# Novel pyrimidine Schiff bases and their selenium-containing nanoparticles as dual inhibitors of CDK1 and tubulin polymerase: design, synthesis, anti-proliferative evaluation, and molecular modelling

**DOI:** 10.1080/14756366.2023.2232125

**Published:** 2023-07-04

**Authors:** Samar El-Kalyoubi, Samiha A. El-Sebaey, Ahmed A. El-Sayed, Moustafa S. Abdelhamid, Fatimah Agili, Sherin M. Elfeky

**Affiliations:** a Department of Pharmaceutical Organic Chemistry, Faculty of Pharmacy, Port Said University, Port Said, Egypt; b Department of Pharmaceutical Organic Chemistry, Faculty of Pharmacy (Girls), Al-Azhar University, Cairo, Egypt; c Photochemistry Department, Chemical Industries Research Institute, National Research Centre, Giza, Egypt; d Department of Biochemistry, Faculty of Science, Zagazig University, Zagazig, Egypt; e Chemistry Department, Faculty of Science (Female Section), Jazan University, Jazan, Saudi Arabia; f Department of Pharmaceutical Organic Chemistry, Faculty of Pharmacy, Mansoura University, Mansoura, Egypt

**Keywords:** Anti-proliferative, cyclin-dependent kinase 1, pyrimidine, selenium nanoparticles, tubulin polymerase

## Abstract

Nanotechnology-based strategies can overcome the limitations of conventional cancer therapies. Hence, novel series of pyrimidine Schiff bases (**4–9**) were employed in the synthesis of selenium nanoparticle forms (**4NPs–9NPs**). All selenium nano-sized forms exerted greater inhibitions than normal-sized compounds, far exceeding 5-fluorouracil activity. Compound **4** showed effective anti-proliferative activity against MCF-7(IC_50_ 3.14 ± 0.04 µM), HepG-2(IC_50_ 1.07 ± 0.03 µM), and A549(IC_50_ 1.53 ± 0.01 µM) cell lines, while its selenium nanoform **4NPs** showed excellent inhibitory effects, with efficacy increased by 96.52%, 96.45%, and 93.86%, respectively. Additionally, **4NPs** outperformed **4** in selectivity against the Vero cell line by 4.5-fold. Furthermore, **4NPs** exhibited strong inhibition of CDK1(IC_50_ 0.47 ± 0.3 µM) and tubulin polymerase(IC_50_ 0.61 ± 0.04 µM), outperforming **4** and being comparable to roscovitine (IC_50_ 0.27 ± 0.03 µM) and combretastatin-A4(IC_50_ 0.25 ± 0.01 µM), respectively. Moreover, both **4** and **4NPs** arrested the cell cycle at G0/G1 phase and significantly forced the cells towards apoptosis. Molecular docking demonstrated that **4** and **4NPs** were able to inhibit CDK1 and tubulin polymerase binding sites.

## Introduction

**Figure 1.**
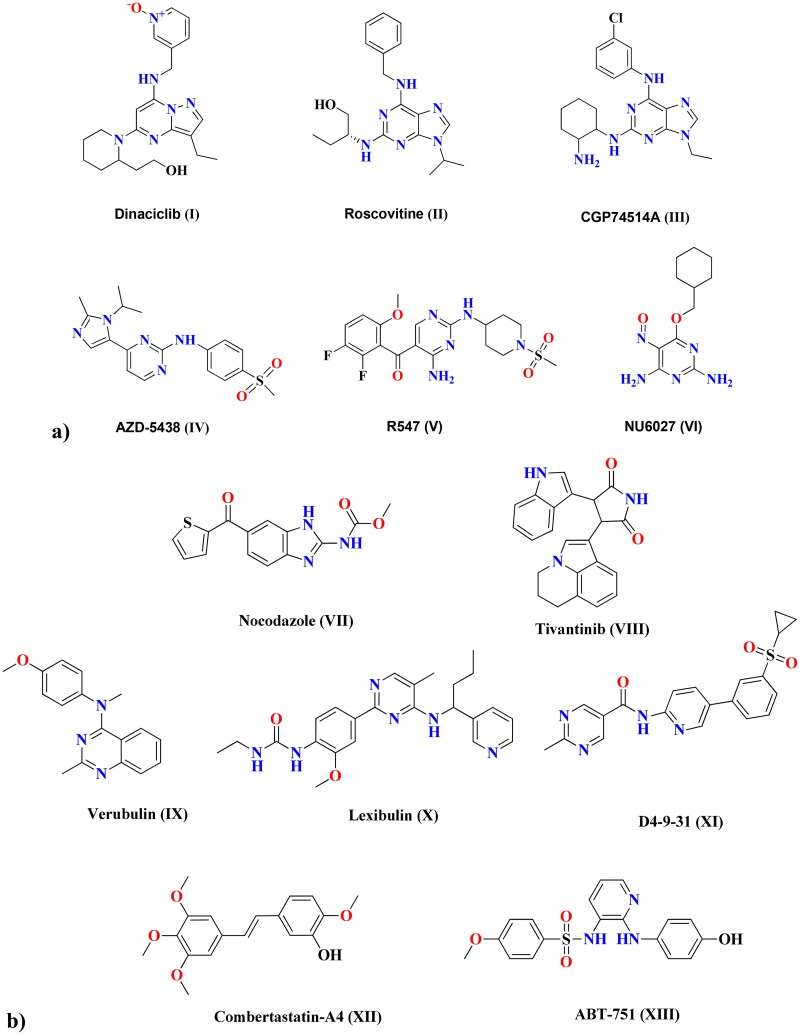
(a) Cyclin-dependent kinase 1 (CDK1) inhibitors. (b) Anti-microtubule and CBSI drugs.

Increased mortality due to cancer is alarming this occurs on account of the ability of cancer cells to develop resistance to anti-proliferative agents in a variety of ways. Combining different drugs that target different pathways (dual inhibitors), as well as designing targeted therapies with high selectivity to cancer cells rather than healthy cells, are key to overcoming this resistance. Metal nanoparticles (NPs) can deliver chemotherapeutic-loaded NPs and are increasingly being used as alternatives to current tumour therapies, including radiotherapy, surgical therapy, and traditional chemotherapy, due to their ability to overcome many of the drawbacks of traditional treatments, such as limited water solubility, non-specific biodistribution, and low bioavailability^1^. One of the most significant characteristics of cancer cells is dysregulated division, which results in aberrant cell growth. Consequently, therapeutic targets that prevent cell proliferation would be successful anti-proliferative agents^2^. Cell cycle regulators known as cyclin-dependent kinases (CDKs) are essential for transcription and differentiation. Catalytic cyclin subunits are responsible for the upregulation of CDK activity, while endogenous CDK inhibitors (CKIs) are responsible for their downregulation. The interaction between cyclin, CDKs, and CKIs is crucial for cell growth. Members of the CDK family play a variety of roles within cells^3^. In particular, CDK1 is important in the mitotic progression of the cell through the S and G2 phases, which depends on the cyclin A-CDK1 complex formation^4^^,^^5^. The development of the cyclin B-CDK1 complex is essential for the cell to proceed through mitosis^6^. Even in the absence of the interphase CDKs (CDK2, 3, 4, and 6), it is possible for CDK1 to drive the necessary events during a cell cycle. This explains why CDK1 inhibitors are essential therapeutic targets when designing anti-proliferative agents. Structurally, CDK1 inhibitors are of various central cores, including; flavones^7^, benzimidazoles^8^, and thiazolones^9^. Furthermore, pyrimidines represent strategic motifs in many CDK1 inhibitors, with high activity and IC_50_ values in the nanomolar range, including dinaciclib (**I**)^10^, roscovitine (**II**)^11^, and CGP74514A (**III**)^12^, as examples of purines and purine isosteres. As well, AZD-5438 (**IV**)^13^, R547 (**V**)^14^, and NU6027 (**VI**)^15^ are other examples of pyrimidines CDK1 inhibitors with potent anti-proliferative activity. Figure 1(a) shows different cyclin-dependent kinase 1 inhibitors that share structural features of a central heterocyclic parent core attached to aromatic hydrophobic moieties *via* different linkers. Microtubules are highly dynamic protein filaments that undergo structural changes during cell division. They assemble the mitotic spindle and carry out modifications needed to advance the cell cycle all the way to the generation of daughter cells. Microtubules are known to be attractive and promising targets for developing of anti-proliferative drugs. New therapeutic approaches are based on the understanding of the regulation and dysregulation of microtubule dynamics and their role in pathological processes^16^. Microtubules and CDK1 are interrelated, where microtubular spindle formation is dependent on the phosphorylation/dephosphorylation of CDK1. During the interphase, the inactive form of CDK1 permits the aggregation α and β-tubulin dimer causing mitotic spindle stabilisation. Once activated at mitosis, CDK1 destabilises microtubulin by inhibiting microtubule-associated proteins, causing the disappearance of mitotic spindles in the cytoplasm^17^. Five exogenous substances were identified as anti-microtubule drugs that act at various binding sites, including vinca alkaloid^18^, maytansine^19^, taxane, laulimalide^20^, and colchicine^21^. Laulimalide, vinca, maytansine, and taxane sites are localised on α-β tubulin heterodimer, while the colchicine site is localised on the tubulin intradimer interface^22^. The colchicine binding site (CBS) has received the most attention in research. Agents that target the β-subunit of curved tubulin and prevent it from acquiring a straight structure can inhibit the assembly of microtubules by interacting at CBS^21^. Figure 1(b) depicts various anti-microtubule and CBSI drugs. Nocodazole (**VII**) and tivantinib (**VIII**) are reported to have binary function inhibitors that act as anti-microtubules through binding at the CBS, as well as target cancer-related kinases^23^. Furthermore, pyrimidine-based verubulin (**IX**)^24^, lexibulin (**X**)^23^, and D4-9-31 (**XI**)^25^ have been found to be potent colchicine binding site inhibitors (CBSIs). Structurally, well-known compounds that bind at the CBS share some common features, including two aryl systems linked *via* different groups. Combretastatin-A4 (**XII**) has two aryls linked *via* an alkene linker, while ABT-751 (**XIII**) has a sulphonamide linker^26^. In the case of verubulin (**IX**), an amino linker connects both aryl systems, while nocodazole (**VII**) has a ketone linker^27^.

**Figure 2.**
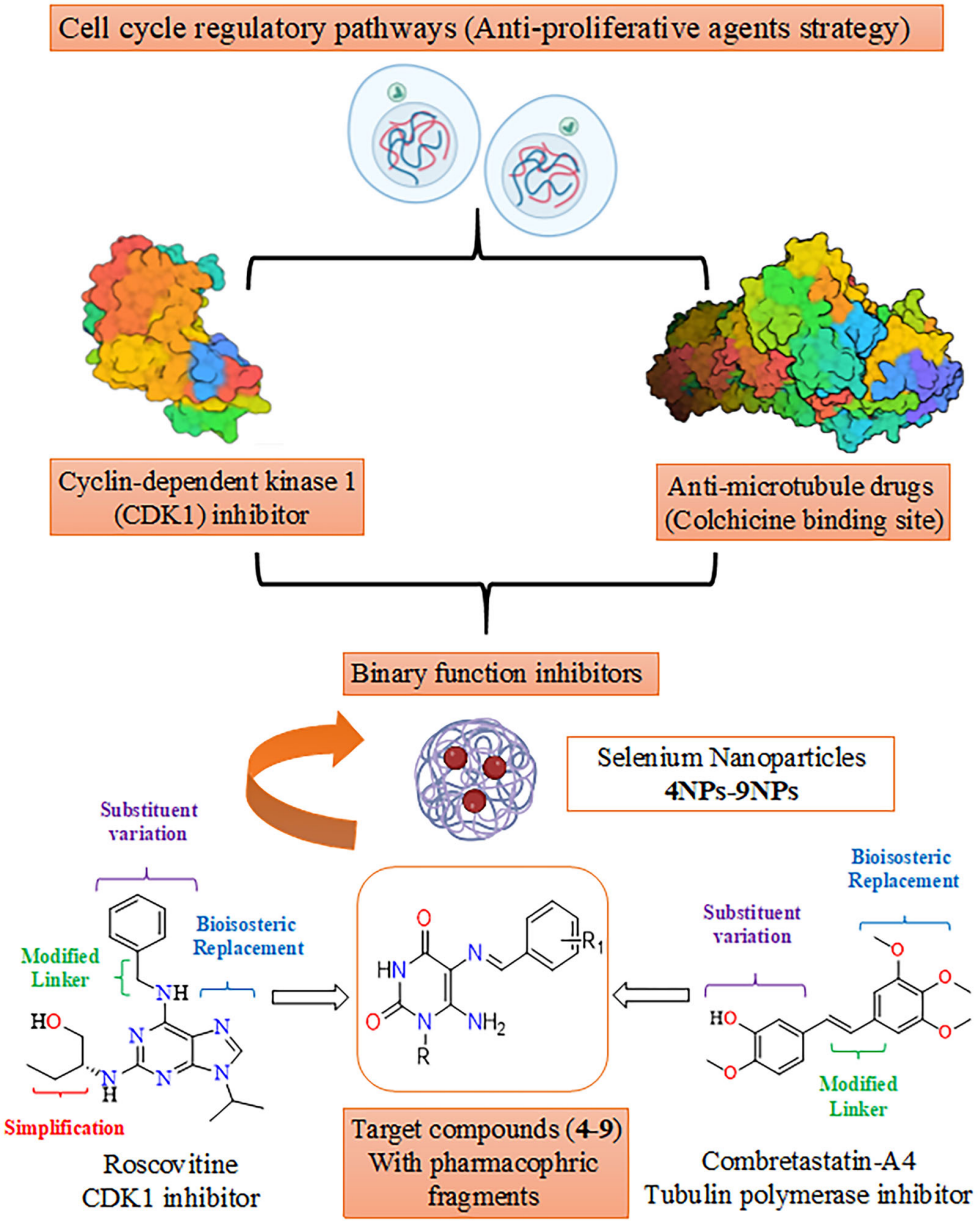
Design of novel pyrimidine Schiff bases and their SeNPs as dual CDK1 and tubulin polymerase inhibitors.

Metal nanoparticles are one of the potential next-generation anticancer therapies as a result of the advancement of nanotechnology in medical research. Selenium (Se) is an essential trace element that is required for many cellular functions *via* selenoprotein incorporation^28^. The significance of selenium nanoparticles (SeNPs) originates from their bioavailability, low toxicity, interaction with proteins, and biocompatibility, particularly when compared to organic and inorganic selenium^29^. SeNPs were demonstrated to have potent anti-proliferative activity^30^ due to their ability to selectively target cancerous cells while having left normal cells undamaged^31^. They are effective in breast, prostate, and lung cancer^32–34^. Compounds containing selenium nanoparticles are reported to regulate a variety of biological functions in cancer cells, including drug delivery, autophagy, and apoptosis^35^. Additionally, SeNP base compounds can inhibit the growth of tumours by blocking the formation of new blood vessels that supply them with nutrients^36^. Furthermore, these compounds can reduce inflammation and oxidative stress, which are both associated with cancer development and progression^31^. On the other hand, unmodified nano-selenium is generally unstable and has a propensity to agglomerate, which significantly lowers its bioavailability^37^. However, many biological macromolecules, including polysaccharides, proteins, and polyphenols, showed the ability to operate as modifiers and regulators for the coating of nano-selenium, thus being candidates for anticancer drugs^38^^,^^39^. Although there is no conclusive evidence of selenium’s ability to prevent cancer, it is essential to reorganise the status of Se and its compounds, whether they are inorganic, organic, or Se-containing nanoparticles, in order to gain a better result of Se’s potential for cancer prevention and treatment^40^. This work aims to design pyrimidine Schiff bases **4–9** as potential dual function CDK1 and microtubule CBS inhibitors. Structural modification of roscovitine (**II**) and combretastatin-A4 (**XII**) was achieved *via* bioisosteric replacement, linker modification, substituent variation, and simplification to afford two aryl systems represented by aminopyrimidine-dione and a substituted phenyl ring connected *via* an imino linker, as shown in Figure 2, as well as their corresponding selenium nanoparticle forms (SeNPs) **4NPs–9NPs**, are being synthesised as a potential alternative chemotherapy strategy. The activity of the synthesised compounds and their SeNPs as anti-proliferative agents will be tested *in vitro* against three cell lines, including the breast cancer cell line (MCF-7), human liver cancer cell line (HepG-2), and lung tumour cell line (A549). In the molecular modelling study, the compounds will be docked into both the enzyme CDK1 and the CBS of microtubule binding sites to compare their interaction modes and binding scores to native ligands. In support of the claim that the designed pyrimidine Schiff bases are inhibitors of both enzymes, CDK1 and tubulin polymerase CBS, the compounds with the greatest inhibitory effects will further be assessed *in vitro* for enzyme inhibition activity. Also, the most active compounds will further be investigated for selective cytotoxicity against Vero cell lines.

## Results and discussion

### Chemistry

**Scheme 1.**
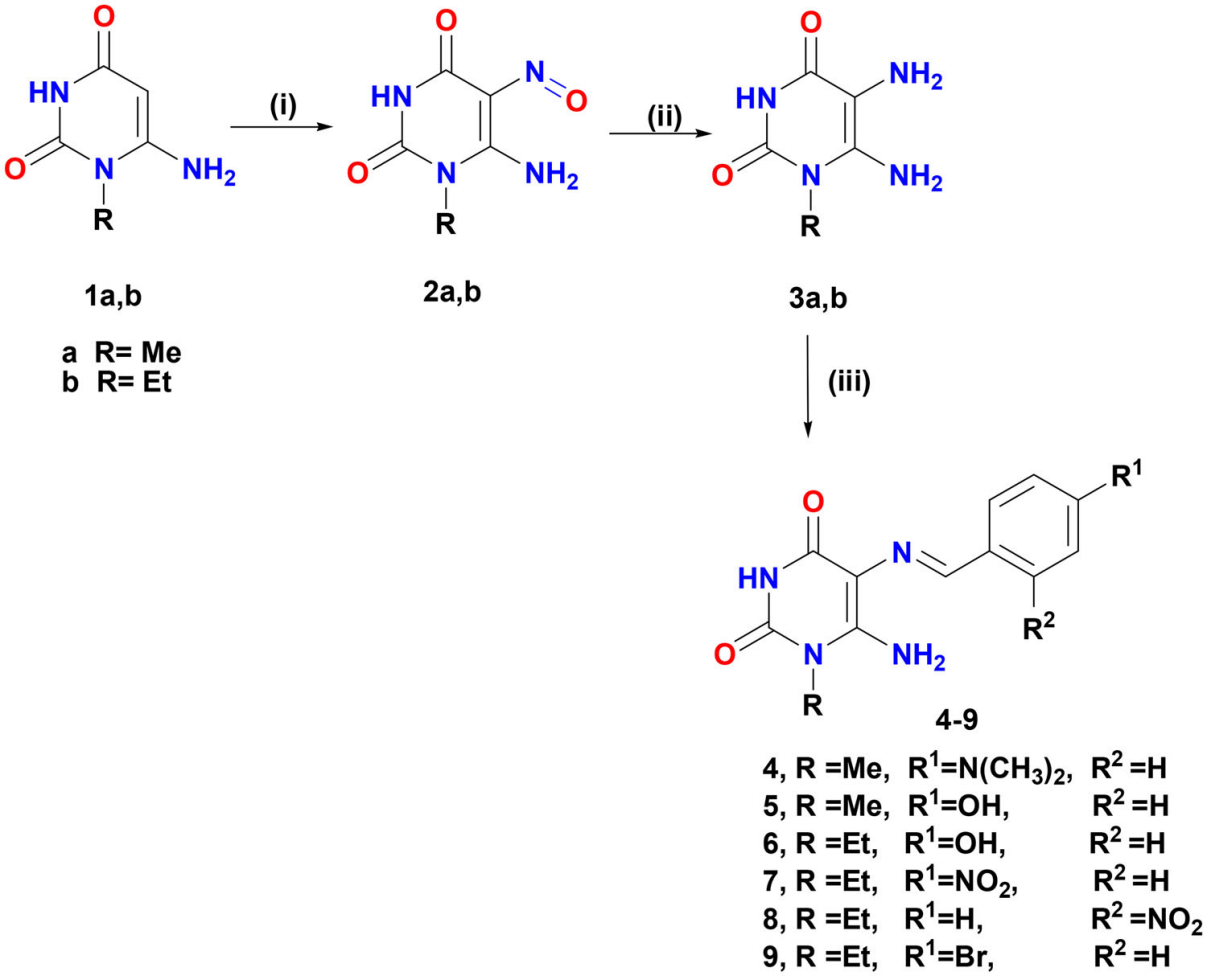
Formation of Schiff′s bases **4–9.** Reagents and conditions: (i) NaNO_2_/AcOH/H_2_O, r.t, 30 min; (ii) (NH_4_)_2_S, 75 °C, 15 min and (iii) aromatic aldehydes/gl. AcOH/heated under fusion, 20 min.

**Figure 3.**
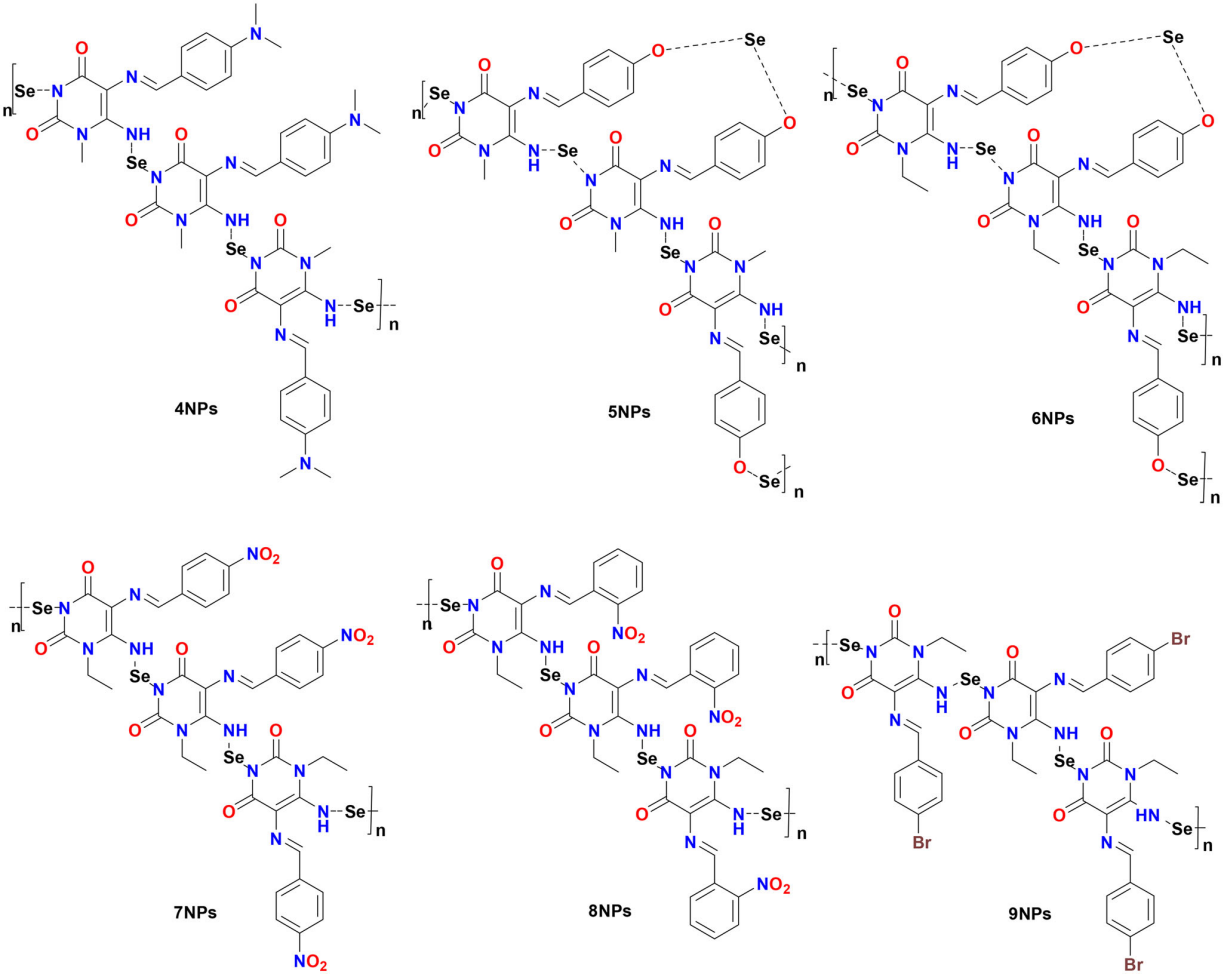
Chemical structures of synthesised Het-SeNPs **4NPs–9NPs**.

The preparation of the target-designed compounds, pyrimidine Schiff bases **4–9**, and their selenium nanoforms **4NPs–9NPs** are outlined in Scheme 1 and Figure 3, respectively.

#### Synthesis of pyrimidine Schiff bases 4–9

5,6-Diaminouracils (**3a, b**) were utilised as a starting substrate for the synthesis of Schiff’s bases. These compounds were prepared by nitrosation of 6-aminouracil (**1a, b**) in a stirred aqueous solution of sodium nitrite in glacial acetic acid at room temperature, affording **2a, b.** The latter was then reduced by the reducing agent ammonium sulphide to furnish the starting material **3a,b**^41–46^. Pyrimidine Schiff bases **4–9** were formed under solvent-free conditions with excellent yields (84–94%) in a short time by condensation of the primary amine of 5,6-diaminouracil (**3a, b**) with a variety of aromatic aldehydes, including 4-(dimethylamino)benzaldehyde, 4-hydroxybenzaldehyde, 4-nitrobenzaldehyde, 4-bromobenzaldehyde, and 2-nitrobenzaldehyde. The reaction was carried out in an acidic medium, glacial acetic acid, to protonate the unstable carbinolamine intermediate and catalysed dehydration *via* water loss, giving the Schiff’s base products. When compared to other approaches, the current approach is considered green and eco-friendly for the production of various Schiff’s bases due to the use of accessible starting materials, solvent-free conditions, and simple product isolation, as well as high yields. These compounds were established by full spectroscopic data and elemental analysis. The ^1^H NMR of Schiff’s bases **4–9** was devoid of δ values of C5–NH_2_ and confirmed the presence of NH-3 and C6–NH_2_ protons in the range of δ 10.62–10.82 and 7.12–7.62 ppm, respectively, that were disappeared by D_2_O. Also, their ^1^H NMR demonstrated singlet signals ascribed to azomethine proton at δ 9.54–9.89 ppm, as well as their ^13^C NMR revealed a distinctive signal for azomethine carbon between δ 157.89 and 159.12 ppm. Both the ^1^H NMR and ^13^C NMR spectra clearly showed the presence of additional protons and carbon signals.

#### Synthesis of heterocyclic selenium nanoparticles 4NPs–9NPs in situ using pyrimidine Schiff bases (4–9):

Nanoparticles have a higher surface area to volume ratio than micro- or sub-micrometer-sized particles, which can be used to bind significant amounts of either active/targeting biomolecules (enzymes, proteins, DNA) or chemical compounds (chemotherapeutic agents). Therefore, the team’s work aimed to create new selenium nanoparticles (SeNPs) using the synthesised pyrimidine Schiff bases (**4–9**), which have appropriate low reducing but high stabilising properties during SeNPs preparation. The organic heterocycle derivatives reduced the Se^+^ cation to Se^0^ using ascorbic acid as a catalyst that acts as an aldehyde to form SeNPs and stabilise the nano-structure of SeNPs^47^^,^^48^. The organic compounds with reductive groups, including -OH, -NH, -NH_2_, and others, could reduce selenium cation. The structure of the synthesised **4NPs–9NPs** is presented in Figure 3. Selenium nanoparticle compounds (**4NPs–9NPs**) were confirmed by UV-Spectrophotometer, TEM technique, and particle size distribution.

##### Ultraviolet-visible (UV-Vis) spectra

**Figure 4.**
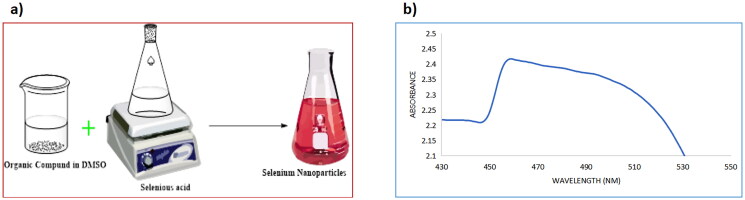
(a) A schematic diagram of the synthesis of selenium nanoparticles; (b) UV-Vis spectrum of selenium nanoparticles.

The first indication of the formation of nanoparticles was the solution turning red after adding H_2_SeO_3_ to Schiff bases (**4–9**) dissolved in DMSO and stirring for 1 h at 60 °C, as shown in Figure 4(a). The formation of selenium nanoparticles **4NPs–9NPs** was monitored using UV-Vis spectroscopy. The UV-Vis absorption spectrum of selenium nanoparticles is depicted in Figure 4(b). The colloidal solution of selenium exhibits an absorption peak at 530 nm corresponding to the surface plasmon resonance peaks in the absorption spectra.

##### Transmission electron microscope (TEM)

**Figure 5.**
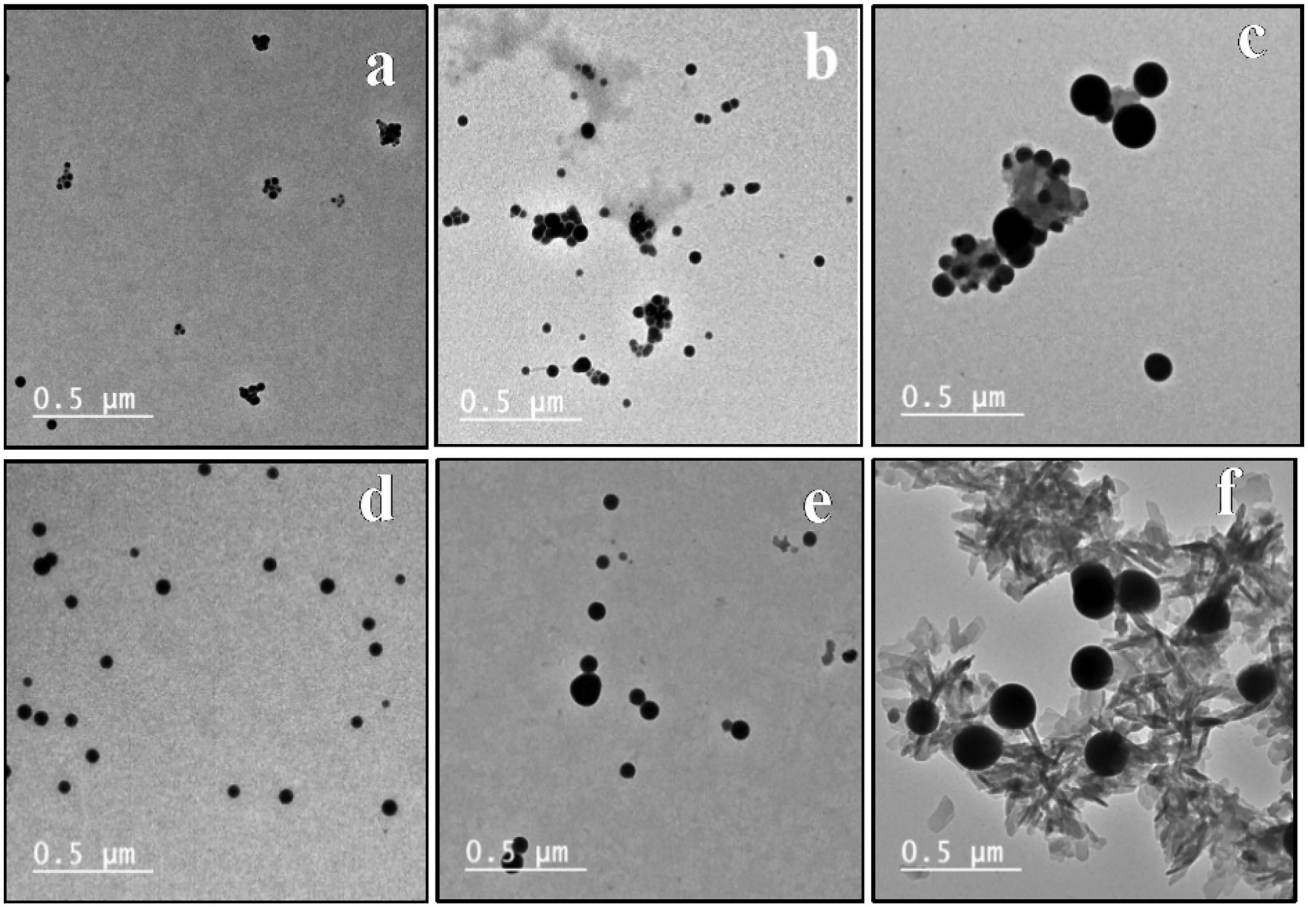
TEM of SeNPs, (a), (b), (c), (d), (e), and (f) TEM of SeNPs loaded onto **4–9**, respectively.

Transmission electron microscopy (TEM) confirmed the characterisation of selenium colloidal nanoparticles dispersed in an aqueous solution without the formation of aggregations^49^. TEM was used to examine the prepared selenium Nanoparticles, either alone or loaded onto the synthesised pyrimidine Schiff bases (**4–9**). The presence of formed selenium nanoparticles in the tested specimens was illustrated in Figure 5. Selenium nanoparticles are found in spherical shapes with minor aggregate, and their particle sizes range from 24.98 to 60.78 nm. When selenium nanoparticles are loaded onto the investigated compounds **4–9,** they spread uniformly throughout both samples, with the nanoparticles encompassing the organic heterocyclic compounds, as graphically illustrated in Figure 5(a–f). The synthesised Schiff bases contributed to the prevention of agglomerates in selenium nanoparticles. It appears that the nitrogen-based compounds’ stabilising effect contributed to the existence of Se nanoparticles separated from each other with convenient distributions^50^^,^^51^.

##### Size distribution and zeta potential

Dynamic light scattering (DLS) measurements were used to determine the particle size of the synthesised **4NPs–9NPs**. The particle size illustrated in Figure S7 in the Supplementary File has an average of 50.43 to 80.90 nm. The reason for the large particle size, which is obtained by DLS rather than TEM measurement, is due to the fact that TEM provides an image for a specific measurement range, whereas DLS provides an overall image of nanoparticles and their aggregations. In addition, DLS denotes the hydrodynamic radius of nanoparticles (hydrated particles) or coated particles in an aqueous solution, while TEM determines the dried diameter of nanoparticles^52^. Furthermore, the Polydispersity Index (PDI) of the SeNPs system changed in the range of 0.013 to 0.666 after interaction with heterocyclic compounds, indicating significant changes in the nanoparticle size distribution, as depicted in Figure S8 of the Supplementary File. Moreover, the zeta potential of untreated SeNPs was reported to be −30.2 mV^53^, which increased in the range of −17.3 to +8.15 mV due to the adsorption of positively-charged heterocyclic compounds molecules onto the matrix of SeNPs (Figure S9 in the Supplementary File). This indicates that electrostatic interactions played an important role in the formation of heterocyclic derivatives-loaded nanoparticles. It is worth noting that zeta potential is a key indicator of colloidal suspension stability. As a result, the negative charge of the heterocyclic derivatives-loaded nanoparticles indicates that the whole system is stable^54^. Table 1 shows the Mean Particle Size (MPS), PDI, mean SD, and zeta potential of heterocyclic-selenium nanoparticles **4NPs–9NPs.**

**Table 1.** Particle size and zeta potential of Het-SeNPs **4NPs–9NPs**.

Compounds no.	MPS (nm)	PDI	SD	Zeta potential
**4NPs**	80.9	0.336	±5.876	7.70
**5NPs**	58.55	0.666	±8.414	8.15
**6NPs**	75.96	0.202	±7.324	0.229
**7NPs**	50.43	0.013	±5.143	−1.51
**8NPs**	78.22	0.033	±6.653	−17.30
**9NPs**	60.47	0.626	±7.987	−0.948

### Biological evaluation

#### In vitro cytotoxicity assay

In general, as shown in Table 2, it was noticed that the selenium nano-sized pyrimidine Schiff bases (**4NPs–9NPs**) exhibited a more potent inhibitory effect than normal-sized pyrimidine Schiff base candidates (**4–9**) on human cancer cell lines, much outperforming the activity of clinically used standard 5-fluorouracil. For pyrimidine Schiff base candidates (**4–9**), the most effective inhibitory activity was observed for compounds **4-** and **6-**bearing dimethylamino and hydroxyl functionalities, respectively – when compared to other members of the series. **4NPs** achieved an efficacy that exceeds its normal-sized form (**4**) by 98.74%, 97.20%, and 97.38% (*p* ≤ 0.001) when introduced into MCF-7, HepG-2, and A569 cell lines, respectively. As well, **6NPs** showed a significant increase in efficiency by 96.52%, 96.45%, and 93.86% against MCF-7, HepG-2, and A569, respectively, when compared to its normal-sized form (**6**). Based on the aforementioned findings, candidates **4** and **6** and their nanoforms were selected for further biological estimations.

**Table 2.** The anti-proliferative activity of pyrimidine Schiff bases (**4–9**) and their nano-sized forms (**4NPs–9NPs)** against human cancer cell lines (IC_50_ µM).

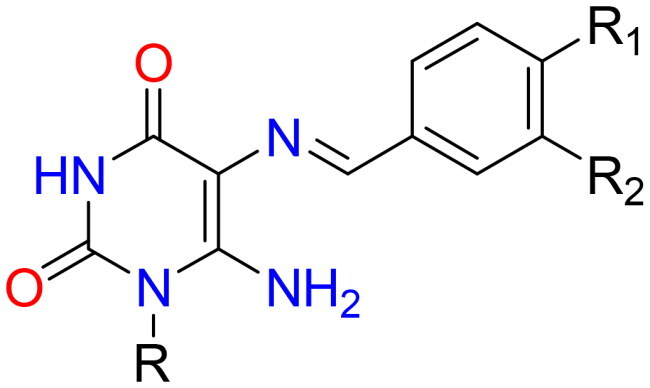							
Ser. no.	Compounds no.	IC_50_ VALUE (µM ± SD)^a^					
		R	R^1^	R^2^	MCF-7	HepG-2	A549
1	**4**	Me	N(CH_3_)_2_	H	3.17 ± 0.04	1.07 ± 0.03	1.53 ± 0.01
2	**5**	Me	OH	H	3.59 ± 0.08	4.07 ± 0.08	4.27 ± 0.08
3	**6**	Et	OH	H	1.15 ± 0.03	1.97 ± 0.05	1.14 ± 0.04
4	**7**	Et	NO_2_	H	3.37 ± 0.04	3.38 ± 0.11	4.16 ± 0.07
5	**8**	Et	H	NO_2_	2.03 ± 0.07	2.1 ± 0.09	2.3 ± 0.05
6	**9**	Et	Br	H	3.16 ± 0.13	3.47 ± 0.09	3.43 ± 0.05
7	**4NPs**	Me	N(CH_3_)_2_	H	0.04 ± 0	0.03 ± 0.01	0.04 ± 0
8	**5NPs**	Me	OH	H	0.06 ± 0	0.09 ± 0	0.07 ± 0
9	**6NPs**	Et	OH	H	0.04 ± 0	0.07 ± 0	0.07 ± 0
10	**7NPs**	Et	NO_2_	H	0.07 ± 0	0.03 ± 0	0.04 ± 0
11	**8NPs**	Et	H	NO_2_	0.04 ± 0	0.05 ± 0	0.07 ± 0
12	**9NPs**	Et	Br	H	0.07 ± 0	0.05 ± 0	0.1 ± 0.01
13	**SeNPs**	–	–	–	0.03 ± 0	0.04 ± 0	0.04 ± 0
14	**5-FU**				1.82 ± 0.03	1.36 ± 0.01	0.76 ± 0.04

^a^IC_50_ value is the concentration of the test compound required to inhibit the growth of 50% of cancer cell population. Values represent the mean for three experiments performed in triplicate.

To determine the selectivity of compounds **4** and **6** and their nanoforms **4NPs** and **6NPs**, these candidates were screened against HepG-2 and normal Vero cell lines. The selectivity of nano-sized forms of both compounds was comparable to that of 5-fluorouracil. Treatment of cell lines with **4NPs** and **6NPs** achieved a significant increase in selectivity by 8.78-fold and 2.24-fold, respectively, when compared with the reference drug, which was attributed to the increased bioavailability of these compounds. It was also observed that compounds **4** and **4NPs** were more selective than compounds **6** and **6NPs** by 2.21-fold and 3.93-fold, respectively (Table 3). Therefore, compounds **4** and **4NPs** were selected for evaluating cell cycle analysis and apoptotic-inducing effect.

**Table 3.** The selectivity of the most potent candidates **4** and **6** in both normal and nanoformulations (**4NPs** and **6NPs**).

Ser. no.	Compounds no.	Cytotoxicity (IC_50_ µM ± SD)		
		HepG-2	Vero	SI^a^
1	**4**	1.07 ± 0.03	4 ± 0.05	3.74
2	**6**	1.97 ± 0.05	3.34 ± 0.06	1.69
3	**4NPs**	0.03 ± 0.01	0.59 ± 0.01	19.67
4	**6NPs**	0.07 ± 0	0.35 ± 0.01	5.00
5	**5-FU**	1.36 ± 0.01	3.04 ± 0.06	2.24

^a^Selectivity index = IC_50_ normal cell/IC_50_ cancer cell.

#### In-vitro inhibitory activity of CDK1 and tubulin polymerisation CBS

**Figure 6.**
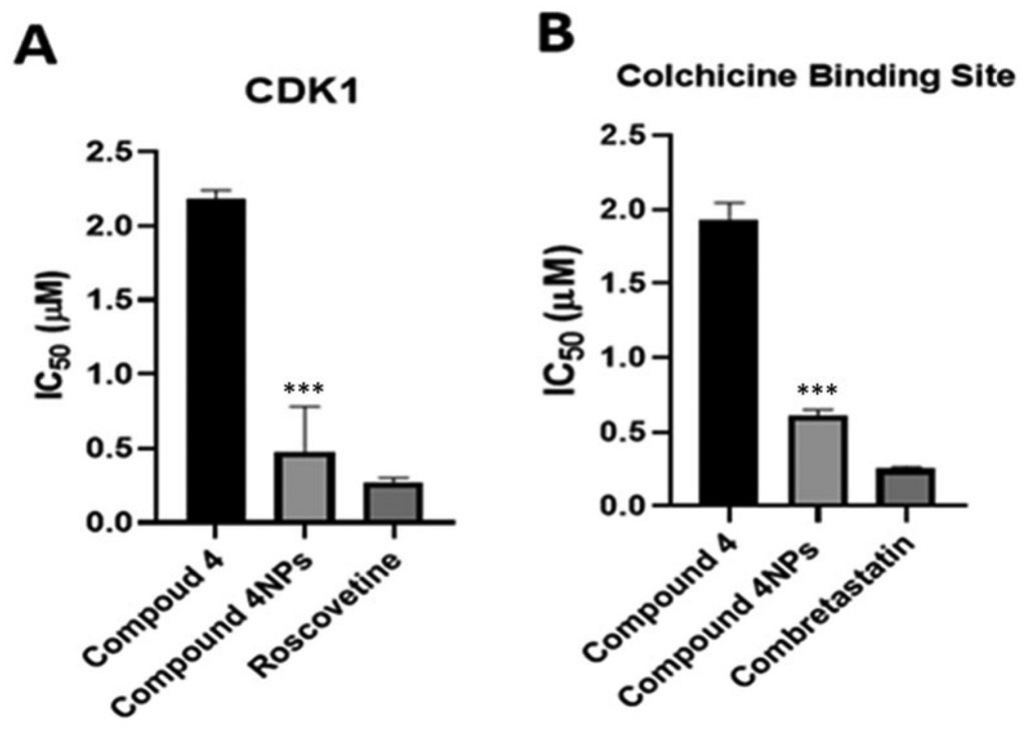
The inhibitory effect of the compounds **4** and **4NPs** on (a) cyclin-dependent kinase 1, (b) Tubulin polymerisation through colchicine binding site. (*) indicate to the significant differences between compound **4NPs**-treated and compound **4**-treated cells, where (***) indicates to *p* < 0.001. All experiments were performed in triplicates.

It was noticed that the inhibitory activity of nanoformulation **4NPs** against CDK1 was significantly increased by 4.6-fold compared to compound **4**. **4NPs** showed CDK1 inhibitory effect approaching that of roscovitine (Table 4 and Figure 6(a)). Compound **4** and its nanoformulation (**4NPs**) exerted a colchicine binding site inhibitory (CBSI) activity as well, evidenced by IC_50_ values. The inhibitory effect of the nanoformulation of compound **4** was comparable to that of combretastatin-A4. Based on our findings, the incorporation of nanoparticles into the test compound maximised its efficacy as a tubulin assembly destabilising agent, thus a mitotic inhibitor (Table 4 and Figure 6(b)).

**Table 4.** The inhibitory activity of the most potent candidates **4** and **4NPs** against both CDK1 and tubulin polymerase (CBS) compared to two reference drugs.

Ser. no.	Compounds no.	CDK1 (IC_50_ µM ± SD)	Tubulin polymerase (CBS) (IC_50_ µM ± SD)
1	**4**	2.183 ± 0.06	1.926 ± 0.119
2	**4NPs**	0.475 ± 0.31	0.614 ± 0.038
3	**Roscovitine**	0.273 ± 0.03	–
4	**Combretastatin-A4**	–	0.25 ± 0.015

#### Flow cytometric analysis of cell-cycle distribution

**Figure 7.**
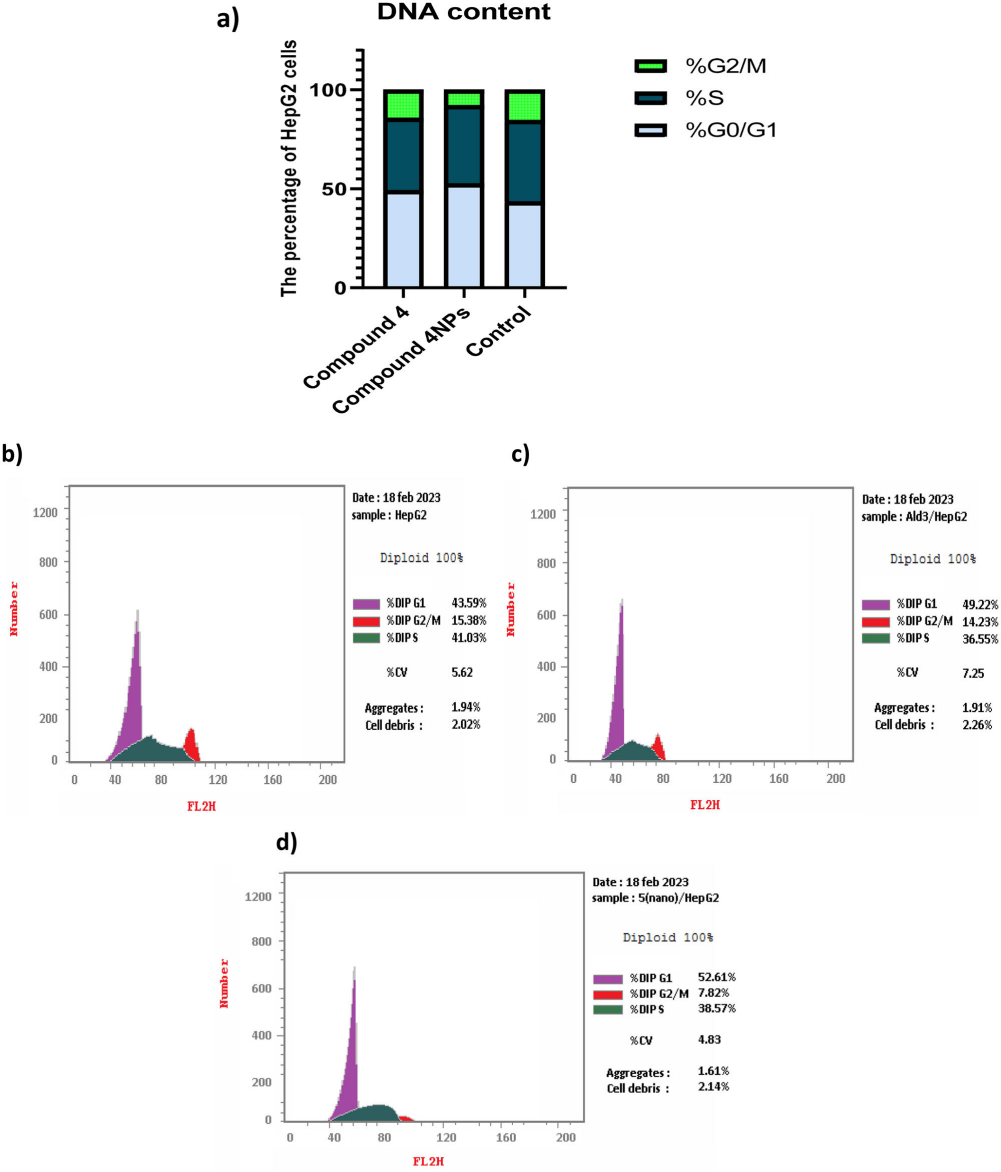
(a) The effect of the most potent compounds **4** and **4NPs** on cell cycle phases of HepG-2. Flow cytometric histograms of HepG-2 cell cycle phases; (b) untreated cells, (c) treated with compound **4** alone, and (d) treated with compound **4NPs**. All experiments were performed in triplicate.

Flow cytometric analysis of the cell cycle demonstrated that HepG-2 cells, which were found to be the most affected in cytotoxicity screening, were arrested at the G1 phase when treated with both **4** and nanoformulation **4NPs**. This arrest was evidenced by the accumulation of cells at G0/G1 as well as a decrease in the percentage of cells in the S phase by 49.15% in comparison with untreated cells, as displayed in Table 5 and Figure 7(a). Compared to **4**, the nanoformulation **4NPs** caused a significant rise in the proportion of cells arrested at G0/G1, indicating enhancement of the efficiency of the test compound. Figure 7(b–d) shows cytometric histograms of HepG-2 cell cycle phases.

**Table 5.** The effect of the most potent candidates **4** and **4NPs** on cell cycle phases of HepG-2.

Ser no.	Compounds no.	%G0–G1	%S	%G2/M
1	**4 /HepG-2**	49.22 ± 2.61	36.55 ± 1.93	14.23 ± 0.81
2	**4NPs/HepG-2**	52.61 ± 2.72	39.57 ± 4.01	7.82 ± 1.02
3	**Cont.HepG-2**	43.59 ± 2.66	41.03 ± 3.42	15.38 ± 0.77

#### Cellular apoptosis analysis

**Figure 8.**
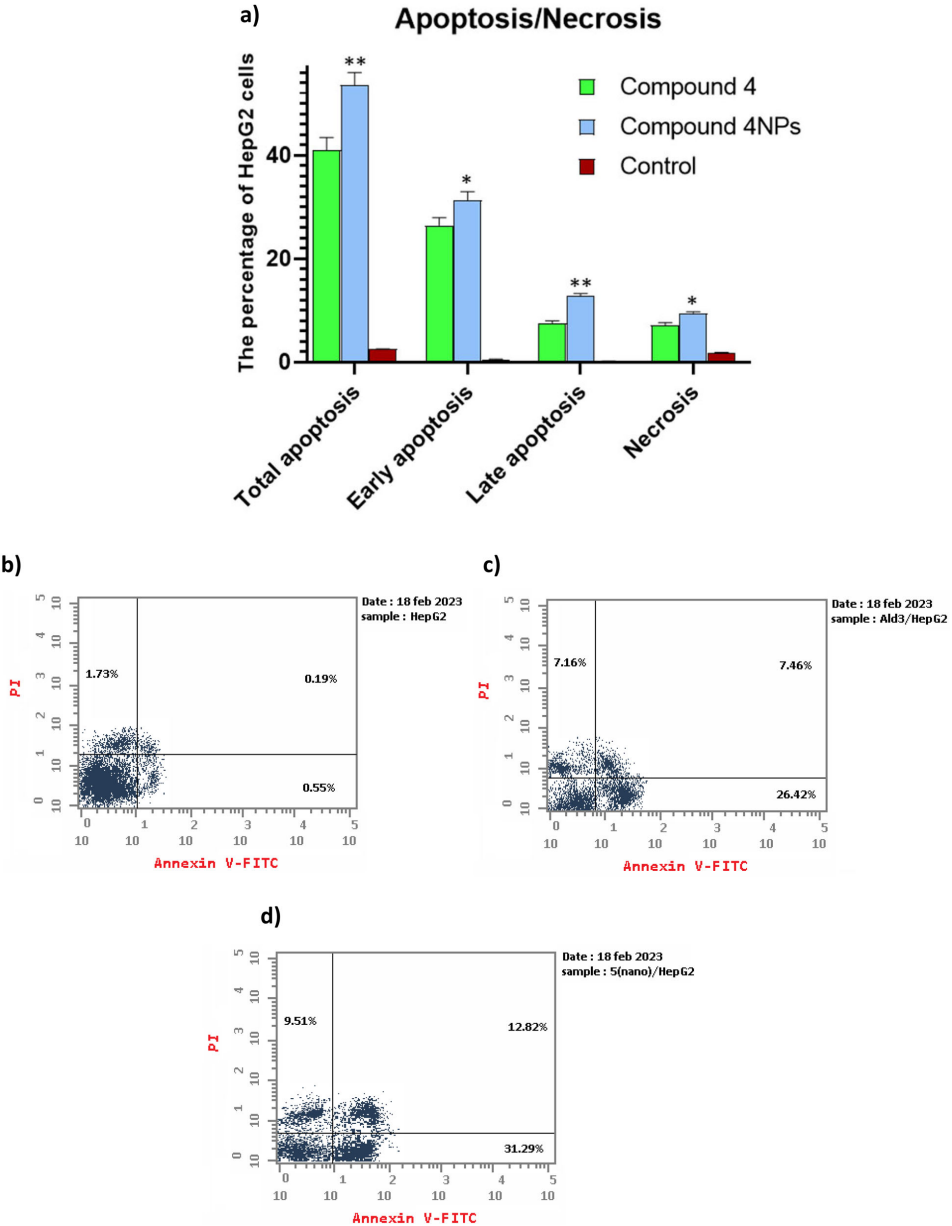
(a) The apoptosis-inducing effect of the most potent candidates **4** and **4NPs** on HepG-2. (*) indicate to the significant differences between compound **4NPs**-treated and compound **4**-treated cells, where (*) indicates to *p* < 0.05, (**) *p* < 0.01. All experiments were performed in triplicates. (b–d). Flow cytometric dot plot of PI/annexin V screening of HepG-2; (b) untreated cells, (c) cells treated with compound **4**, and (d) cells treated with compound **4NPs**. All experiments were performed in triplicate.

Screening of HepG-2 cells using PI/Annexin V clarified that compounds **4** and **4NPs** forced the cells towards apoptosis significantly. The most powerful apoptotic effect was obtained when compound **4NPs** was used, as evidenced by a remarkable increase in the percentage of apoptotic cells approaching 31% when compared to that treated with compound **4**. Furthermore, the test compounds **4** and **4NPs** increased the percentage of necrotic cells by 5.50- and 4.14-fold, respectively, compared to untreated cells (Table 6 and Figure 8(a–d)). Our findings suggested that the transformation of compound **4** to nano-sized form **4NPs** increased its apoptotic-inducing potency, accordingly killing cancer cells.

**Table 6.** The apoptotic effect of the most potent candidates **4** and **4NPs** on HepG-2.

Ser. no.	Compound no.	Apoptosis			Necrosis
		Total	Early	Late	
1	**4 /HepG-2**	41.04 ± 2.39	26.42 ± 1.47	7.46 ± 0.54	7.16 ± 0.44
2	**4NPs/ HepG-2**	53.62 ± 2.42	31.29 ± 1.72	12.82 ± 0.44	9.51 ± 0.27
3	**Cont.HepG-2**	2.47 ± 0.07	0.55 ± 0.04	0.19 ± 0.02	1.73 ± 0.14

### Molecular docking simulation

**Figure 9.**
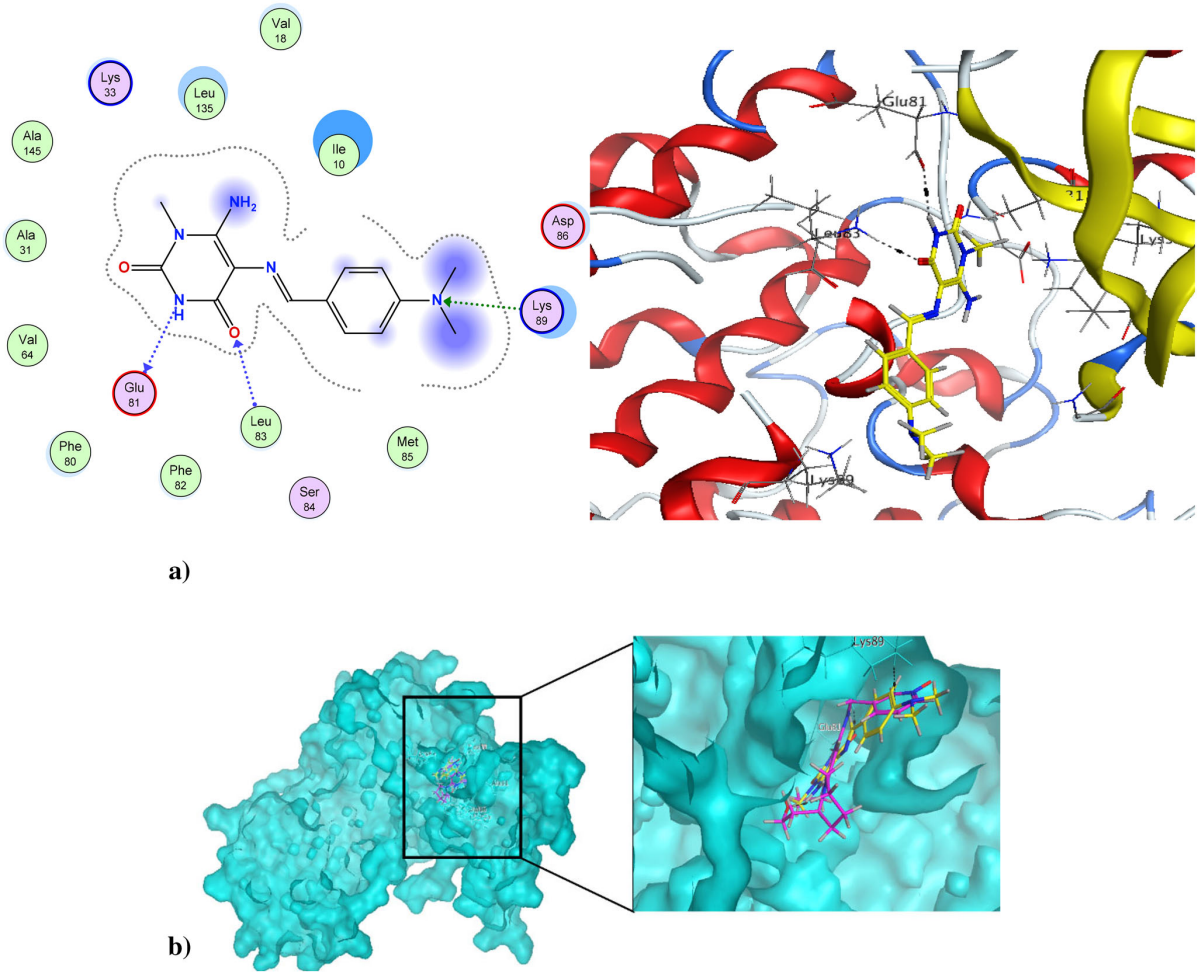
(a) Compound **4** at the active site of CDK1 (PDB ID: 6GU6), and (b) alignment of compound **4** and dinaciclib at the active site of CDK1 (PDB ID: 6GU6).

**Figure 10.**
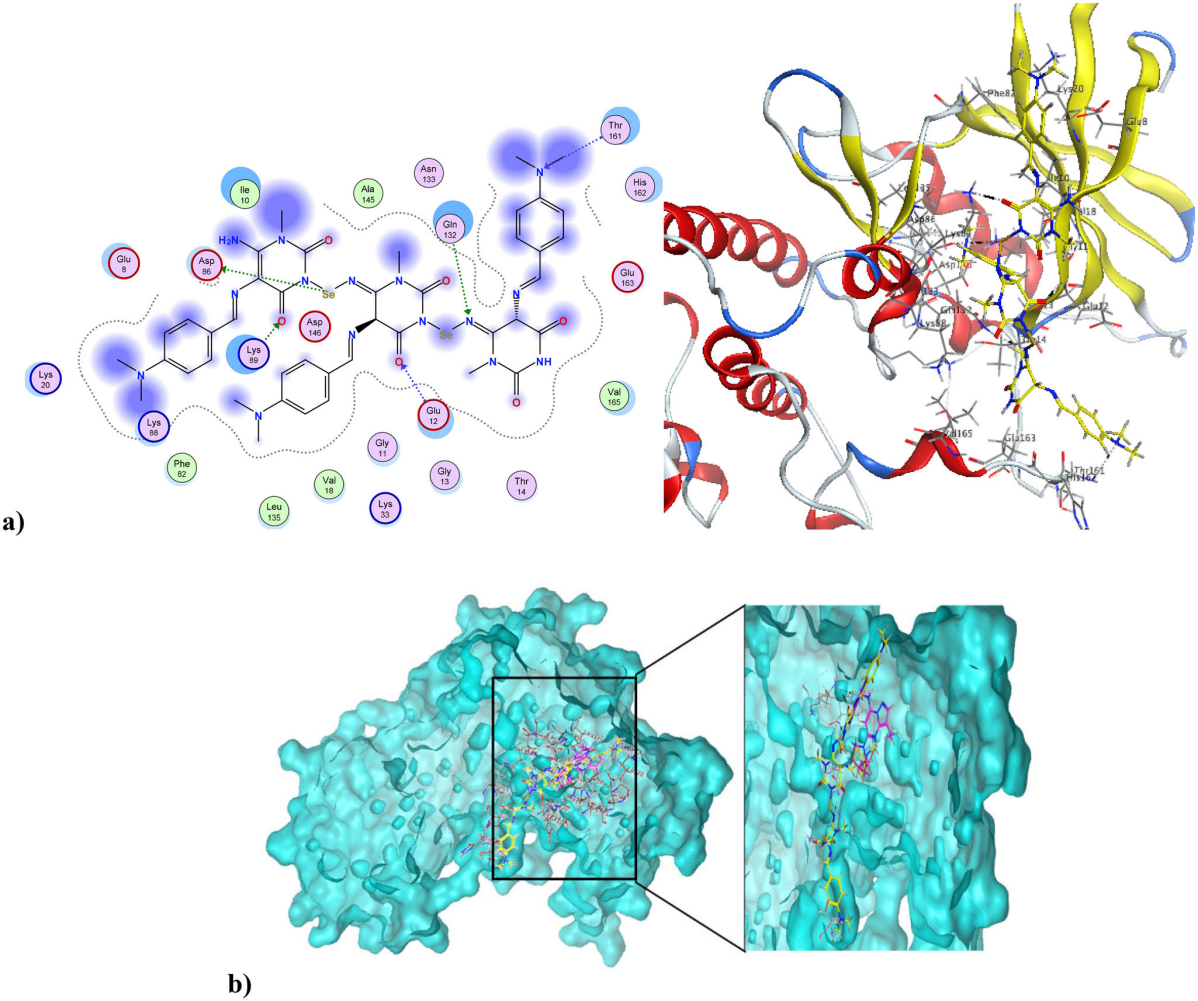
(a) Nano compound **4NPs** at the active site of CDK1 (PDB ID: 6GU6). (b) The alignment of **4NPs** and dinaciclib at the active site of CDK1 (PDB ID: 6GU6).

Cancer is associated with uncontrolled cell proliferation that results in most cases from dysregulation of the cell cycle^55^. This dysregulation is often characterised by an increase in the level of cyclin-dependent kinases and their catalytic cyclin subunits or a decrease in endogenous cyclin kinase inhibitors^56^^,^^57^. This explains why CDKs are such attractive therapeutic targets for designing potent and selective anti-proliferative agents. CDK1, like all its family, is characterised by a conserved protein kinase domain with a small *N*-terminal lobe and a large C-terminal fold that are connected by a hinge. Dinaciclib is an ATP-competitive CDK inhibitor that can bind to cyclin-free CDK1 [58]. As shown in Figure S10 in the Supplementary File, dinaciclib binds at the active site of CDK1 *via* three types of interactions. The first is an *H*-bond interaction formed by the NH linker at the key amino acid Leu83, while the second is a hydrophobic interaction formed by the core pyrazole moiety of dinaciclib with the two lobes at amino acids Val18, Leu135, and Ile10. The third type of interaction is achieved *via* stacking interaction of the pyridine moiety of dinaciclib against the key amino acid Ile10. All these pull the amino acids closer to the inside of the active site, narrowing it and resulting in the inhibitory activity of dinaciclib. The newly synthesised compound **4** was capable of binding at the CDK1 active site, exerting a docking score of −6.25 K.cal/mol compared to the docking score of −8.46 K.cal/mol of dinaciclib. Figure 9(a) depicts compound **4** at the CDK1 active site (PDB ID: 6GU6). Compound **4** formed an *H*-bond interaction with the key Leu83 amino acid through its carbonyl at position-4 of the pyrimidinedione ring system. It formed an additional hydrogen bond interaction at Gly81 *via* NH at position-3 of the pyrimidinedione. Similar to dinaciclib, it exhibited hydrophobic interaction at key amino acids Val18, Leu135, and Ile10. Figure 9(b) shows the alignment of **4** and dinaciclib at the CDK1 active site (PDB ID: 6GU6). The nano form of **4**, compound **4NPs** was docked into the CDK1 active site, exhibiting a docking score of −9.36 K.cal/mol. It was capable of binding at the active site, where it formed several *H*-bond interactions with different amino acids, including Asp86, Lys89, Gly12, Thr161, and Gln132. Also, it formed hydrophobic interactions with key amino acids such as Ile10, Lys89, Val18, Leu135, Phe82, and Gln132, as illustrated in Figure 10(a). Additionally, the alignment of **4NPs** and dinaciclib at the CDK1 active site (PDB ID: 6GU6) is displayed in Figure 10(b).

**Figure 11.**
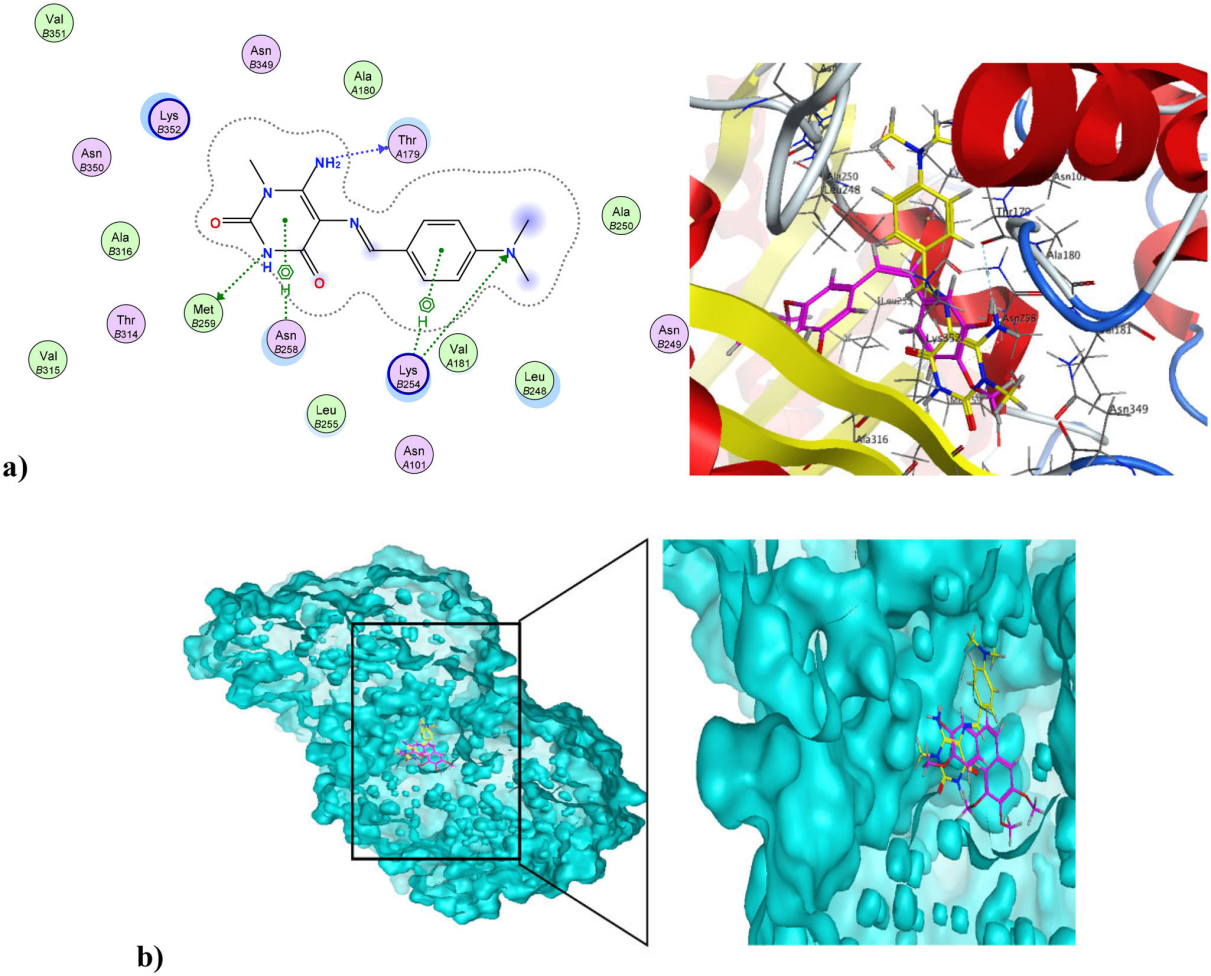
(a) Compound **4** at the CBS of microtubules (PDB ID: 5LYJ). (b) The alignment of compound **4** and combretastatin-A4 at the CBS of microtubules (PDB ID: 5LYJ).

**Figure 12.**
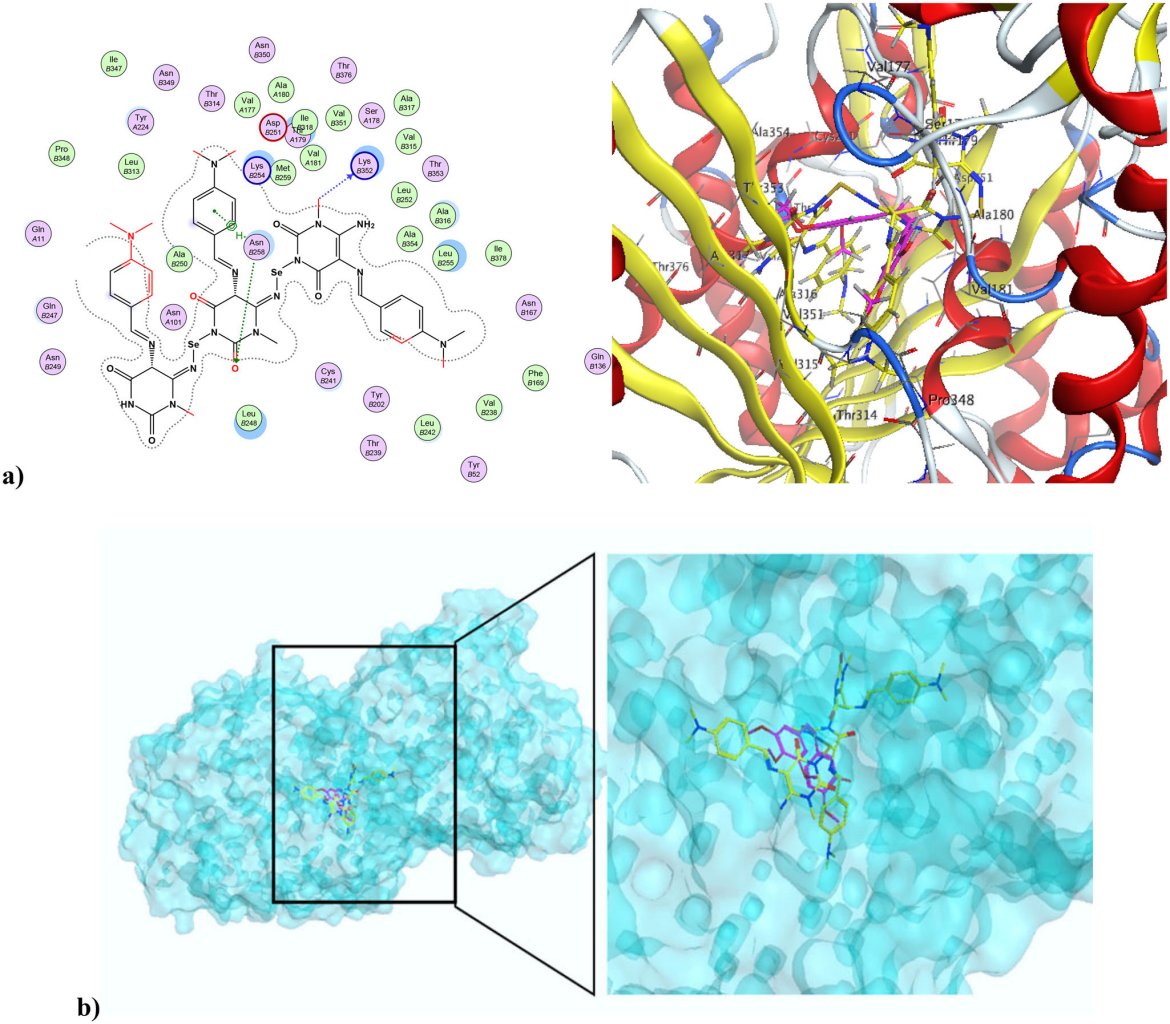
(a) Nano compound **4NPs** at the CBS of microtubules (PDB ID: 5LYJ). (b) The alignment of nano compound **4NPs** and combretastatin-A4 at the CBS of microtubules (PDB ID: 5LYJ).

Microtubules play vital roles in cellular processes, where they help maintain cell shape. They participate in cellular division through the formation of the mitotic spindle. Microtubule targeting agents, also known as microtubule destabilising agents, are potent anti-proliferative agents^23^^,^^59^^,^^60^. Combretastatin-A4 is a diphenyl derivative with a high potency of disrupting the cell cycle as well as angiogenic activities^61^^,^^62^. Combretastatin-A4 can inhibit microtubules through binding at the colchicine binding site (CBS), resulting in disruption of cancer cell growth and powerful cytotoxic activity. The nature of combretastatin A4 interaction at the colchicine binding site (CBS) is mainly hydrophobic, with hydrophobic interactions with many key amino acids at the pocket, including Asn352, Asn349, Leu255, Leu248, Val238, Cys241, and Ala316. It forms *H*-bond interactions at Thr179 and Val238. Figure S11 in the Supplementary File shows combretastatin-A4 at the colchicine binding site (CBS) of microtubules (PDB ID: 5LYJ)^63^. The newly synthesised compound **4** was capable of interacting at CBS of microtubules in a mode similar to combretastatin-A4, showing a docking score of −7.79 K.cal/mol, which was comparable to combretastatin A4’s docking score of −8.54 K.cal/mol. The pyrimidinedione of compound **4** aligned with the di-substituted phenyl ring of combretastatin A4. Compound **4** formed three *H*-bond interactions at Thr179, Met259, and Lys254. Also, it formed Arene-Hydrogen interactions at Lys254 and Asn258. Additionally, it showed hydrophobic interactions with a number of amino acids at the colchicine binding site, including Lys254, Asn254, and Lys352. Figure 11(a) shows compound **4** at the CBS of microtubules (PDB ID: 5LYJ), while Figure 11(b) depicts the alignment of compound **4** and combretastatin A4 at the CBS of microtubules (PDB ID: 5LYJ). On the other hand, compound **4NPs**, the nano form of **4**, was also capable of interacting at CBS, showing a docking score of −9.27K.cal/mol. Figure 12(a) illustrates that the nano compound **4NPs** formed *H*-bond interactions at Lys352 and Asn258. Similar to combretastatin -A4, nano compound **4NPs** formed hydrophobic interactions with many amino acids, including Leu255, Lys352, Lys254, Thr179, Leu248, Gln247, Gln11, and Ala316. Figure 12(b) shows the alignment of nano compound **4NPs** and combretastatin- A4 at the CBS of microtubules (PDB ID: 5LYJ).

## Conclusion

Selenium-based nanoparticles are believed to improve tumour targeting of chemotherapeutic agents, resulting in more effective tumour inhibition. Thus, the pyrimidine Schiff bases (**4–9**) were synthesised under solvent-free conditions by condensation of 5,6-diaminouracil (**3a,b**) with various aromatic aldehydes and characterised using spectroscopic methods and elemental analyses. These synthesised compounds were formulated in selenium nanoforms (**4NPs–9NPs**), which were confirmed by UV-Spectrophotometer, TEM technique, and particle size distribution. All the normal-sized compounds and Se-nanoforms were tested *in vitro* against MCF-7, HepG-2, and A569 cell lines to determine their anti-proliferative activities. Interestingly, all selenium nano-sized forms had a greater inhibitory effect than normal-sized compounds, much outperforming the activity of clinically used standard 5-FU. Compound **4** demonstrated effective anti-proliferative activity against MCF-7 (IC_50_ 3.14 ± 0.04 µM), HepG-2 (IC_50_ 1.07 ± 0.03 µM), and A549 (IC_50_ 1.53 ± 0.01 µM), while its selenium nanoform **4NPs** showed an excellent inhibitory effect, with efficacy increased by 96.52%, 96.45%, and 93.86%, respectively, against the three cell lines when compared to **4**. Additionally, compound **4** showed a pronounced selectivity comparable to 5-fluorouracil against Vero cell lines, whereas **4NPs** demonstrated a five-fold increase in selectivity compared to **4** and a nine-fold increase over 5-FU. Furthermore, **4** exhibited IC_50_ values of 2.18 ± 0.06 µM and 1.92 ± 0.12 µM against CDK1 and tubulin polymerase at the colchicine binding site (CBS), respectively. While **4NPs** strongly inhibited both CDK1 with an IC_50_ of 0.47 ± 0.3 µM, comparable to roscovitine (IC_50_ 0.27 ± 0.03 µM), and tubulin polymerase CBS with an IC_50_ of 0.61 ± 0.04 µM, comparable to combretastatin-A4 (IC_50_ 0.25 ± 0.01 µM). Moreover, compound **4** stopped the cell cycle at G0/G1 phase and forced the cells towards apoptosis, whereas its nanoform **4NPs** caused a significant rise in the proportion of cells arrested at G0/G1 and its apoptotic-inducing potency, indicating an improvement in the test compound’s efficiency. In the molecular docking simulation, **4** and **4NPs** were able to bind to CDK1 and CBS binding sites in inhibitory modes. These findings imply that pyrimidine Schiff base in selenium nanoform **4NPs** is a promising scaffold with a possible anti-proliferative candidate for further research.

## Materials and methods

### Chemistry

Melting points (°C) were measured using the Stuart melting point apparatus (SMP 30) and are uncorrected. Pre-coated (0.25 mm) silica gel plates (Merck 60 F_254_, Germany) were used to monitor reactions, and spots were visualised using a UV lamp (254 nm). Ethyl acetate: toluene (1:1) and chloroform: methanol (9:1) were used as elution systems. NMR spectra were recorded in (DMSO) at ^1^H NMR (400 MHz) and ^13^C NMR (100 MHz) on a Bruker NMR spectrometer (*δ* ppm), Zagazig university using TMS as an internal standard. Mass spectra were done on the direct inlet part of the mass analyser in a Thermo Scientific GCMS model ISQ at Al-Azhar University’s Regional Centre for Mycology and Biotechnology (RCMB), Egypt. The UV-Vis (Shimadzu spectrophotometer) was used to monitor the selenium nanoparticles formation. The UV-Vis spectra were taken between 400 and 700 nm. High-Resolution Transmission Electron Microscopy (HRTEM) JEOL (JEM-2100 TEM) was used to obtain the shape and size of the SeNPs. A drop of colloidal solution was placed on a 400 mesh Carbon-coated Copper grid and evaporated in the air at room temperature to prepare samples for TEM measurements. The average diameter, size distribution, and zeta potential of samples were measured using a particle size analyser (Nano-ZS, Malvern Instruments Ltd., UK). For measuring Size distribution and zeta potential, the sample was sonicated for 30–60 min just before assessment.

#### Materials

The starting compounds, 6-aminouracil **1a,b**, nitroso derivatives **2a,b**, 5,6-diaminouracil derivatives **3a,b** were prepared according to the reported method^41–46^. Aldrich Chemicals Co., USA, as well as commercial sources, provided all of the chemicals and reagents used.

#### General method for the preparation of (E)-6-amino-1-alkyl-5-(arylideneamino)pyrimidine-2,4(1H,3H)-diones (4–9)

A mixture of 5,6-diaminouracils **3a,b** (1.75 mmol) and different appropriate aromatic aldehydes (1.75 mmol) was heated under fusion in the presence of glacial acetic acid (0.7 ml) for 20 min. The formed residue was collected by methanol, filtered, washed with methanol, recrystallized from MeOH-DMF (4:1), and then dried in the oven to afford the desired Schiff′s bases **4–9** in excellent yields.

##### (E)-6-amino-5-((4-(dimethylamino)benzylidene)amino)-1-methylpyrimidine-2,4(1H,3H)-dione (4)

Yellow solid, Yield: 88%; m.p.= 294–296 °C; ^1^H NMR (400 MHz, DMSO) *δ* 10.62 (s, 1H, NH uracil), 9.54 (s, 1H, CH arylidene), 7.68 (d, *J* = 8.8 Hz, 2H, Ar–H), 7.12 (s, 2H, NH_2_), 6.71 (d, *J* = 8.8 Hz, 2H, Ar–H), 3.31 (s, 3H, CH_3_), 2.96 (s, 6H, 2CH_3_). ^13^C NMR (100 MHz, DMSO) *δ* 157.98, 153.95, 151.08, 150.61, 149.25, 128.49, 126.70, 111.70, 99.27, 39.89, 29.27 ppm. MS: *m/z* (rel. int. %) = 287 (M‏^+^, 11), 281 (59), 262 (51), 234 (53), 228 (42), 215 ,(61) 209 (66), 201 (52), 197 (75), 189 (73), 169 (100), 162 (61), 125 (80), 104 (78). Anal. Calcd (%) for C_14_H_17_N_5_O_2_ (287.32): C, 58.52; H, 5.96; N, 24.38; Found: C, 58.69; H, 6.12 N, 24.17.

##### (E)-6-amino-5-((4-hydroxybenzylidene)amino)-1-methylpyrimidine-2,4(1H,3H)-dione (5)

Yellow solid, Yield: 84%; m.p.= 259–260 °C; ^1^H NMR (400 MHz, DMSO) *δ* 10.65 (s, 1H, NH uracil), 9.57 (s, 1H, CH arylidene), 9.30 (s, 1H, OH), 7.70 (d, *J* = 8.6 Hz, 2H, Ar–H), 7.18 (s, 2H, NH_2_), 6.78 (d, *J* = 8.6 Hz, 2H, Ar–H), 3.32 (s, 3H, CH_3_).^13^C NMR (100 MHz, DMSO) *δ* 158.76, 158.01, 154.32, 150.07, 149.30, 130.04, 128.88, 115.36, 99.08, 29.34 ppm. MS: *m/z* (rel. int. %) = 260 (M‏^+^, 38), 248 (60), 245 (60), 190 (48), 184 (96), 180 (46), 173 (56), 159 (72), 154 (80), 138 (50), 82 (48), 70 (100). Anal. Calcd (%) for C_12_H_12_N_4_O_3_ (260.25): C, 55.38; H, 4.65; N, 21.53; Found: C, 55.67; H, 4.82; N, 21.74.

##### (E)-6-amino-1-ethyl-5-((4-hydroxybenzylidene)amino)pyrimidine-2,4(1H,3H)-dione (6)

Yellow solid, Yield: 85%; m.p.= 274–275 °C; ^1^H NMR (400 MHz, DMSO) *δ* 10.64 (s, 1H, NH uracil), 9.71 (s, 1H, CH arylidene), 9.57 (s, 1H, OH), 7.70 (d, *J* = 8.4 Hz, 2H, Ar–H), 7.23 (s, 2H, NH_2_), 6.79 (d, *J* = 8.4 Hz, 2H, Ar–H), 3.94 (q, *J* = 6.8 Hz, 2H, CH_2_), 1.15 (t, *J* = 6.8 Hz, 3H, CH_3_) ppm.^13^C NMR (100 MHz, DMSO) *δ* 159.12, 158.41, 153.79, 151.01, 149.45, 130.31, 129.31, 115.86, 99.43, 37.58, 13.36 ppm. MS: *m/z* (rel. int. %) = 274 (M‏^+^, 31), 258 (100), 251 (97), 245 (50), 224 (58), 201 (46), 152 (78), 99 (52). Anal. Calcd (%) for C_13_H_14_N_4_O_3_ (274.28): C, 56.93; H, 5.15; N, 20.43; Found: C, 57.04; H, 5.37; N, 20.69.

##### (E)-6-amino-1-ethyl-5-((4-nitrobenzylidene)amino)pyrimidine-2,4(1H,3H)-dione (7)

Orange solid, Yield: 94%; m.p.>300 °C; ^1^H NMR (400 MHz, DMSO) *δ* 10.82 (s, 1H, NH uracil), 9.77 (s, 1H, CH arylidene), 8.20 (d, *J* = 9.0 Hz, 2H, Ar–H), 8.15 (d, *J* = 9.0 Hz, 2H, Ar–H), 7.62 (s, 2H, NH_2_), 3.97 (q, *J* = 7.0 Hz, 2H, CH_2_), 1.17 (t, *J* = 7.0 Hz, 3H, CH_3_) ppm.^13^C NMR (100 MHz, DMSO) *δ* 157.94, 154.66, 148.91, 146.97, 145.92, 145.01, 127.87, 123.73, 99.58, 37.24, 13.00. ppm. MS: *m/z* (rel. int. %) = 303 (M‏^+^, 49), 289 (76), 282 (58), 267 (48), 249 (50), 244 (55), 225 (74), 213 (62), 197 (40), 189 (78), 177 (76), 172 (100), 147 (68), 113 (56), 107 (70). Anal. Calcd (%) for C_13_H_13_N_5_O_4_ (303.28): C, 51.49; H, 4.32; N, 23.09; Found: C, 51.72; H, 4.50; N, 23.21.

##### (E)-6-amino-1-ethyl-5-((2-nitrobenzylidene)amino)pyrimidine-2,4(1H,3H)-dione (8)

Orange solid, Yield: 92%; m.p.>300 °C; ^1^H NMR (400 MHz, DMSO) *δ* 10.75 (s, 1H, NH uracil), 9.89 (s, 1H, CH arylidene), 8.40 (d, *J* = 7.6 Hz, 1H, Ar–H), 7.87 (d, *J* = 7.6 Hz, 1H, Ar–H), 7.69 (t, *J* = 7.6 Hz, 1H, Ar–H), 7.59–7.51 (m, 1H, Ar–H), 7.45 (s, 2H, NH_2_), 3.95 (q, *J* = 6.8 Hz, 2H, CH_2_), 1.16 (t, *J* = 6.8 Hz, 3H, CH_3_) ppm.^13^C NMR (100 MHz, DMSO) *δ* 157.89, 154.57, 148.88, 148.56, 142.58, 132.49, 131.59, 129.25, 128.58, 123.69, 99.44, 37.21, 12.96 ppm. MS: *m/z* (rel. int. %) = 303 (M‏^+^, 32), 216 (30), 169 (47), 118 (38), 97 (44), 80 (45), 80 (45), 77 (59), 57 (100). Anal. Calcd (%) for C_13_H_13_N_5_O_4_ (303.28): C, 51.49; H, 4.32; N, 23.09; Found: C, 51.74; H, 4.51; N, 23.28.

##### (E)-6-amino-5-((4-bromobenzylidene)amino)-1-ethylpyrimidine-2,4(1H,3H)-dione (9)

Yield: 92%; m.p. >300 °C; ^1^H NMR (400 MHz, DMSO) *δ* 10.53 (s, 1H, NH), 9.64 (s, 1H, CH arylidene), 7.85 (d, *J* = 8.5 Hz, 2H, Ar–H), 7.56 (d, *J* = 8.5 Hz, 2H, Ar–H), 7.42 (s, 2H, NH_2_), 3.95 (q, *J* = 6.9 Hz, 2H, CH_2_), 1.15 (t, *J* = 6.9 Hz, 3H, CH_3_). ^13^C NMR (100 MHz, DMSO) *δ* 157.96, 153.89, 150.50, 148.82, 137.92, 131.99, 131.34, 129.07, 128.25, 122.06, 98.88, 37.05, 13.02. ppm. MS: *m/z* (rel. int. %) = 339 (M + 2, 43), 337 (M^+^, 37), 313 (35), 247 (39), 238 (65), 192 (66), 145 (42), 131 (46), 123 (57), 93 (100), 77 (54); Anal. Calcd (%) for C_13_H_13_BrN_4_O_2_ (337.18): C, 46.31; H, 3.89; 70; N, 16.62. Found: C, 46.49; H, 4.07; 70; N, 16.87.

#### Synthesis of in-situ selenium nanoparticles (SeNPs) 4NPs-9NPs using synthesised pyrimidine Schiff bases 4–9

In a conical flask, selenious acid (H_2_SeO_3_, 0.013 gm, 0.01 mmol) was dissolved in deionised water (80 ml). After heating the selenious acid solution to 60 °C, compounds **4–9** (0.01 mmol) in DMSO (10 ml) were added. 200 µL of 40 mM ascorbic acid was added as a catalyst after 1 h of continuous stirring at 60 °C, and the ruby red SeNPs were suspended. UV-Spectrophotometer, particle size distribution, and TEM were used to confirm and characterise the formation of selenium nanoparticles.

### Biological evaluation

#### In vitro cytotoxicity assay

The cytotoxic activity of all synthesised compounds, both normal and nano-sized forms, was evaluated against three cancer cell lines, breast cancer (MCF-7), hepatocellular carcinoma (HepG-2), and non-small cell lung cancer (A549), while the normal Vero cell line was performed for the promising compounds **4, 6** and their nano-sized **4NPs, 6NPs,** using the MTT assay according to the literature^64^. The cell lines were obtained from ATCC (American Type Culture Collection). 5-Fluorouracil was used as a control. It is worth noting that freshly prepared nanoparticle-based compounds were used in each of the conducted experiments. The protocol of the MTT assay was illustrated in detail in the Supplementary File.

#### In vitro enzymes inhibition assay

##### Estimation of the activity of CDK1

The most active compound **4** and its selenium nanoform **4NPs** were evaluated for their CDK1 inhibition activity. Anti-CDK1/cyclin B1 assay was performed *in vitro* according to the manufacturer’s instructions^4^ as described in the Supplementary File.

##### Determination of colchicine–tubulin binding capacity

Additionally, the colchicine–tubulin binding site inhibition activity of the test compounds **4** and **4NPs** was investigated using the reported method, as described in the Supplementary File^65^.

#### Flow cytometric analysis of cell-cycle distribution

Cell cycle flow cytometry was performed using propidium iodide (PI) staining (ab139418_Propidium Iodide Flow Cytometry Kit/BD)^66^. IC_50_ of both **4** and **4NPs** was introduced to HepG-2 cells and then incubated for 48 h. The cells were then fixed in 70% ethanol for 12 h at 4 °C. After that, the cells were washed with cold PBS, incubated with 100 μL RNase A at 37 °C for 30 min, and stained with 400 μL PI for another 30 min at room temperature in the dark. Flowing software was used to analyse the data after measuring the stained cells with an Epics XL-MCL™ Flow Cytometer (Beckman Coulter).

#### Cellular apoptosis analysis

The apoptotic potency of compounds **4** and **4NPs** was investigated using an Annexin-V assay^67^ and biparametric cytofluorimetric analysis. The liver HepG-2 cells have been subjected to treatment with an IC_50_ of both **4** and **4NPs** for 48 h before being trypsinized, centrifuged, and rinsed twice with PBS before being suspended in 500 μL of binding buffer and double stained for 15 min with 5 μL Annexin V-FITC and 5 μL PI in the dark at room temperature. Stained cells have been counted and analysed with an Epics XL-MCL™ Flow Cytometer and Flowing software.

### Molecular docking simulation

Molecular docking has been performed using the Molecular Operating Environment (MOE) software, according to the literature^68^. First, the MOE builder was used to build ligands in 3D conformation *via* 3D protonation and assigning partial charges, followed by energy minimisation using the MMFF94x force field. The biological targets needed for the docking study were obtained from the RCSB-Protein Data Bank (PDB ID: 6GU6)^58^; cyclin-dependent kinase 1 (CDK1) in complex with dinaciclib and combretastatin-A4 at the colchicine binding site of microtubules (PDB ID: 5LYJ)^63^. The proteins were prepared in a four-step preparation protocol, including 3D protonation and auto-correction for atoms types, connections, and assigning charges. Finally, the active sites were defined with the help of MOE Alpha Site Finder. Dummy atoms have been generated for the alpha spheres and used as polar-hydrophobic descriptors of the active sites of both 6GU6 and 5LYJ. The MOE-induced fit docking protocol was adopted. For validation, re-docking of the native ligands against both the 6GU6 and 5LYJ active sites was performed, and the cut-off of root-mean-square deviation (RMSD) values were < 2.0 Å. The interaction of ligands at the active sites was assessed *via* bond rotation inside the active site using the triangle matcher approach and London dG scoring function as first scoring function. Five poses out of 30 were retained after using GBVI/WSA dG force field-based scoring function as a second scoring function. Poses that had a lower RMSD and a higher S-value were recorded.

## Consent form

Authors accept the final version submitted to the journal.

## Supplementary Material

Supplemental Material
