# Supplementary material for: Novel pyrimidine Schiff bases and their selenium-containing nanoparticles as dual inhibitors of CDK1 and tubulin polymerase: design, synthesis, anti-proliferative evaluation, and molecular modelling
Source: J Enzyme Inhib Med Chem. 2023 Jul 4;38(1):2232125. doi: 10.1080/14756366.2023.2232125 (PMC10324454; doi:10.1080/14756366.2023.2232125)

Supporting information

Novel pyrimidine Schiff bases and their selenium-containing nanoparticles as dual inhibitors of CDK1 and tubulin polymerase: design, synthesis, anti-proliferative evaluation and molecular modeling

Samar El-Kalyoubi[1*], Samiha A. El-Sebaey[2*], Ahmed A. El-Sayed[3], Moustafa S. Abdelhamid[4], Fatimah Agili[5], Sherin M. Elfeky[6]

[1]Department of Pharmaceutical Organic Chemistry, Faculty of Pharmacy, Port Said University, 42511 Port Said, Egypt.
[2]Department of Pharmaceutical Organic Chemistry, Faculty of Pharmacy (Girls), Al-Azhar University, Youssef Abbas Street, Nasr City, Cairo, Egypt
[3]Photochemistry Department, Chemical Industries Research Institute, National Research Centre, 33 El-Bohouth St., Dokki - 12622, Giza, Egypt
[4]Department of Biochemistry, Faculty of Science, Zagazig University, Zagazig 44519, Egypt
[5]Chemistry Department, Faculty of Science (Female Section), Jazan University, Jazan 82621, Saudi Arabia
[6]Department of Pharmaceutical Organic Chemistry, Faculty of Pharmacy, Mansoura University, Mansoura 355516, Egypt.

*Corresponding authors:
1- Samiha A. El-Sebaey; samiha.ali85@azhar.edu.eg; https://orcid.org/0000-0002-9928-9080
2- Samar El-Kalyoubi; S.elkalyoubi@pharm.psu.edu.eg; s.elkalyoubi@hotmail.com; https://orcid.org/0000-0002-4648-5184

<u>Table of Contents</u>

	Figure No.	Page No.
Biological tests	-	S3, S4
^{1}H NMR, ^{13}C NMR and Mass spectra of ***(E)-6-amino-5-((4-(dimethylamino)benzylidene)amino)-1-methylpyrimidine-2,4(1H,3H)-dione (4)***	**Figure S1**	S5
^{1}H NMR, ^{13}C NMR and Mass spectra of ***(E)-6-amino-5-((4-hydroxybenzylidene)amino)-1-methylpyrimidine-2,4(1H,3H)-dione (5)***	**Figure S2**	S6
^{1}H NMR, ^{13}C NMR and Mass spectra of ***(E)-6-amino-1-ethyl-5-((4-hydroxybenzylidene)amino)pyrimidine-2,4(1H,3H)-dione (6)***	**Figure S3**	S7, S8
^{1}H NMR, ^{13}C NMR and Mass spectra of ***(E)-6-amino-1-ethyl-5-((4-nitrobenzylidene)amino)pyrimidine-2,4(1H,3H)-dione (7)***	**Figure S4**	S9, S10
^{1}H NMR, ^{13}C NMR and Mass spectra of ***(E)-6-amino-1-ethyl-5-((2-nitrobenzylidene)amino)pyrimidine-2,4(1H,3H)-dione (8)***	**Figure S5**	S11, S12
^{1}H NMR, ^{13}C NMR and Mass spectra of ***(E)-6-amino-5-((4-bromobenzylidene) amino)-1-ethylpyrimidine-2,4(1H,3H)-dione (9)***	**Figure S6**	S13, S14
Particle size from DLS of SeNPs **4NPs-9NPs**	**Figure S7**	S15
Polydispersity Index (PDI) of SeNPs **4NPs-9NPs**	**Figure S8**	S16
Zeta potential of SeNPs **4NPs-9NPs**	**Figure S9**	S17
Dinaciclib at the active site of CDK1 (PDB ID: 6GU6)	**Figure S10**	S18
Combretastatin-A4 at the colchicine binding site (CBS) of microtubules (PDB ID: 5LYJ)	**Figure S11**	S19
Viability/cytotoxicity Lab Report of (**4-9**), (**4NPs-9NPs**), and 5-FU against MCF-7, HepG-2, A549, and Vero cells	**-**	S20-S56
CDK1 Lab Report	**-**	S57
Colchicine binding assay Lab Report	**-**	S58

1.1. Biological activity

1.1.1. *In vitro* cytotoxicity assay

The cytotoxic activity of all synthesized compounds, both normal and nano-sized forms, was evaluated against three cancer cell lines, breast cancer (MCF-7), hepatocellular carcinoma (HepG-2), and non-small cell lung cancer (A549), while the normal Vero cell line was performed for the promising compounds **4, 6** and their nano-sized **4NPs, 6NPs,** using the MTT assay according to the literature [64]. The cell lines were obtained from ATCC (American Type Culture Collection). 5-Fluorouracil was used as a reference drug. The cytotoxic activities of the examined compounds were quantified by inoculating a 96-well tissue culture plate with 1 X 10^5 cells / mL (100 uL/well) and incubating it at 37°C for 24 hours to develop a complete monolayer sheet. After forming a confluent sheet of cells, the growth medium was decanted from 96-well microtiter plates, and the cell monolayer was washed twice with wash media. Two-fold dilutions of tested samples were made in RPMI medium with 2% serum (maintenance medium), and 0.1 mL of each dilution was tested in different wells, with three wells serving as controls and receiving only maintenance medium. Following that, the plate was incubated at 37°C and examined. Cells were investigated for any physical signs of toxicity, such as partial or complete monolayer loss, rounding, shrinkage, or cell granulation. MTT solution was prepared (5mg/ml in PBS) (BIO BASIC CANADA INC), and 20 uL MTT solution was added to each well. After 5 minutes on a shaking table at 150 rpm to thoroughly mix the MTT into the media, it was incubated (37 ° C, 5% CO_2) for 4 hours to allow the MTT to be metabolized. Formazan (MTT metabolic product) was resuspended in 200 uL DMSO and shaken at 150 rpm for 5 minutes to thoroughly mix the formazan into the solvent. The optical density was measured at 560 nm, and the background was subtracted at 620 nm. Finally, the IC_{50} of the test compound in comparison to the reference was calculated using the GraphPad Prism software.

1.1.2. *In vitro* Enzymes inhibition assay

1.1.2.1. Estimation of the activity of CDK1

The CDK1 kinase was provided by BPS Bioscience Inc. (California, United States). Kinase-Glo® luminescent kinase assay platform was purchased from Promega Corporation (Madison, United States). Anti-CDK1/cyclin B1 assay was carried out *in vitro* according to the manufacturer's instructions [4]. Dimethyl sulfoxide (DMSO) was used in dissolving the tested compounds **4, 4NPs**, and control to prepare 50 µM stock solutions. Double-distilled water and buffer were used to dilute the stock solutions to 40, 30, 20, and 10 µM working concentrations before being stored at −20°C. After mixing buffer, ATP, CDK substrate peptidase, and distilled water, 25 µL of the resulting mixture was added to a 96-well plate. Then, using a buffer, CDK1/cyclin B1 was diluted to 1 ng/µL before adding five microliters of 20 µM test compounds **4** and **4NPs**. Twenty microliters of the CDK1/cyclin B1 solution were incubated at 30°C for 45 minutes with the test compounds **4** and **4NPs**. Finally, after adding 50 L of Kinase-Glo Max reagent to each wall, the 96-well plate was covered with aluminum foil and incubated at room temperature for 15 minutes. A microplate reader was used to measure the luminescence of the tested compounds.

1.1.2.2. Determination of colchicine–tubulin binding capacity

1 μM radiolabeled colchicine (Perkin-Elmer; 1 μCi/μL), 1% DMSO, and various concentrations of the test compounds **4** and **4NPs** were mixed and incubated with 1 mmol/L tubulin (>99% pure; Cytoskeleton, Inc.; 0.2 μg/mL) in 50 μL G-PEM buffer containing 80 mmol/L PIPES (pH 6.8), 1 mmol/L EGTA, 1 mmol/L $MgCl_2$, 1 mmol/L GTP, and 5% glycerol for 60 minutes at 37ºC. The binding solutions were filtered and washed twice through a stack of 2DEAE-cellulose filters. The radioactivity in the filtrates was determined by liquid scintillation spectrometry (Perkin-Elmer Wallac) [65]. Nonlinear regression was used to analyze data using GraphPad Prism.

Figure S1: ^{1}H NMR, ^{13}C NMR and Mass spectra of ***(E)-6-amino-5-((4-(dimethylamino) benzylidene)amino)-1-methylpyrimidine-2,4(1H,3H)-dione (4)***

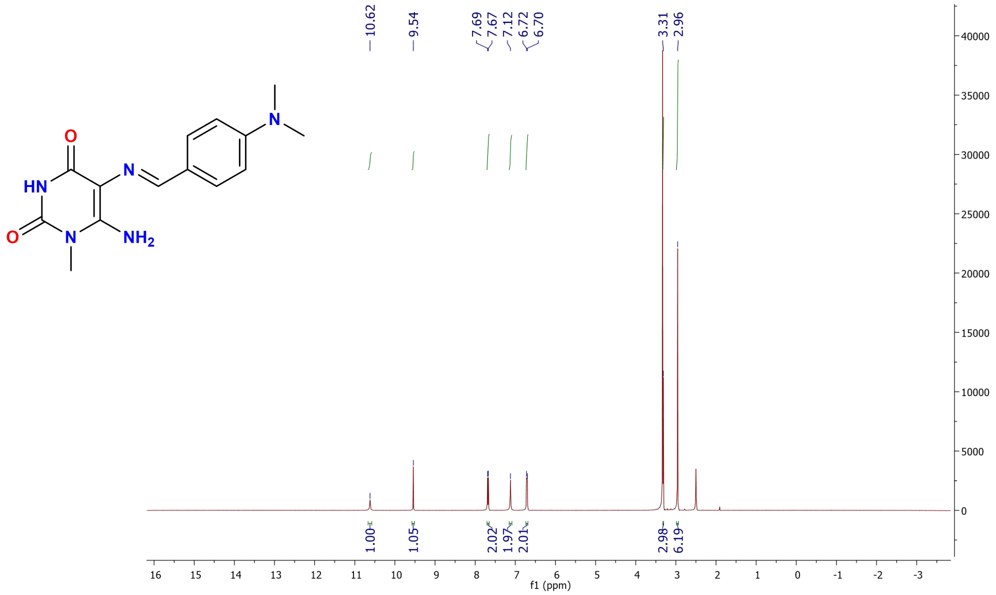

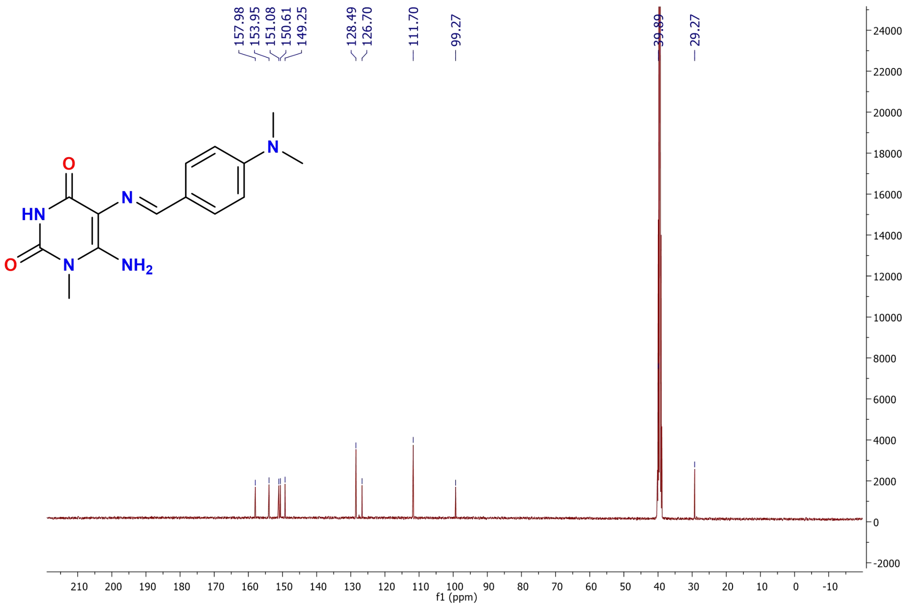

Figure S2: ^{1}H NMR, ^{13}C NMR and Mass spectra of ***(E)-6-amino-5-((4-hydroxybenzylidene)amino)-1-methylpyrimidine-2,4(1H,3H)-dione (5)***

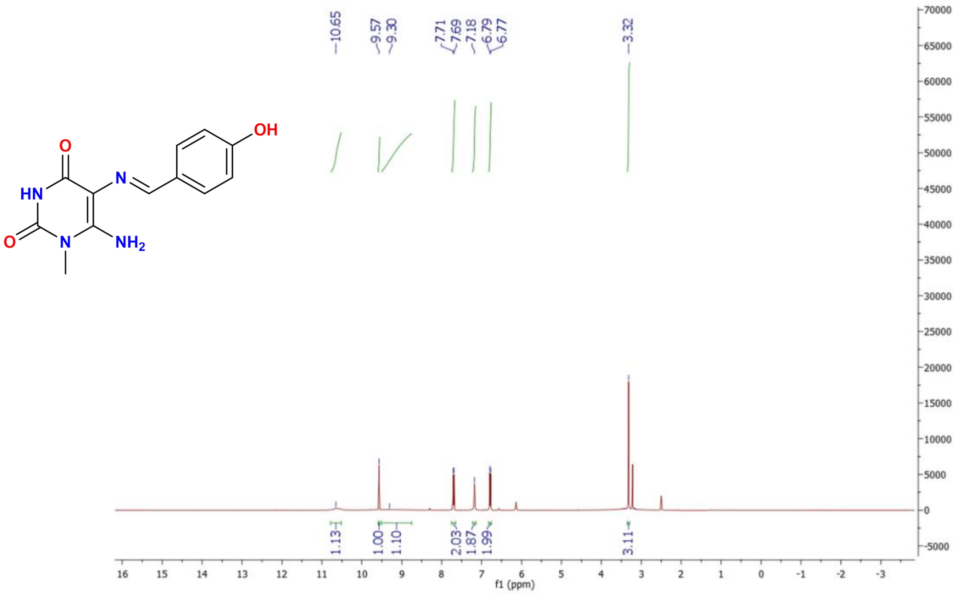

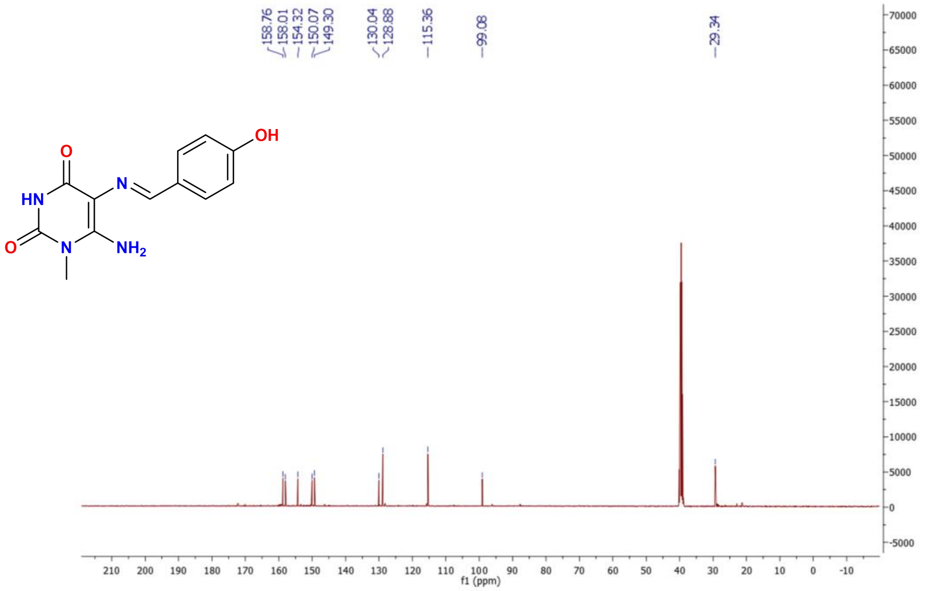

Figure S3: ^{1}H NMR, ^{13}C NMR and Mass spectra of ***(E)-6-amino-1-ethyl-5-((4-hydroxybenzylidene)amino)pyrimidine-2,4(1H,3H)-dione (6)***

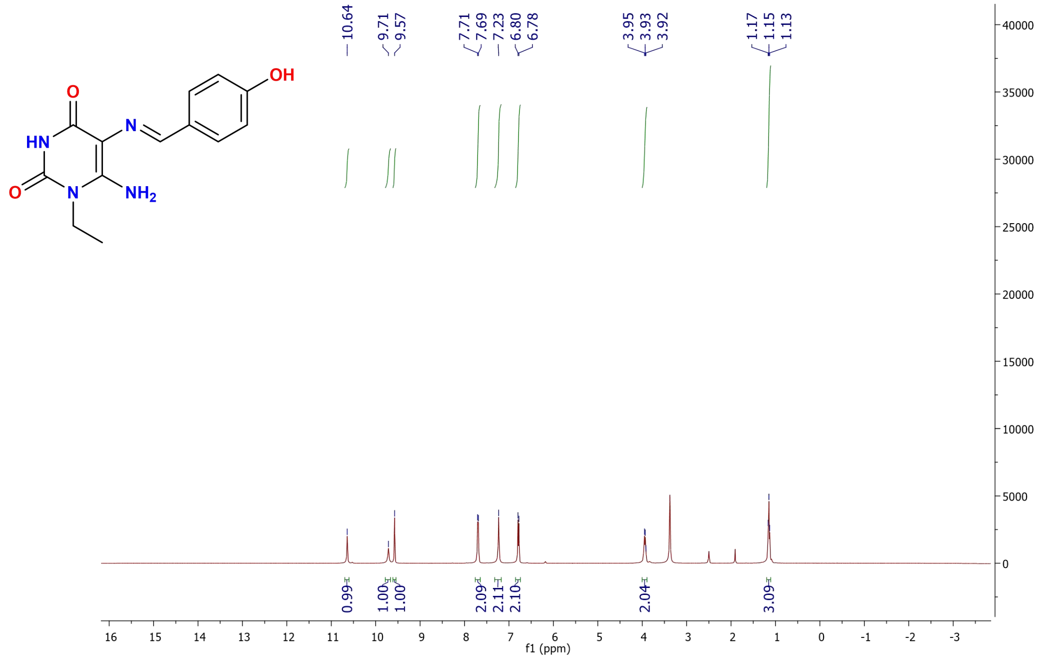

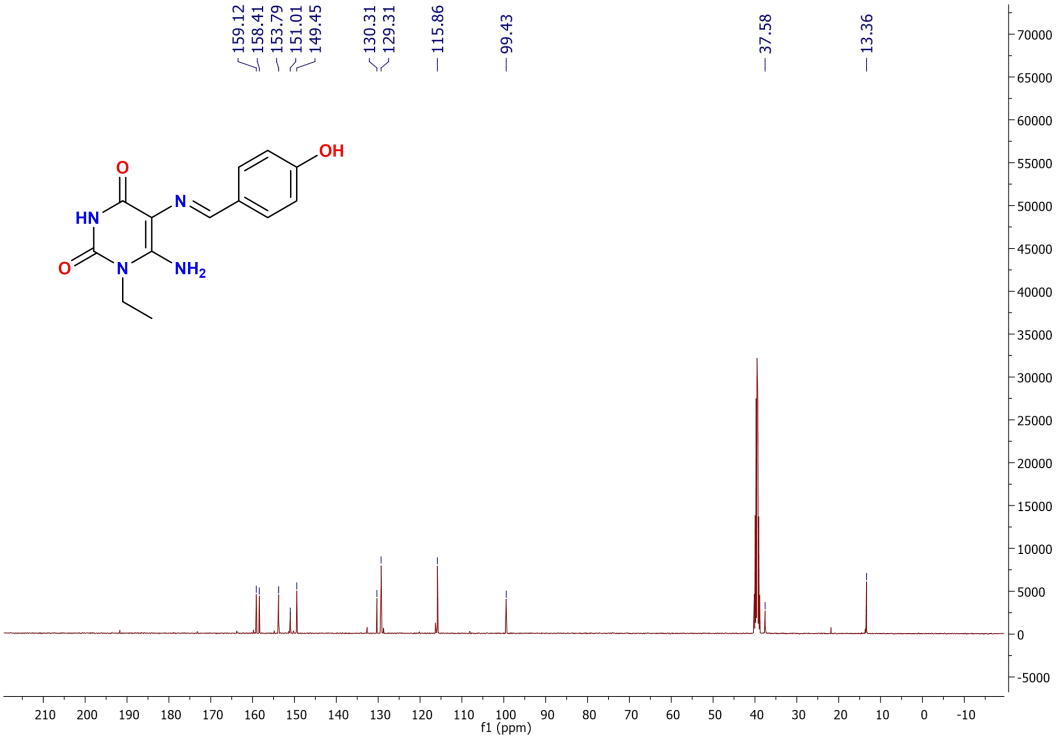

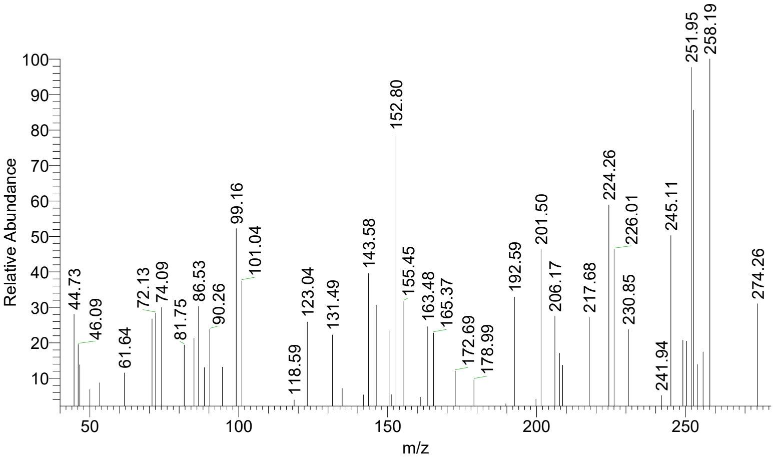

Relative Abundance
m/z
44.73
46.09
61.64
72.13
74.09
81.75
86.53
90.26
99.16
101.04
118.59
123.04
131.49
143.58
152.80
155.45
163.48
165.37
172.69
178.99
192.59
201.50
206.17
217.68
224.26
226.01
230.85
241.94
245.11
251.95
258.19
274.26

Figure S4: ^{1}H NMR, ^{13}C NMR and Mass spectra of ***(E)-6-amino-1-ethyl-5-((4-nitrobenzylidene)amino)pyrimidine-2,4(1H,3H)-dione (7)***

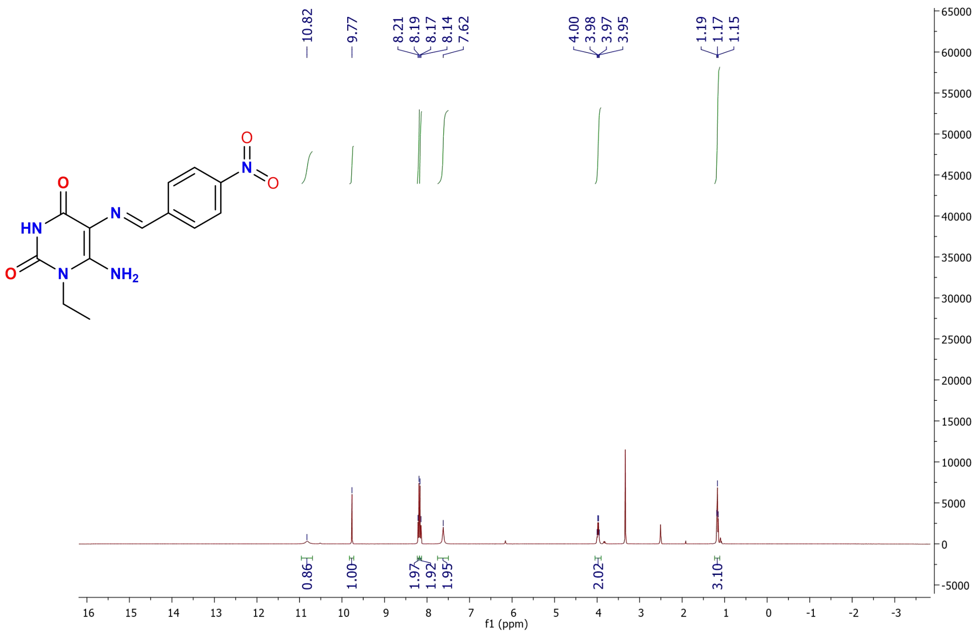

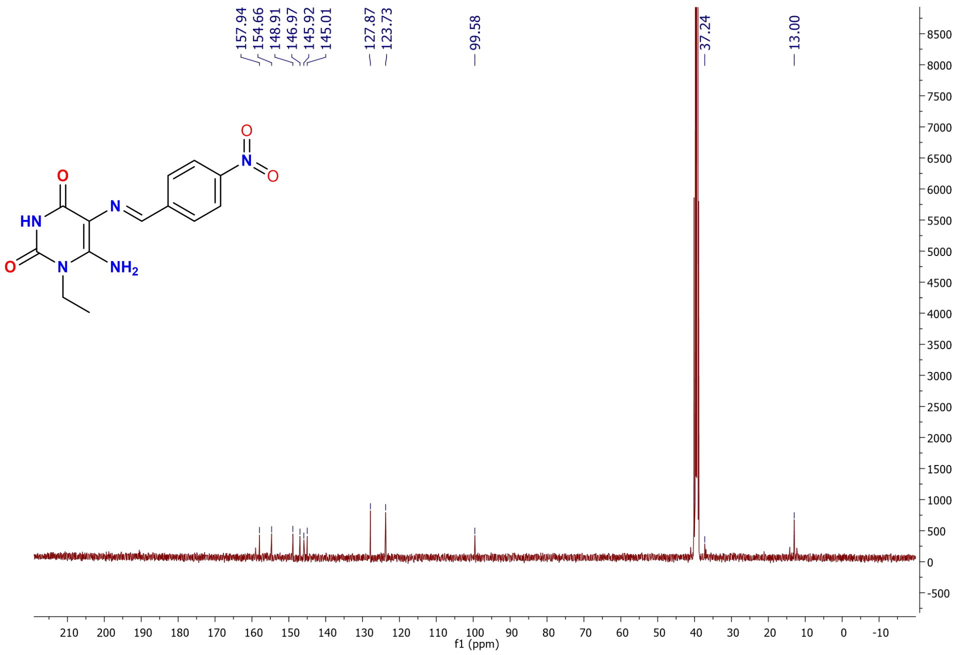

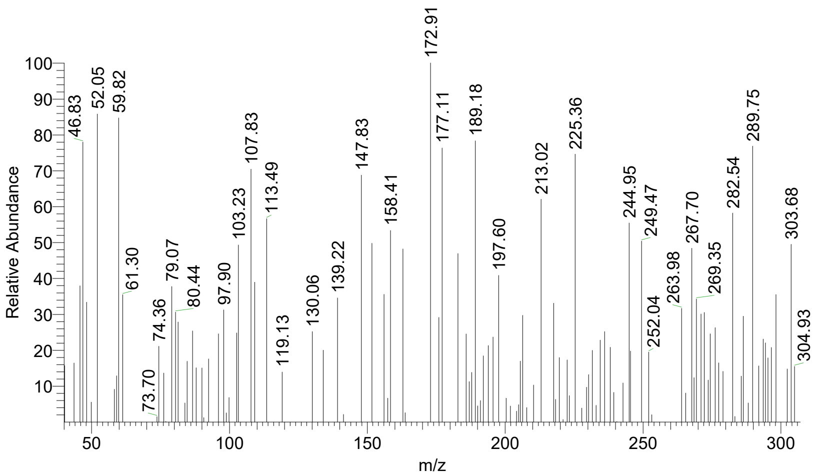

Relative Abundance
m/z

Figure S5: ^{1}H NMR, ^{13}C NMR and Mass spectra of ***(E)-6-amino-1-ethyl-5-((2-nitrobenzylidene)amino)pyrimidine-2,4(1H,3H)-dione (8)***

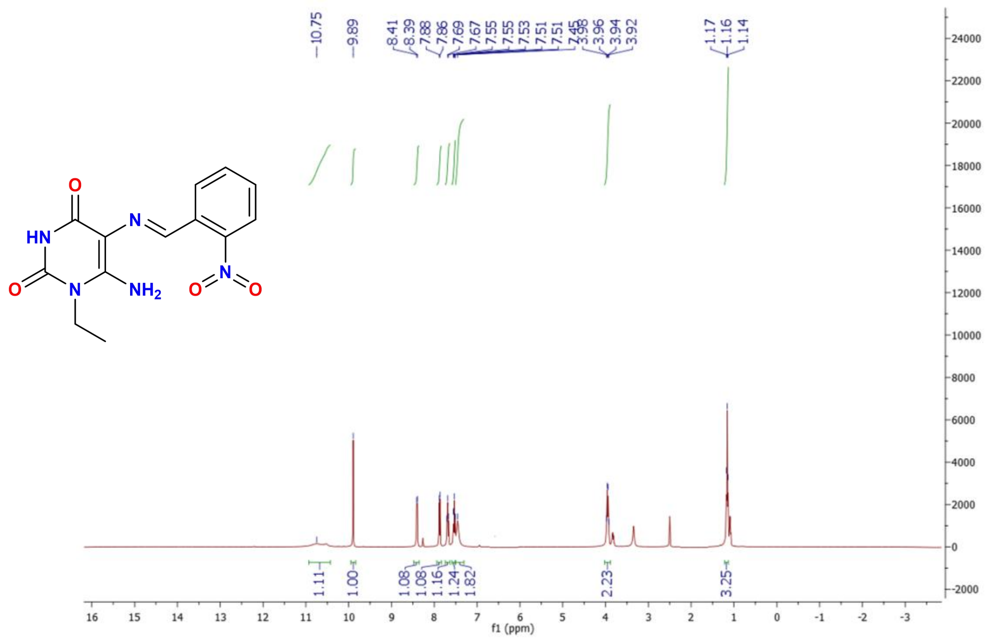

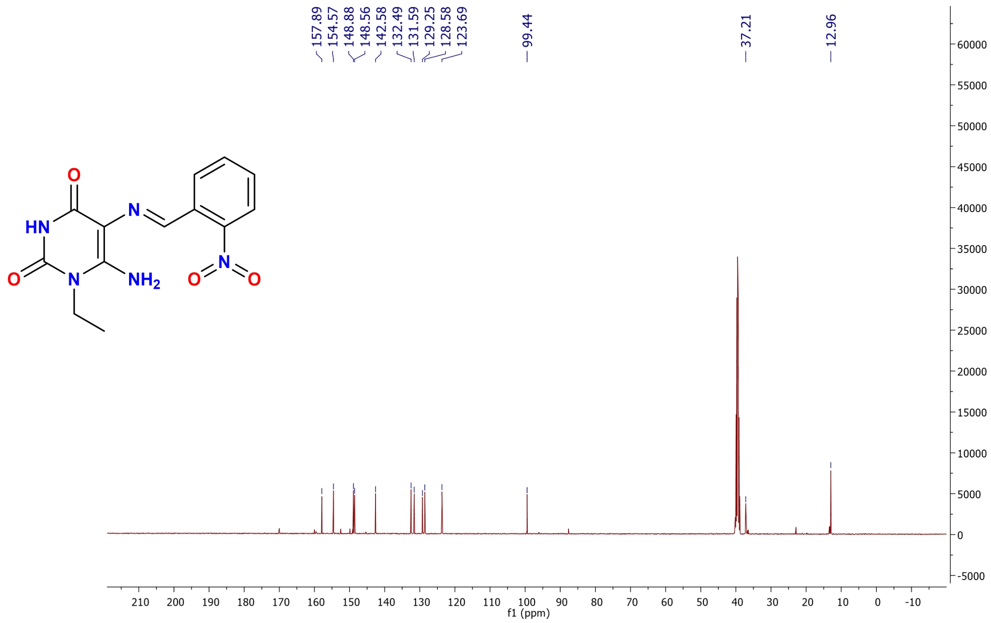

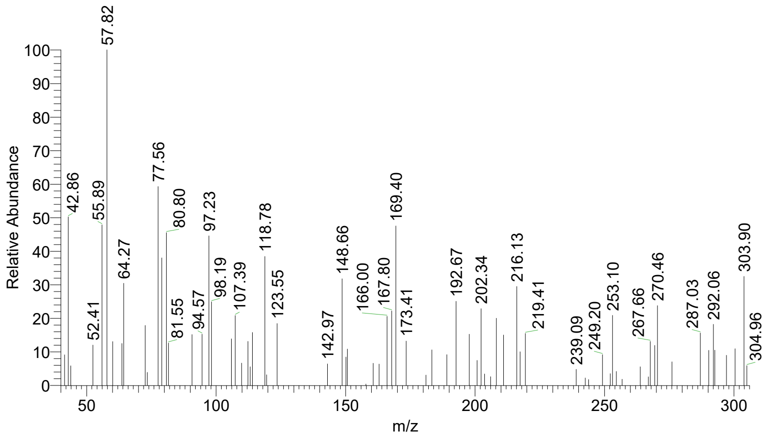
Relative Abundance
m/z
57.82
42.86
55.89
77.56
80.80
97.23
118.78
169.40
148.66
64.27
52.41
81.55
94.57
98.19
107.39
123.55
142.97
166.00
167.80
173.41
192.67
202.34
216.13
219.41
239.09
249.20
253.10
267.66
270.46
287.03
292.06
303.90
304.96

Figure S6: ^{1}H NMR, ^{13}C NMR and Mass spectra of ***(E)-6-amino-5-((4-bromobenzylidene)amino)-1-ethylpyrimidine-2,4(1H,3H)-dione (9)***

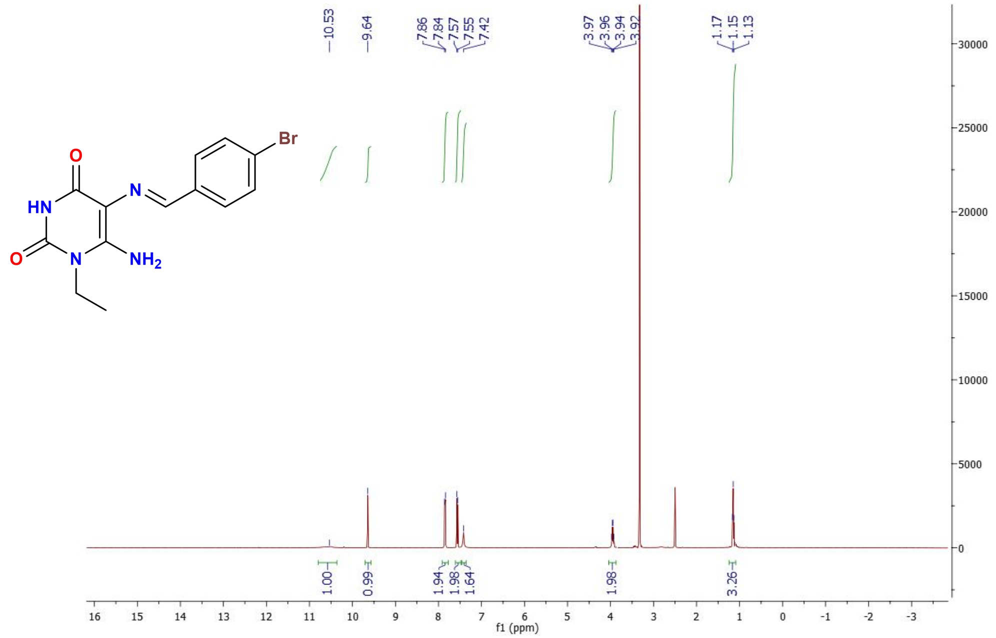

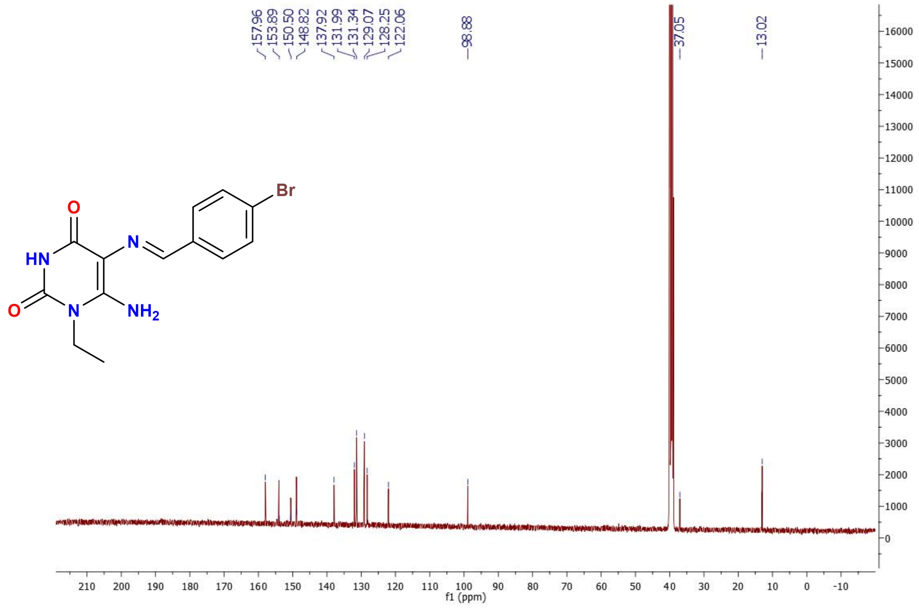

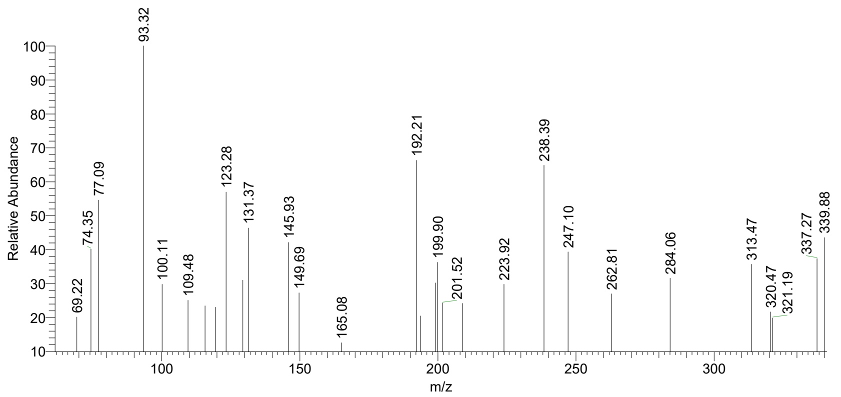

93.32
192.21
238.39
123.28
77.09
131.37
74.35
145.93
339.88
337.27
247.10
199.90
223.92
100.11
284.06
313.47
109.48
149.69
262.81
201.52
320.47
321.19
69.22
165.08
Relative Abundance
m/z

Figure (S7). Particle size from DLS of SeNPs, **a**), **b), c), d), e),** and **f)** Particle size of selenium nanoparticles **4NPs-9NPs,** respectively

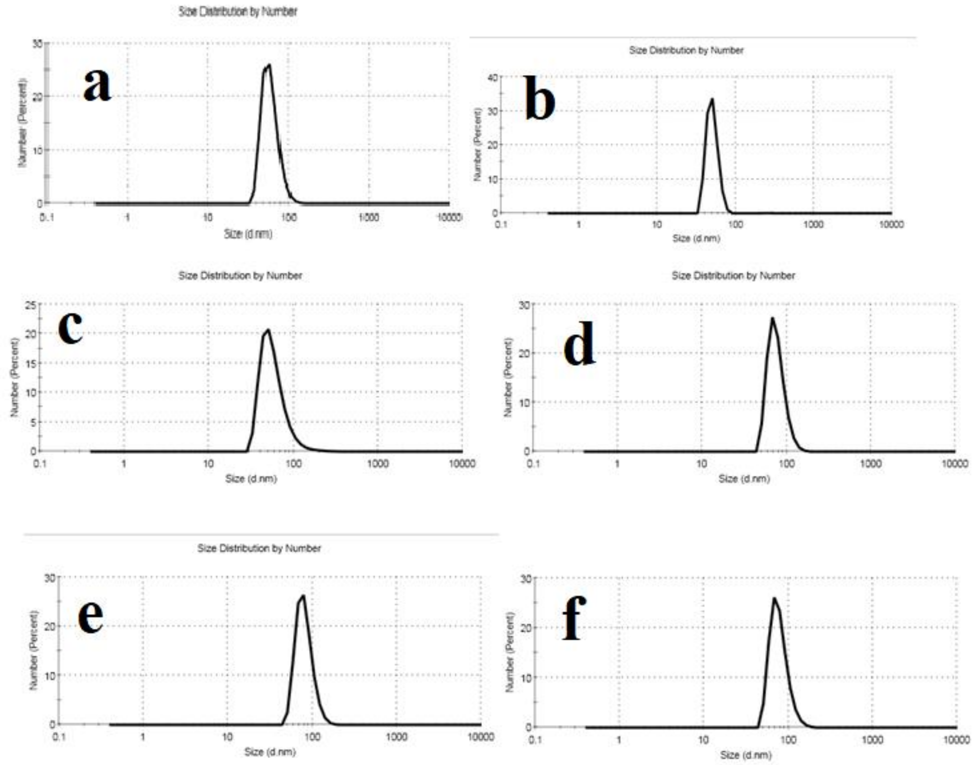

Figure (S8). Polydispersity Index (PDI) of SeNPs, **a), b), c), d), e),** and **f)** PDI of selenium nanoparticles **4NPs-9NPs,** respectively

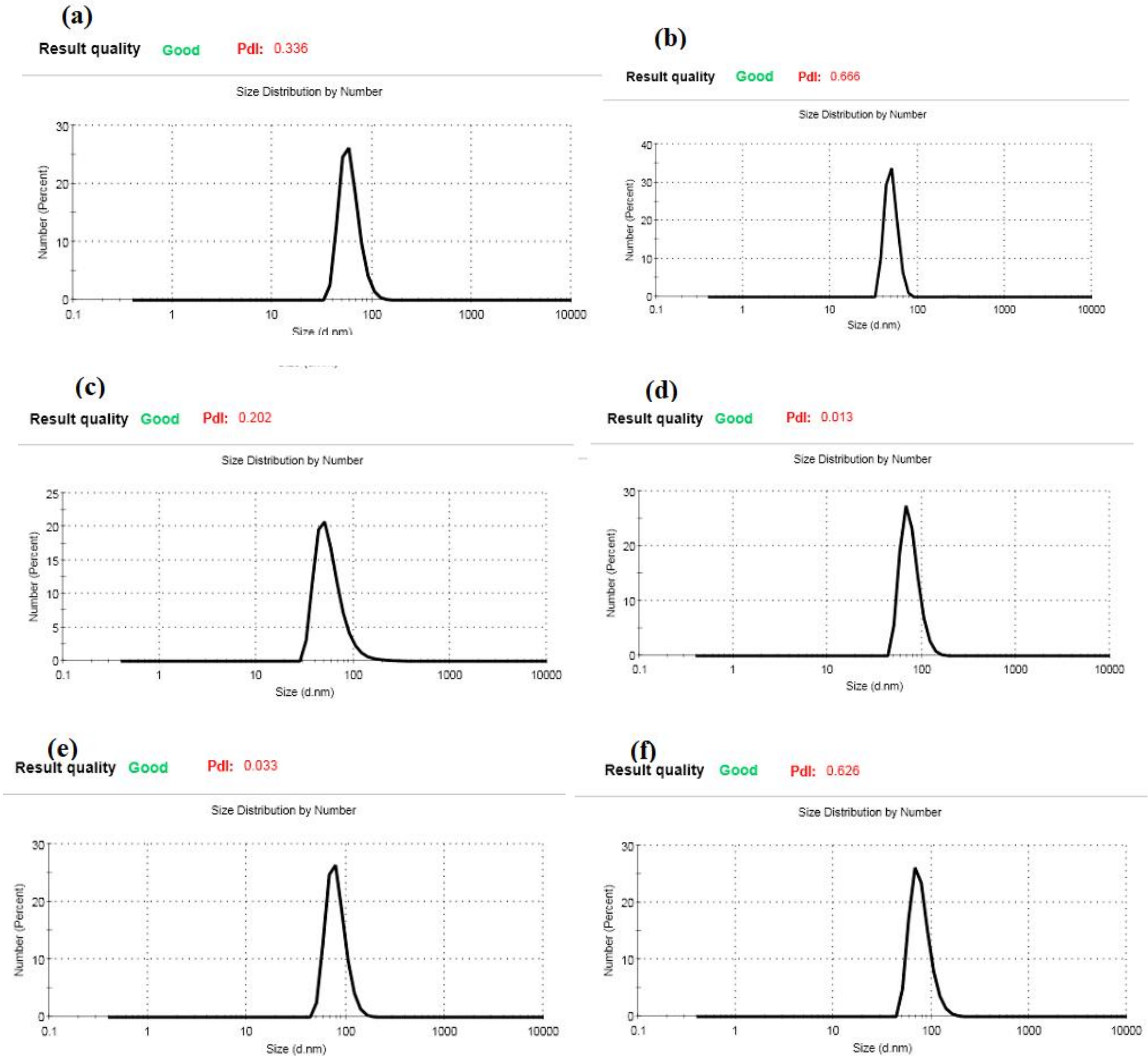

Figure (S9). The Zeta potential of SeNPs, **a), b), c), d), e),** and **f)** Zeta potential of selenium nanoparticles **4NPs-9NPs,** respectively

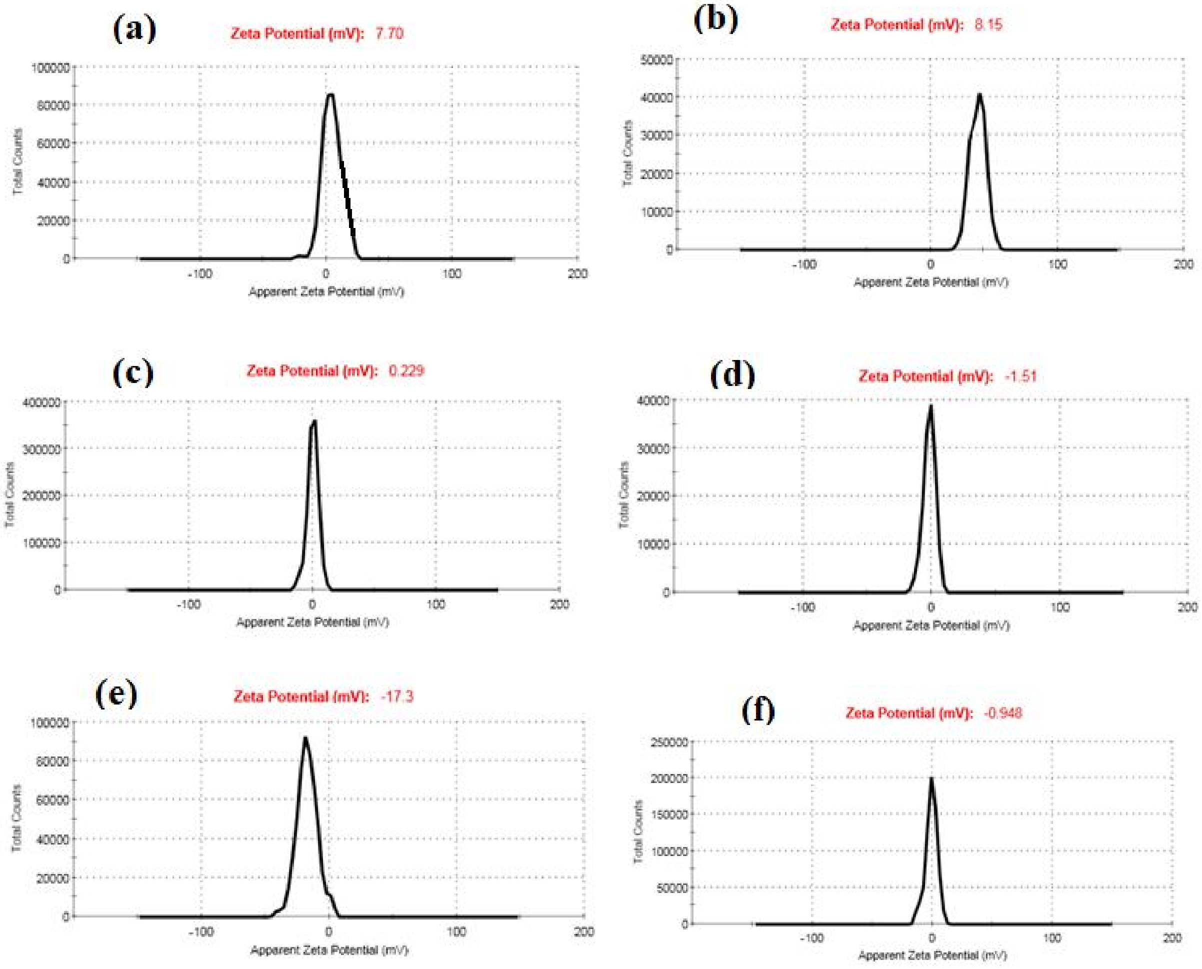

Figure (S10). Dinaciclib at the active site of CDK1 (PDB ID: 6GU6)

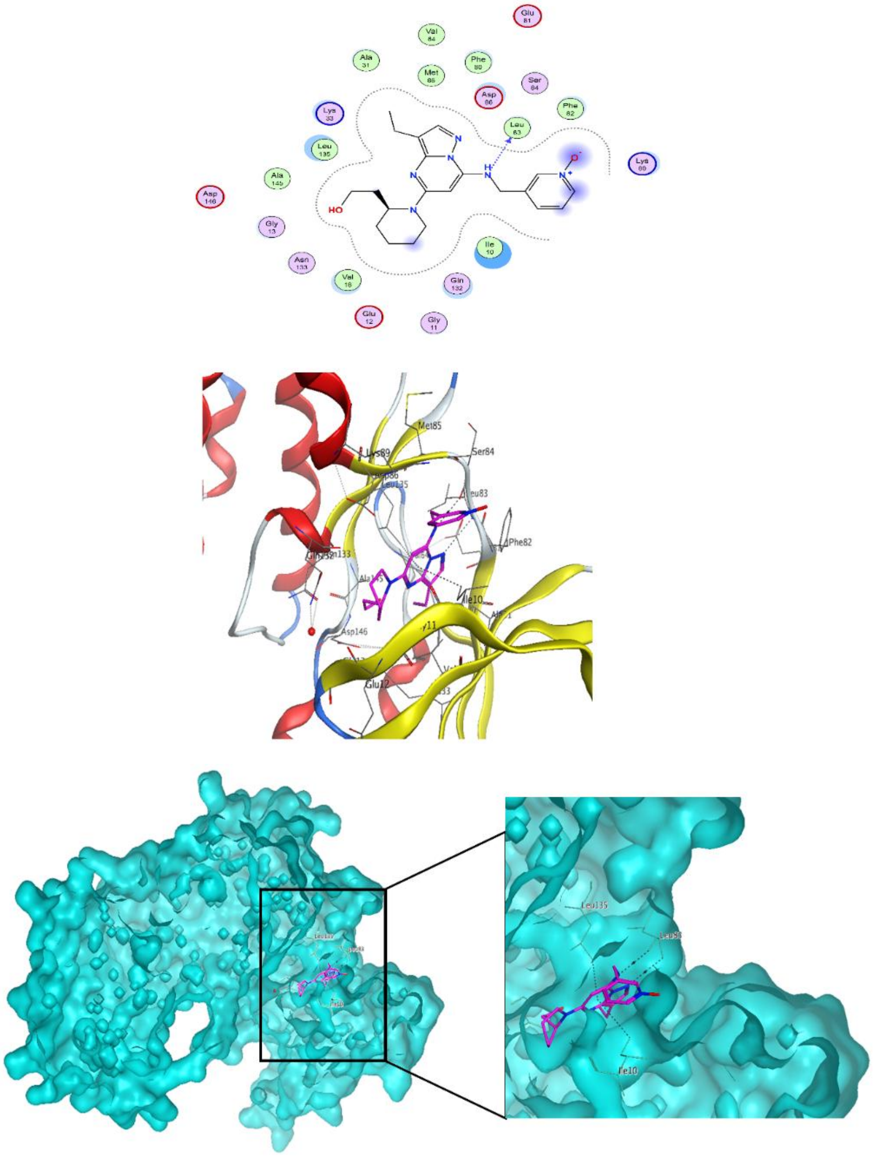

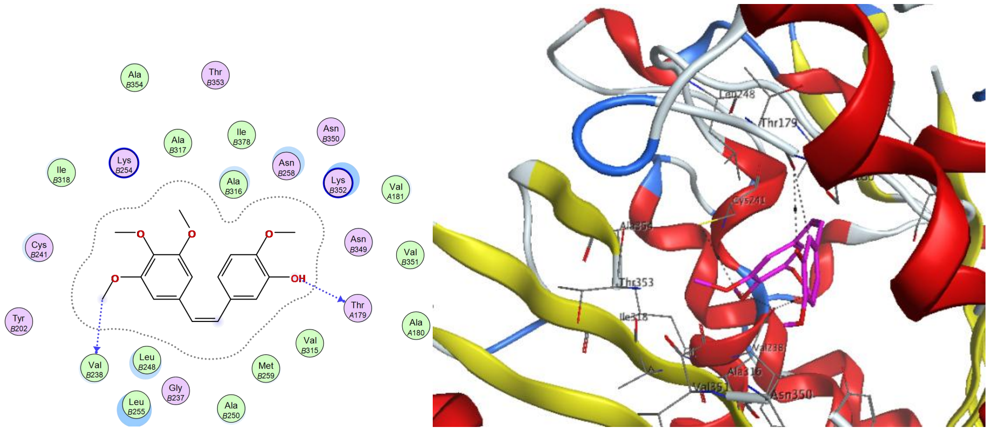

Figure (S11). Combretastatin-A4 at the colchicine binding site (CBS) of microtubules (PDB ID: 5LYJ).

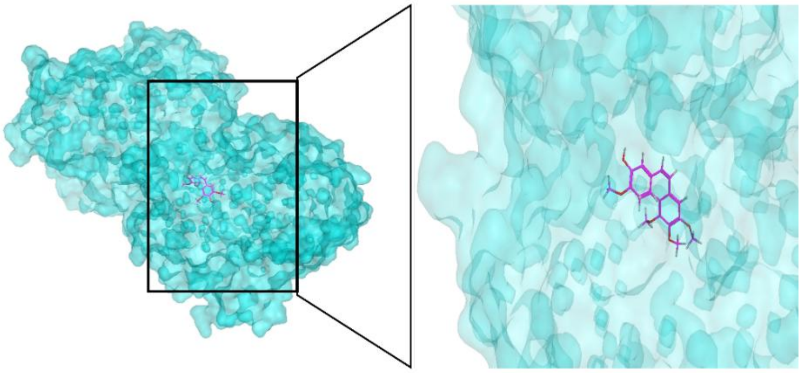

Viability/cytotoxicity Lab Report

Functional assay (MTT)

Test code: T-1-023-20

samples number : 12

experiment design : effect against MCF-7, A549 and HepG2 cells

ID	uM/ml	O.D			Mean O.D	±SE	Viability %	Toxicity %	IC50 ± SD
MCF-7	--------	0.838	0.819	0.824	0.827	0.005686	100	0	uM
Se NPs	1	0.019	0.016	0.017	0.017333	0.000882	2.095929061	97.90407094	0.03 ± 0
	0.5	0.019	0.018	0.019	0.018667	0.000333	2.257154373	97.74284563	
	0.25	0.017	0.02	0.019	0.018667	0.000882	2.257154373	97.74284563	
	0.125	0.019	0.018	0.019	0.018667	0.000333	2.257154373	97.74284563	
	0.062	0.199	0.201	0.184	0.194667	0.005364	23.53889561	76.46110439	
	0.031	0.436	0.47	0.452	0.452667	0.009821	54.73599355	45.26400645	
1 [9NPs]	1	0.019	0.015	0.018	0.017333	0.001202	2.095929061	97.90407094	0.07 ± 0
	0.5	0.019	0.018	0.019	0.018667	0.000333	2.257154373	97.74284563	
	0.25	0.02	0.018	0.021	0.019667	0.000882	2.378073358	97.62192664	
	0.125	0.063	0.066	0.082	0.070333	0.005897	8.504635228	91.49536477	
	0.062	0.315	0.327	0.333	0.325	0.005292	39.29866989	60.70133011	
	0.031	0.725	0.759	0.74	0.741333	0.009838	89.64127368	10.35872632	
2 [7NPs]	1	0.017	0.019	0.018	0.018	0.000577	2.176541717	97.82345828	0.07 ± 0
	0.5	0.018	0.018	0.018	0.018	0	2.176541717	97.82345828	
	0.25	0.019	0.02	0.019	0.019333	0.000333	2.337767029	97.66223297	
	0.125	0.063	0.049	0.047	0.053	0.005033	6.408706167	93.59129383	
	0.062	0.388	0.401	0.395	0.394667	0.003756	47.72269246	52.27730754	
	0.031	0.688	0.659	0.672	0.673	0.008386	81.37847642	18.62152358	
3 [5NPs]	1	0.016	0.019	0.019	0.018	0.001	2.176541717	97.82345828	0.06 ± 0
	0.5	0.018	0.019	0.017	0.018	0.000577	2.176541717	97.82345828	
	0.25	0.017	0.017	0.019	0.017667	0.000667	2.136235389	97.86376461	
	0.125	0.042	0.038	0.044	0.041333	0.001764	4.997984684	95.00201532	
	0.062	0.271	0.269	0.255	0.265	0.005033	32.04353083	67.95646917	
	0.031	0.618	0.658	0.632	0.636	0.011719	76.904474	23.095526	
4 [6NPs]	1	0.018	0.022	0.017	0.019	0.001528	2.297460701	97.7025393	0.04 ± 0
	0.5	0.02	0.019	0.017	0.018667	0.000882	2.257154373	97.74284563	
	0.25	0.018	0.015	0.019	0.017333	0.001202	2.095929061	97.90407094	
	0.125	0.022	0.036	0.031	0.029667	0.004096	3.5872632	96.4127368	
	0.062	0.094	0.153	0.127	0.124667	0.017072	15.07456671	84.92543329	
	0.031	0.462	0.489	0.451	0.467333	0.011289	56.50947199	43.49052801	
	1	0.017	0.018	0.017	0.017333	0.000333	2.095929061	97.90407094	

5 [4NPs]	0.5	0.019	0.017	0.019	0.018333	0.000667	2.216848045	97.78315195	0.04 ± 0
	0.25	0.019	0.02	0.021	0.02	0.000577	2.418379686	97.58162031	
	0.125	0.018	0.022	0.02	0.02	0.001155	2.418379686	97.58162031	
	0.062	0.114	0.162	0.108	0.128	0.017088	15.47762999	84.52237001	
	0.031	0.47	0.441	0.498	0.469667	0.016455	56.79161628	43.20838372	
6 [8NPs]	1	0.019	0.016	0.015	0.016667	0.001202	2.015316405	97.9846836	0.04 ± 0
	0.5	0.018	0.019	0.019	0.018667	0.000333	2.257154373	97.74284563	
	0.25	0.02	0.017	0.022	0.019667	0.001453	2.378073358	97.62192664	
	0.125	0.022	0.026	0.019	0.022333	0.002028	2.700523982	97.29947602	
	0.062	0.152	0.199	0.172	0.174333	0.013618	21.08020959	78.91979041	
	0.031	0.462	0.511	0.496	0.489667	0.014495	59.20999597	40.79000403	

ID	uM/ml	O.D			Mean O.D	±SE	Viability %	Toxicity %	IC50 ± SD
MCF-7	--------	0.838	0.819	0.824	0.827	0.005686	100	0	uM
Old 1 [7]	10	0.033	0.028	0.031	0.030667	0.001453	3.708182185	96.29181782	3.37 ± 0.04
	5	0.177	0.153	0.18	0.17	0.008544	20.55622733	79.44377267	
	2.5	0.463	0.5	0.492	0.485	0.01124	58.64570738	41.35429262	
	1.25	0.833	0.82	0.823	0.825333	0.00393	99.79846836	0.20153164	
	0.625	0.819	0.832	0.824	0.825	0.003786	99.75816203	0.241837969	
	0.312	0.827	0.819	0.817	0.821	0.003055	99.27448609	0.725513906	
Old 2 [8]	10	0.05	0.038	0.044	0.044	0.003464	5.320435308	94.67956469	2.03 ± 0.07
	5	0.092	0.091	0.117	0.1	0.008505	12.09189843	87.90810157	
	2.5	0.359	0.36	0.422	0.380333	0.020835	45.98952035	54.01047965	
	1.25	0.518	0.473	0.499	0.496667	0.013043	60.05642886	39.94357114	
	0.625	0.83	0.831	0.819	0.826667	0.003844	99.95969367	0.040306328	
	0.312	0.827	0.829	0.821	0.825667	0.002404	99.83877469	0.161225312	
Old 3 [4]	10	0.019	0.022	0.018	0.019667	0.001202	2.378073358	97.62192664	3.17 ± 0.04
	5	0.092	0.115	0.142	0.116333	0.014449	14.0669085	85.9330915	
	2.5	0.538	0.488	0.474	0.5	0.019425	60.45949214	39.54050786	
	1.25	0.749	0.733	0.772	0.751333	0.011319	90.85046352	9.149536477	
	0.625	0.811	0.794	0.788	0.797667	0.006888	96.45304313	3.546956872	
	0.312	0.829	0.822	0.822	0.824333	0.002333	99.67754938	0.322450625	
Old 4 [5]	10	0.018	0.02	0.02	0.019333	0.000667	2.337767029	97.66223297	3.59 ± 0.08
	5	0.173	0.214	0.199	0.195333	0.011977	23.61950826	76.38049174	
	2.5	0.572	0.555	0.569	0.565333	0.005239	68.35953245	31.64046755	
	1.25	0.791	0.835	0.802	0.809333	0.01322	97.86376461	2.136235389	
	0.625	0.822	0.828	0.825	0.825	0.001732	99.75816203	0.241837969	
	0.312	0.831	0.817	0.826	0.824667	0.004096	99.7178557	0.282144297	
Old 5 [6]	10	0.018	0.017	0.019	0.018	0.000577	2.176541717	97.82345828	1.15 ± 0.03
	5	0.019	0.018	0.019	0.018667	0.000333	2.257154373	97.74284563	
	2.5	0.188	0.241	0.214	0.214333	0.015301	25.91696896	74.08303104	
	1.25	0.38	0.326	0.348	0.351333	0.015677	42.48286981	57.51713019	
	0.625	0.716	0.749	0.732	0.732333	0.009528	88.55300282	11.44699718	
	0.312	0.811	0.796	0.809	0.805333	0.004702	97.38008867	2.619911326	
	10	0.083	0.055	0.072	0.07	0.008145	8.4643289	91.5356711	

**Schif** [9]	5	0.157	0.192	0.184	0.177667	0.010588	21.48327287	78.51672713	3.16 ± 0.13
	2.5	0.438	0.462	0.449	0.449667	0.006936	54.3732366	45.6267634	
	1.25	0.693	0.738	0.733	0.721333	0.01424	87.22289399	12.77710601	
	0.625	0.83	0.821	0.823	0.824667	0.002728	99.7178557	0.282144297	
	0.312	0.82	0.837	0.824	0.827	0.005132	100	0	

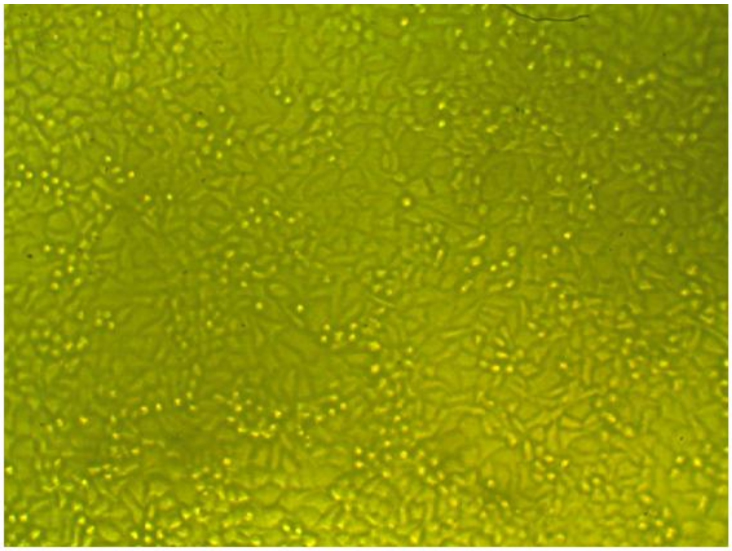

control
Mcf7 cells

Organism : *Homo sapiens*, human
Tissue : mammary gland, breast; derived from metastatic site: pleural effusion
Cell Type : epithelial
Culture Properties : adherent
Disease : adenocarcinoma
ATCC : HTB-22

Effect of sample Se NPs on Mcf7 cells at different concentration

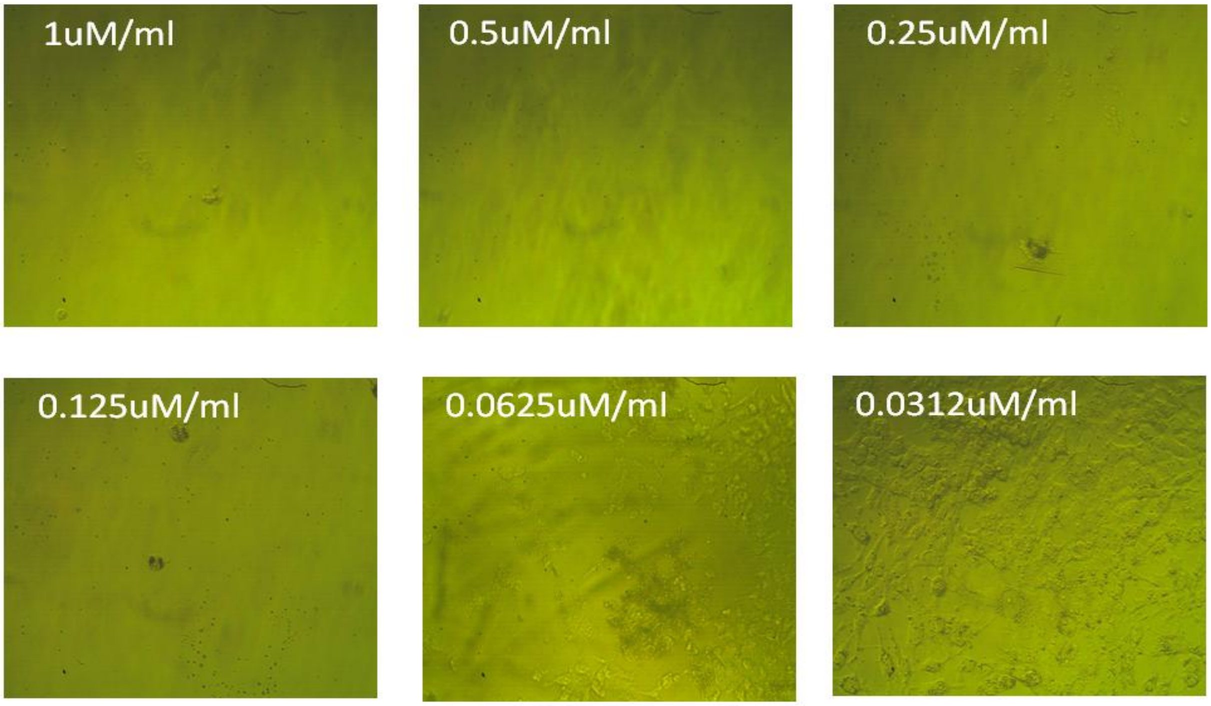

Effect of sample 1 on Mcf7 cells at different concentration

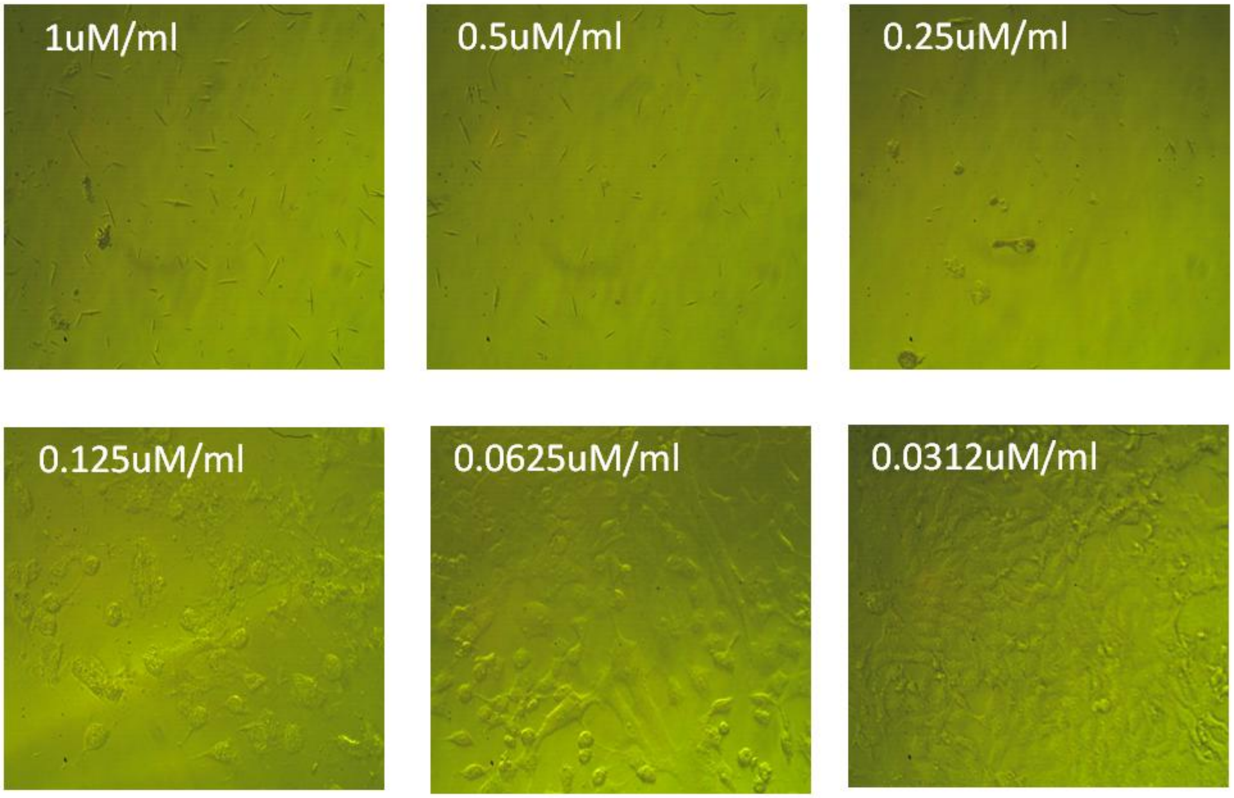

Effect of sample 2 on Mcf7 cells at different concentration

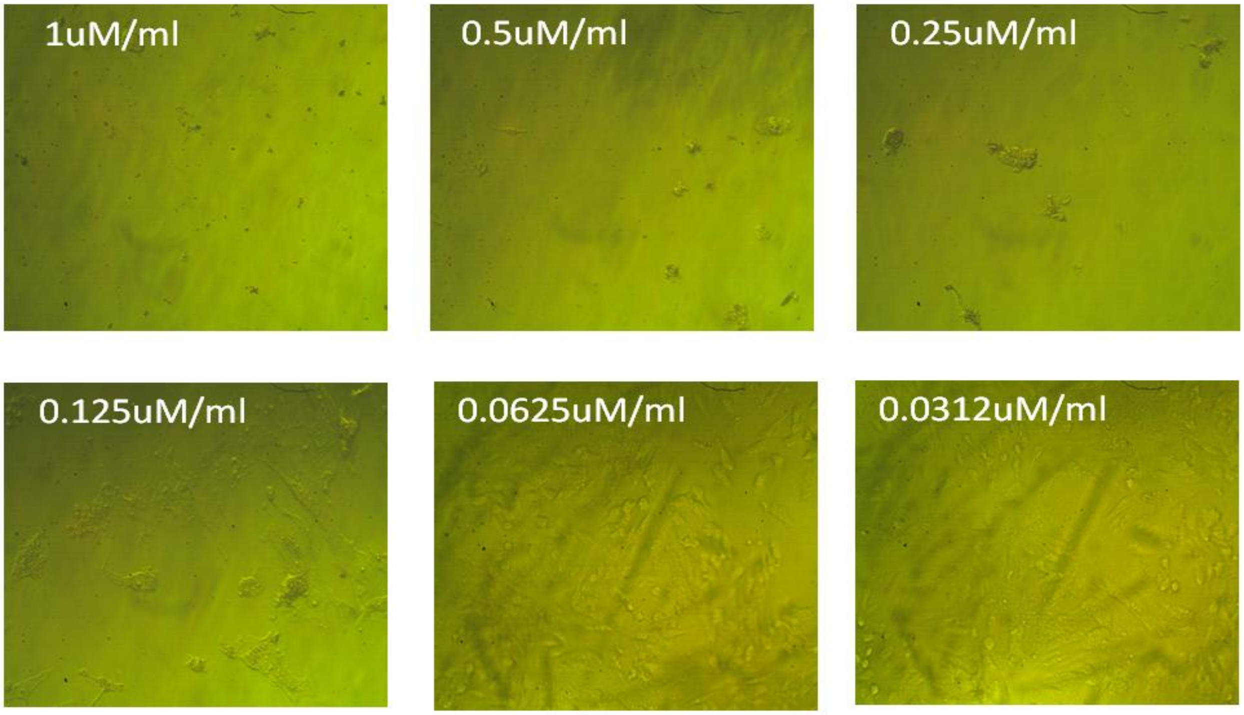

Effect of sample 3 on Mcf7 cells at different concentration

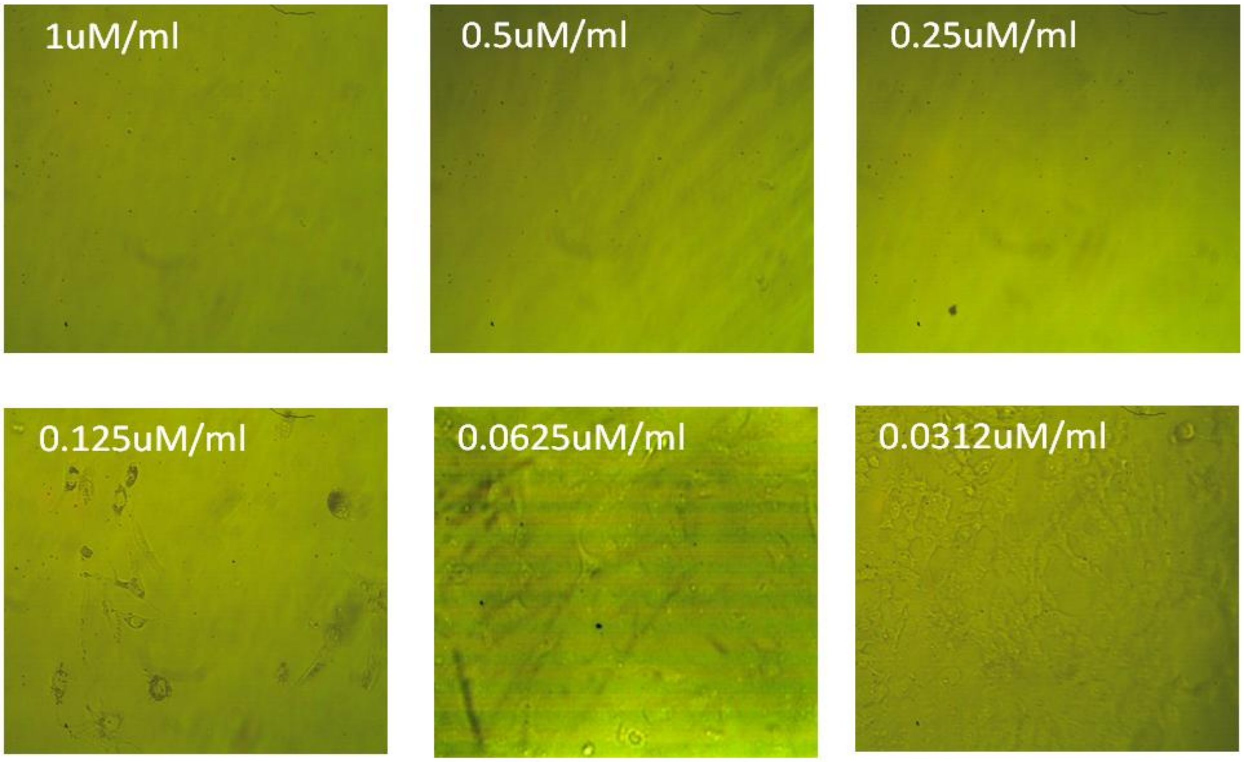

Effect of sample 4 on Mcf7 cells at different concentration

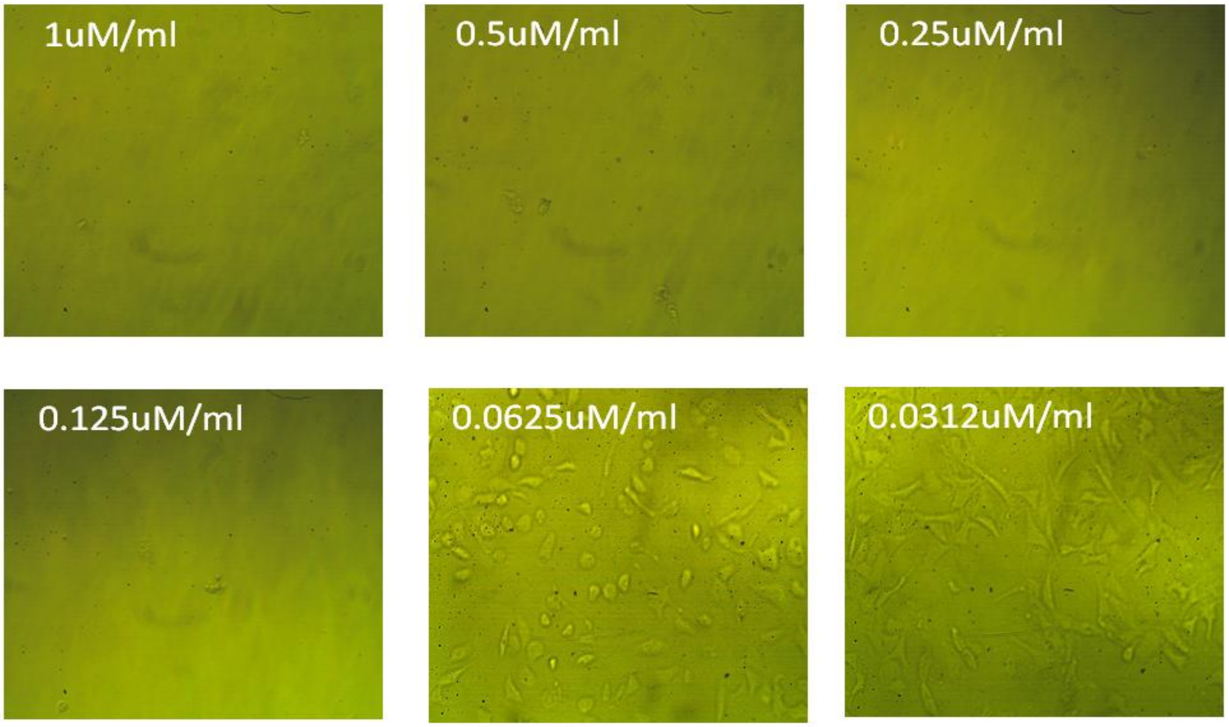

Effect of sample 5 on Mcf7 cells at different concentration

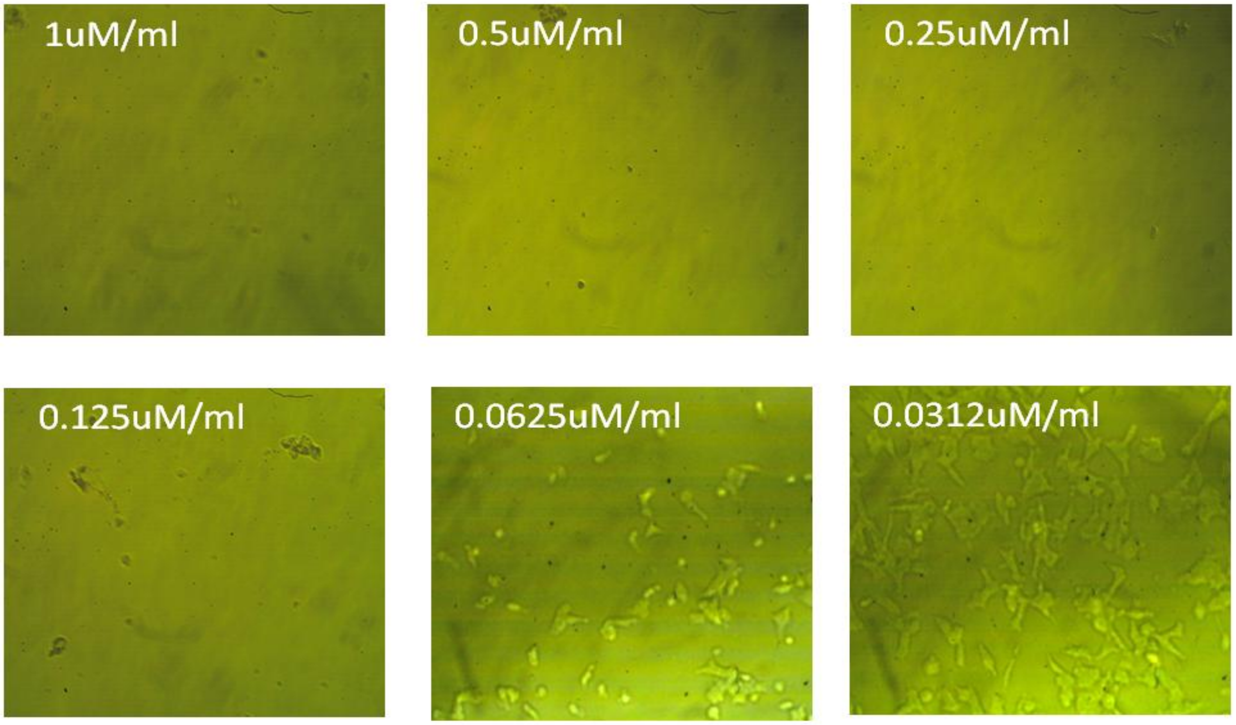

Effect of sample 6 on Mcf7 cells at different concentration

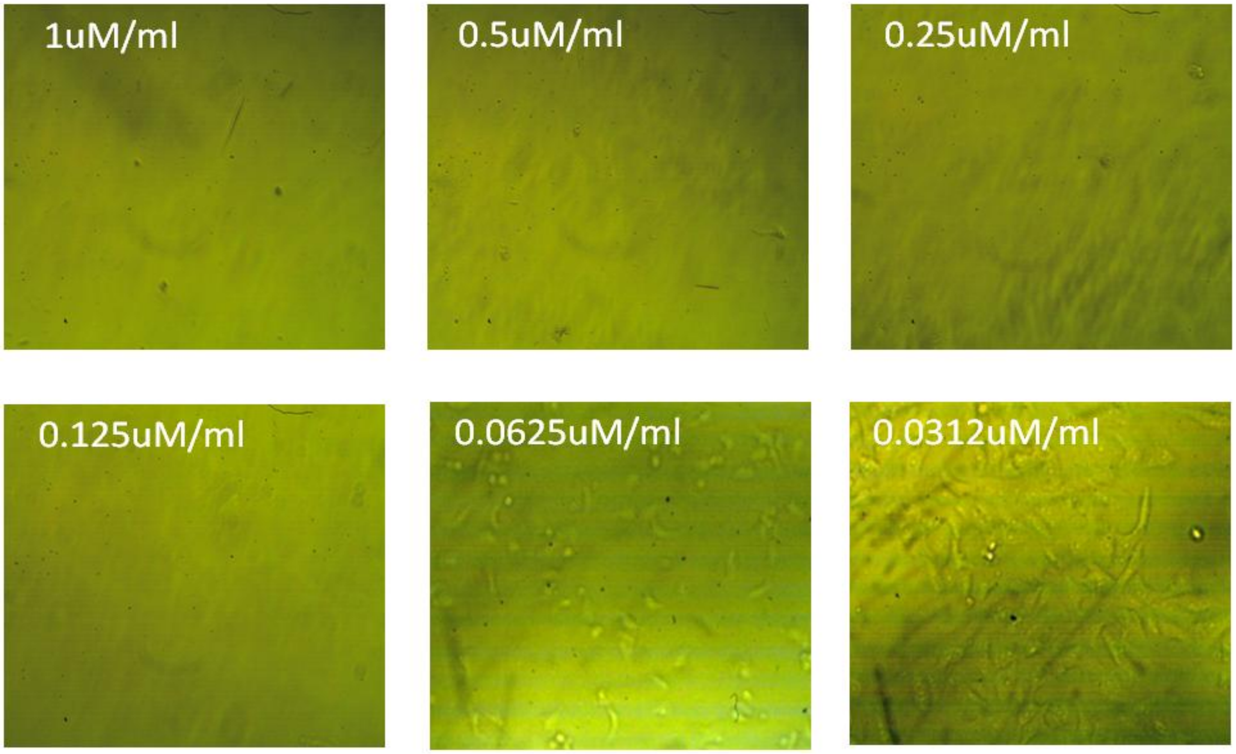

Effect of sample Old 1 on Mcf7 cells at different concentration

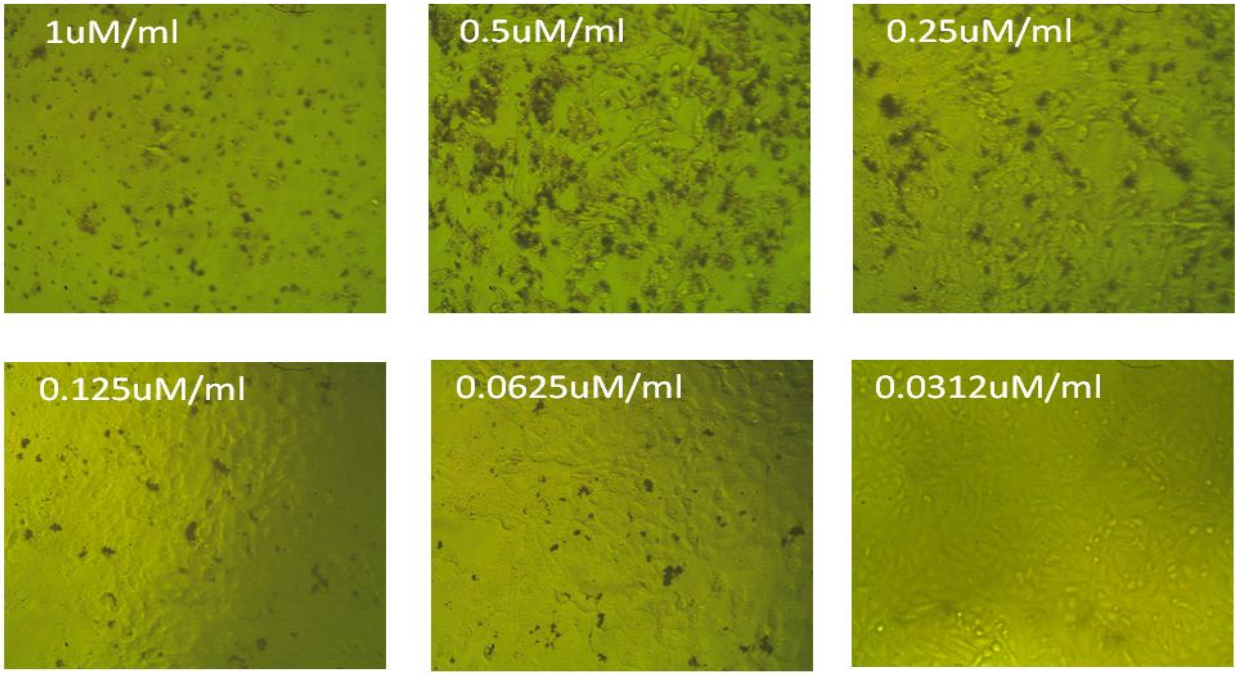

Effect of sample Old 2 on Mcf7 cells at different concentration

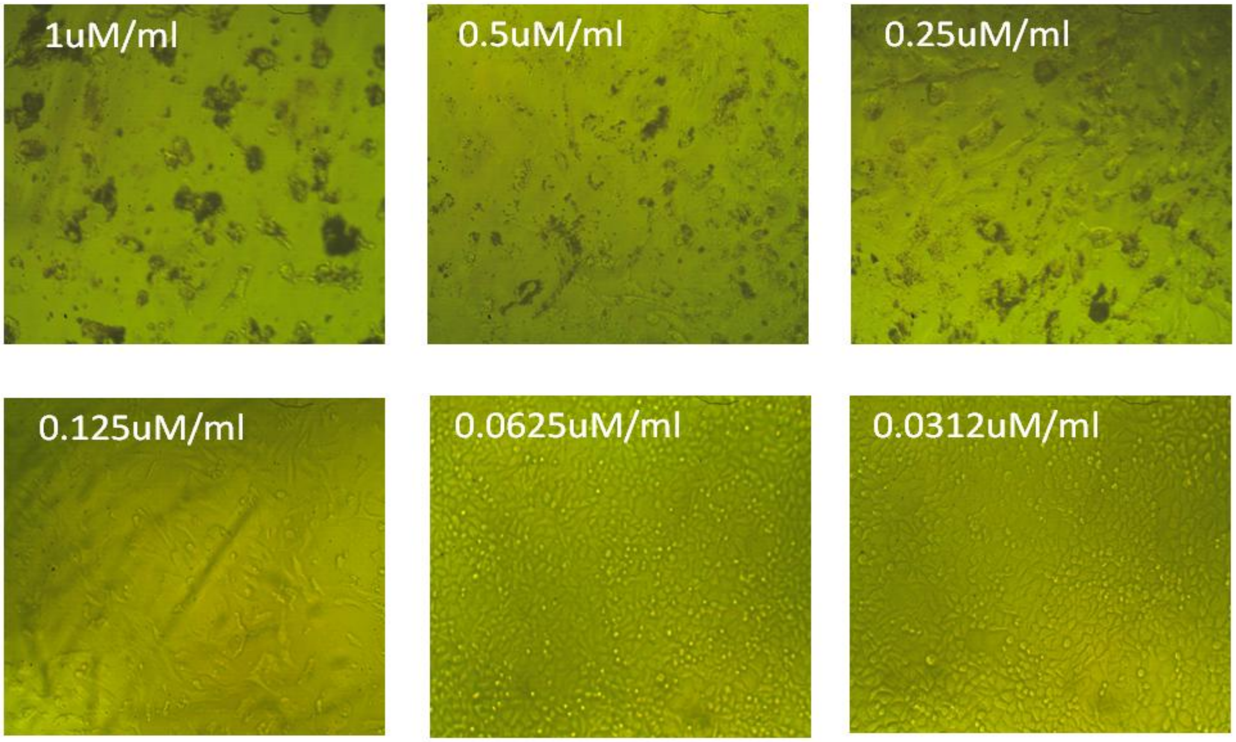

Effect of sample Old 3 on Mcf7 cells at different concentration

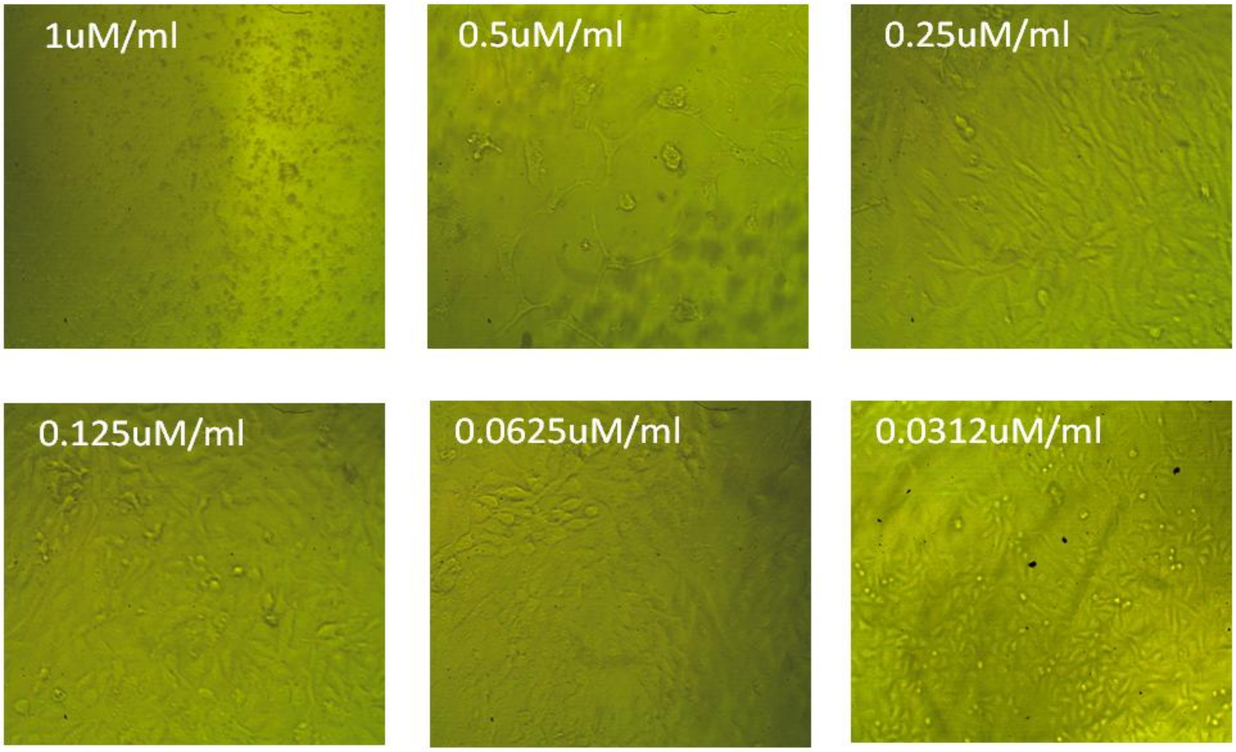

Effect of sample Old 4 on Mcf7 cells at different concentration

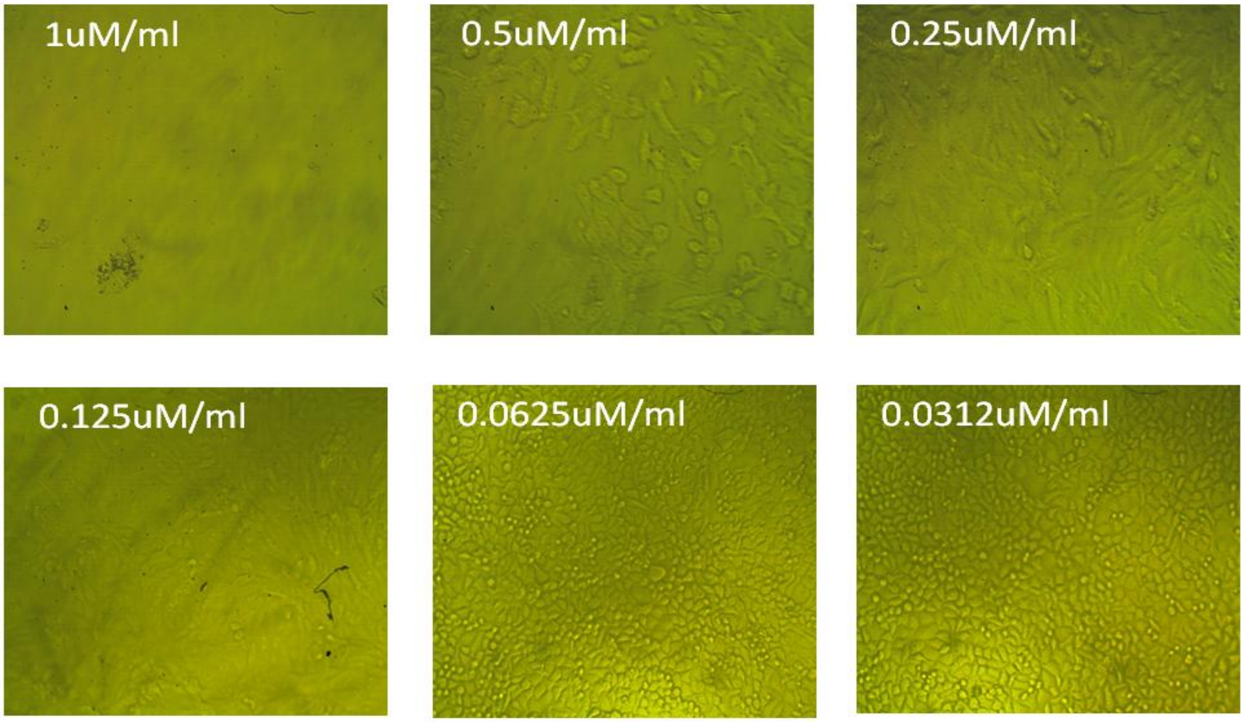

Effect of sample Old 5 on Mcf7 cells at different concentration

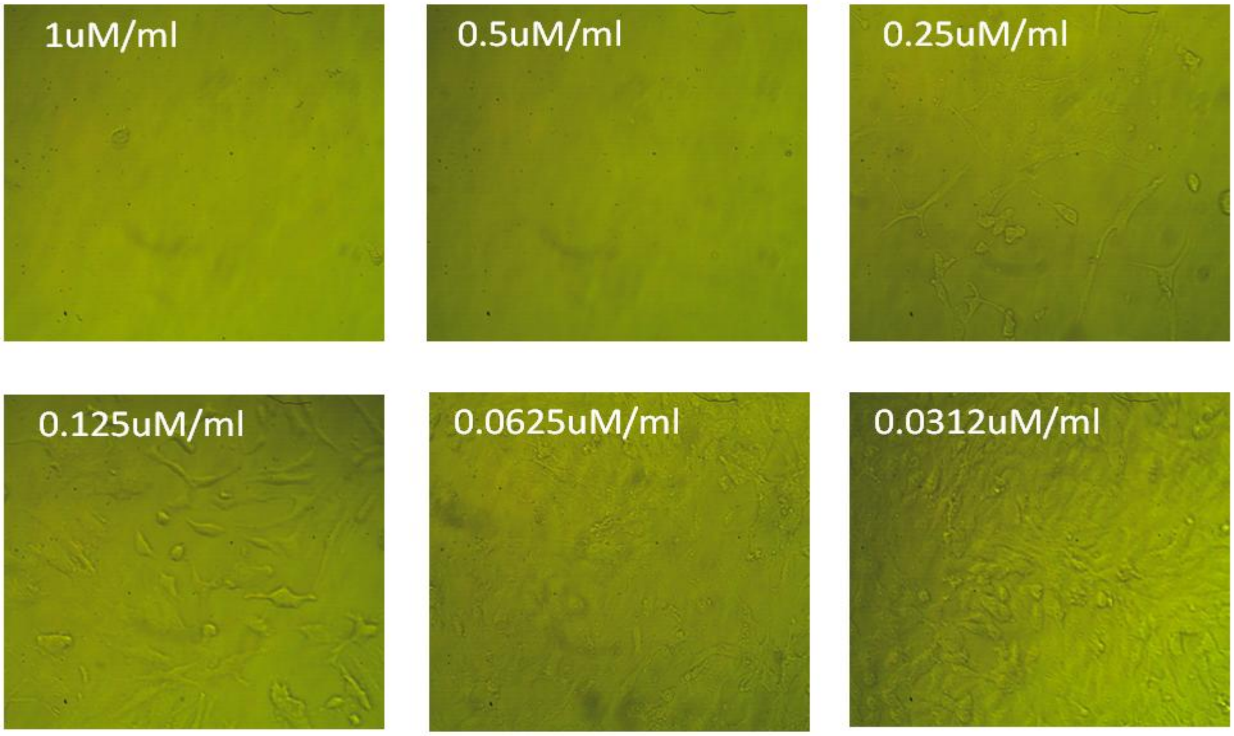

Effect of sample Schif on Mcf7 cells at different concentration

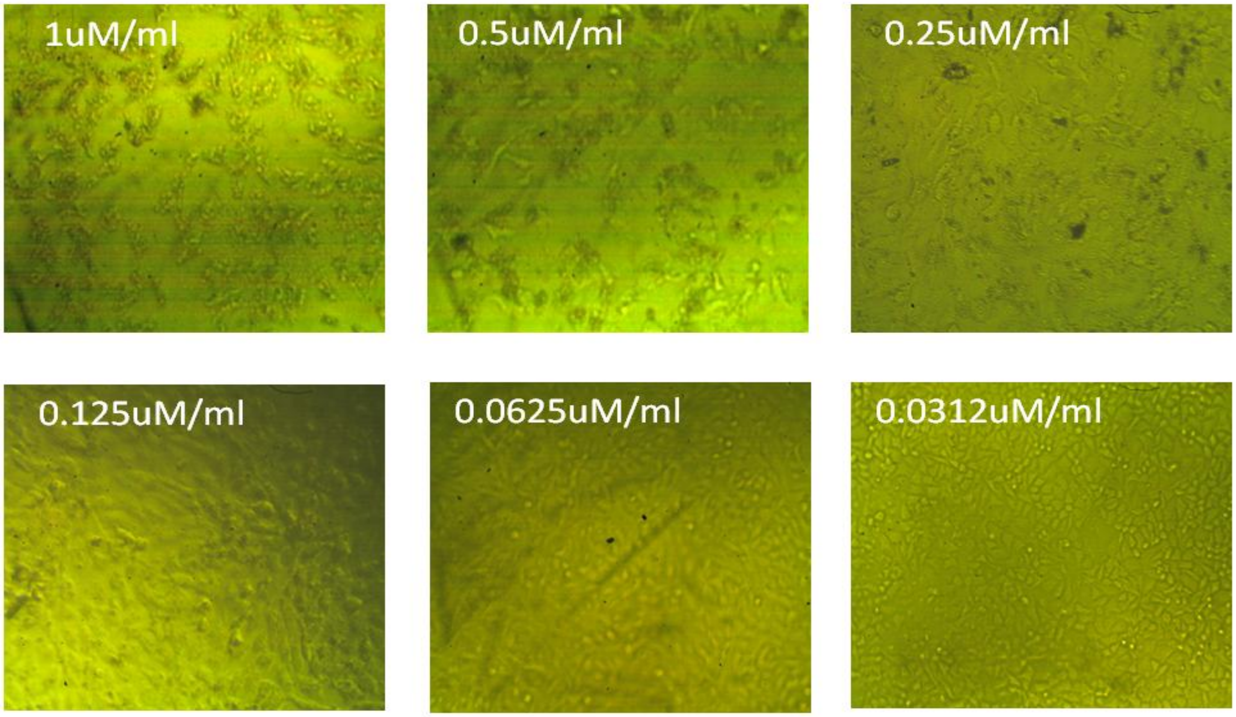

ID	uM/ml	O.D			Mean O.D	±SE	Viability %	Toxicity %	IC50 ± SD
HepG-2	--------	0.782	0.759	0.763	0.768	0.007095	100	0	uM
Se NPs	1	0.018	0.017	0.019	0.018	0.000577	2.34375	97.65625	0.04 ± 0
	0.5	0.019	0.017	0.019	0.018333	0.000667	2.387152778	97.61284722	
	0.25	0.019	0.019	0.019	0.019	0	2.473958333	97.52604167	
	0.125	0.019	0.019	0.02	0.019333	0.000333	2.517361111	97.48263889	
	0.062	0.136	0.11	0.106	0.117333	0.009404	15.27777778	84.72222222	
	0.031	0.437	0.478	0.444	0.453	0.012662	58.984375	41.015625	
1 [9NPs]	1	0.018	0.017	0.019	0.018	0.000577	2.34375	97.65625	0.05 ± 0
	0.5	0.018	0.017	0.018	0.017667	0.000333	2.300347222	97.69965278	
	0.25	0.017	0.016	0.02	0.017667	0.001202	2.300347222	97.69965278	
	0.125	0.022	0.031	0.028	0.027	0.002646	3.515625	96.484375	
	0.062	0.26	0.231	0.249	0.246667	0.008452	32.11805556	67.88194444	
	0.031	0.517	0.501	0.473	0.497	0.012858	64.71354167	35.28645833	
2 [7NPs]	1	0.019	0.015	0.016	0.016667	0.001202	2.170138889	97.82986111	0.03 ± 0
	0.5	0.016	0.018	0.016	0.016667	0.000667	2.170138889	97.82986111	
	0.25	0.022	0.018	0.019	0.019667	0.001202	2.560763889	97.43923611	
	0.125	0.022	0.026	0.021	0.023	0.001528	2.994791667	97.00520833	
	0.062	0.093	0.118	0.107	0.106	0.007234	13.80208333	86.19791667	
	0.031	0.455	0.421	0.419	0.431667	0.011681	56.20659722	43.79340278	
3 [5NPs]	1	0.017	0.019	0.018	0.018	0.000577	2.34375	97.65625	0.09 ± 0
	0.5	0.018	0.017	0.019	0.018	0.000577	2.34375	97.65625	
	0.25	0.063	0.048	0.055	0.055333	0.004333	7.204861111	92.79513889	
	0.125	0.162	0.149	0.137	0.149333	0.007219	19.44444444	80.55555556	
	0.062	0.483	0.529	0.541	0.517667	0.017676	67.40451389	32.59548611	
	0.031	0.699	0.732	0.701	0.710667	0.010682	92.53472222	7.465277778	
4 [6NPs]	1	0.018	0.016	0.019	0.017667	0.000882	2.300347222	97.69965278	0.07 ± 0
	0.5	0.019	0.017	0.018	0.018	0.000577	2.34375	97.65625	
	0.25	0.02	0.02	0.017	0.019	0.001	2.473958333	97.52604167	
	0.125	0.083	0.069	0.091	0.081	0.006429	10.546875	89.453125	
	0.062	0.392	0.437	0.417	0.415333	0.013017	54.07986111	45.92013889	
	0.031	0.642	0.666	0.682	0.663333	0.011624	86.37152778	13.62847222	
5 [4NPs]	1	0.017	0.016	0.016	0.016333	0.000333	2.126736111	97.87326389	0.03 ± 0.01
	0.5	0.017	0.016	0.018	0.017	0.000577	2.213541667	97.78645833	
	0.25	0.019	0.017	0.017	0.017667	0.000667	2.300347222	97.69965278	
	0.125	0.022	0.051	0.028	0.033667	0.008838	4.383680556	95.61631944	
	0.062	0.11	0.142	0.108	0.12	0.011015	15.625	84.375	
	0.031	0.36	0.411	0.389	0.386667	0.014769	50.34722222	49.65277778	
6 [8NPs]	1	0.017	0.019	0.019	0.018333	0.000667	2.387152778	97.61284722	0.05 ± 0
	0.5	0.019	0.015	0.017	0.017	0.001155	2.213541667	97.78645833	
	0.25	0.018	0.019	0.018	0.018333	0.000333	2.387152778	97.61284722	
	0.125	0.019	0.023	0.024	0.022	0.001528	2.864583333	97.13541667	
	0.062	0.225	0.216	0.256	0.232333	0.012115	30.25173611	69.74826389	
	0.031	0.524	0.515	0.476	0.505	0.014731	65.75520833	34.24479167	

ID	uM/ml	O.D			Mean O.D	±SE	Viability %	Toxicity %	IC50 ± SD
HepG2	--------	0.782	0.759	0.763	0.768	0.007095	100	0	uM
Old 1 [7]	10	0.052	0.044	0.039	0.045	0.003786	5.859375	94.140625	3.38 ± 0.11
	5	0.162	0.197	0.183	0.180667	0.010171	23.52430556	76.47569444	
	2.5	0.427	0.448	0.469	0.448	0.012124	58.33333333	41.66666667	
	1.25	0.73	0.751	0.699	0.726667	0.015103	94.61805556	5.381944444	
	0.625	0.747	0.762	0.777	0.762	0.00866	99.21875	0.78125	
	0.312	0.772	0.756	0.759	0.762333	0.00491	99.26215278	0.737847222	
Old 2 [8]	10	0.071	0.094	0.118	0.094333	0.013569	12.28298611	87.71701389	2.1 ± 0.09
	5	0.116	0.143	0.139	0.132667	0.008413	17.27430556	82.72569444	
	2.5	0.362	0.275	0.299	0.312	0.025942	40.625	59.375	
	1.25	0.527	0.519	0.546	0.530667	0.008007	69.09722222	30.90277778	
	0.625	0.718	0.742	0.74	0.733333	0.007688	95.48611111	4.513888889	
	0.312	0.749	0.762	0.771	0.760667	0.006386	99.04513889	0.954861111	
Old 3 [4]	10	0.018	0.016	0.019	0.017667	0.000882	2.300347222	97.69965278	1.07 ± 0.03
	5	0.018	0.018	0.019	0.018333	0.000333	2.387152778	97.61284722	
	2.5	0.026	0.024	0.023	0.024333	0.000882	3.168402778	96.83159722	
	1.25	0.261	0.302	0.288	0.283667	0.012032	36.93576389	63.06423611	
	0.625	0.645	0.662	0.671	0.659333	0.007623	85.85069444	14.14930556	
	0.312	0.764	0.732	0.722	0.739333	0.012667	96.26736111	3.732638889	
Old 4 [5]	10	0.019	0.019	0.019	0.019	0	2.473958333	97.52604167	4.07 ± 0.08
	5	0.216	0.222	0.239	0.225667	0.006888	29.38368056	70.61631944	
	2.5	0.714	0.739	0.744	0.732333	0.00928	95.35590278	4.644097222	
	1.25	0.756	0.781	0.761	0.766	0.007638	99.73958333	0.260416667	
	0.625	0.76	0.782	0.759	0.767	0.007506	99.86979167	0.130208333	
	0.312	0.768	0.763	0.77	0.767	0.002082	99.86979167	0.130208333	
Old 5 [6]	10	0.018	0.016	0.019	0.017667	0.000882	2.300347222	97.69965278	1.97 ± 0.05
	5	0.019	0.018	0.02	0.019	0.000577	2.473958333	97.52604167	
	2.5	0.22	0.261	0.248	0.243	0.012097	31.640625	68.359375	
	1.25	0.583	0.552	0.579	0.571333	0.009735	74.39236111	25.60763889	
	0.625	0.752	0.77	0.749	0.757	0.006557	98.56770833	1.432291667	
	0.312	0.75	0.756	0.768	0.758	0.005292	98.69791667	1.302083333	
Schif [9]	10	0.07	0.062	0.088	0.073333	0.007688	9.548611111	90.45138889	3.47 ± 0.09
	5	0.217	0.193	0.226	0.212	0.009849	27.60416667	72.39583333	
	2.5	0.421	0.457	0.45	0.442667	0.01102	57.63888889	42.36111111	
	1.25	0.718	0.726	0.746	0.73	0.008327	95.05208333	4.947916667	
	0.625	0.769	0.76	0.751	0.76	0.005196	98.95833333	1.041666667	
	0.312	0.754	0.78	0.762	0.765333	0.007688	99.65277778	0.347222222	

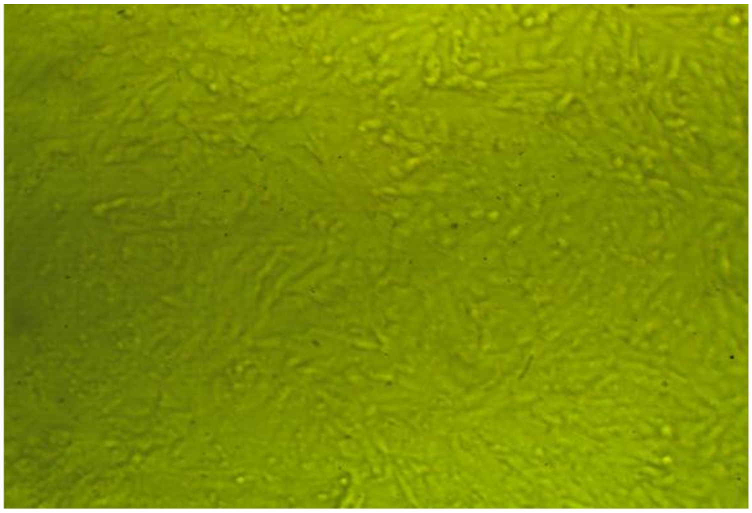

control
HepG2 cells

Organism :	*Homo sapiens*, human
Tissue :	liver
Cell Type :	epithelial
Culture Properties :	adherent
Disease :	hepatocellular carcinoma
ATCC :	HB-8065

Effect of sample Se NPs on HepG2 cells at different concentration

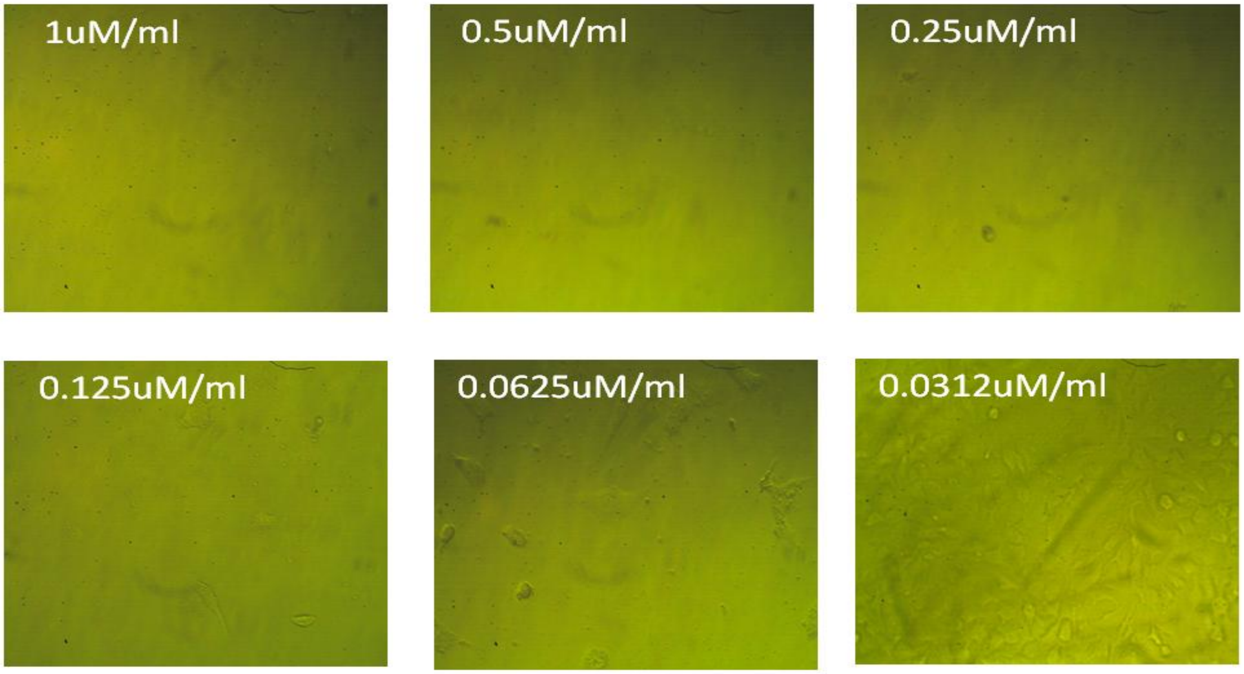

Effect of sample 1 on HepG2 cells at different concentration

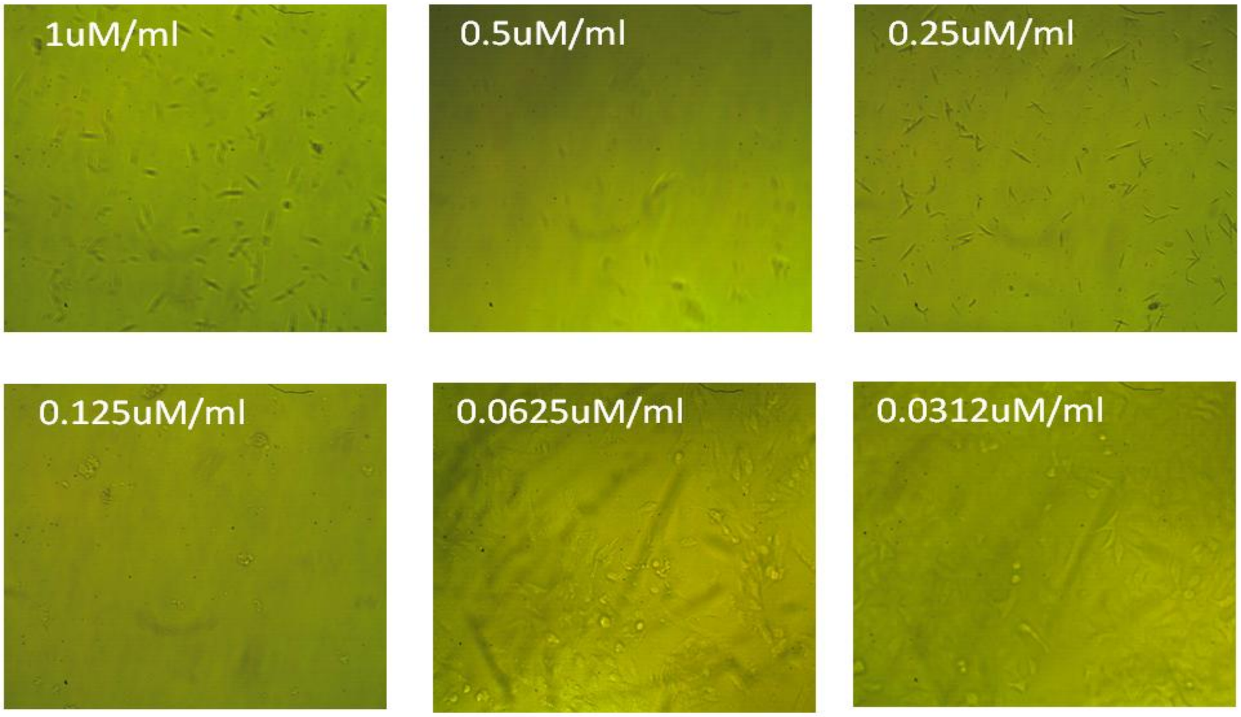

Effect of sample 2 on HepG2 cells at different concentration

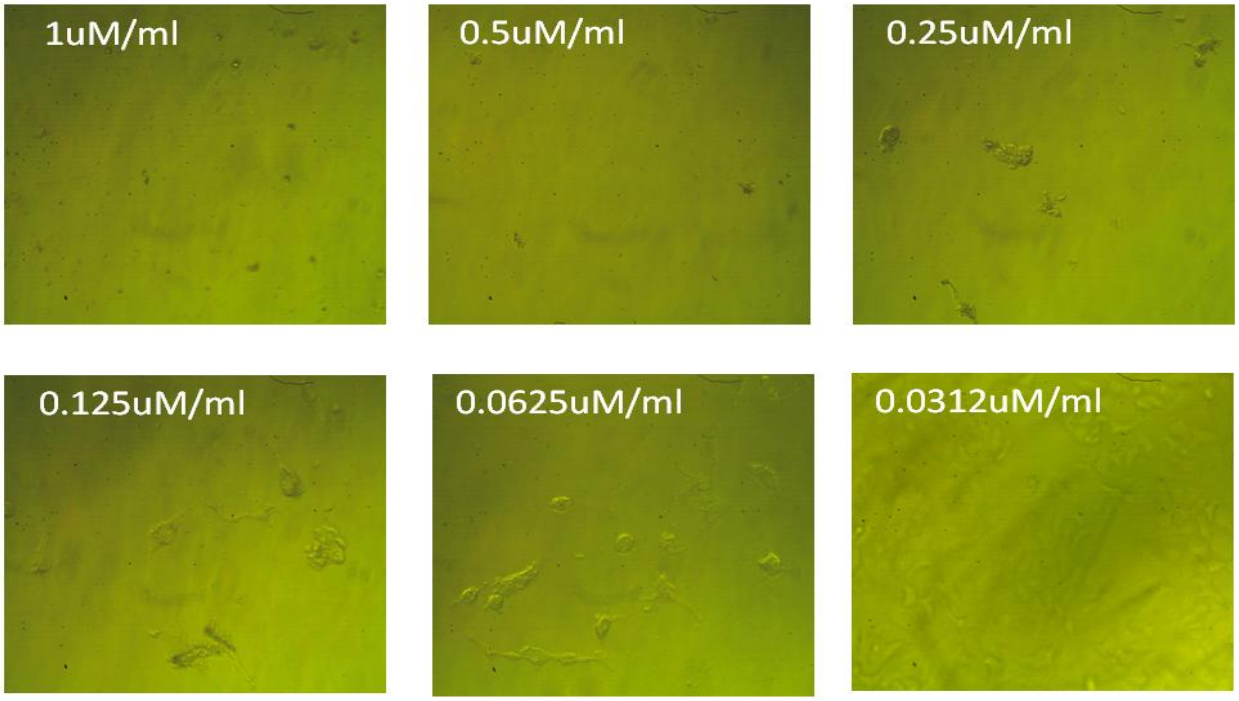

Effect of sample 3 on HepG2 cells at different concentration

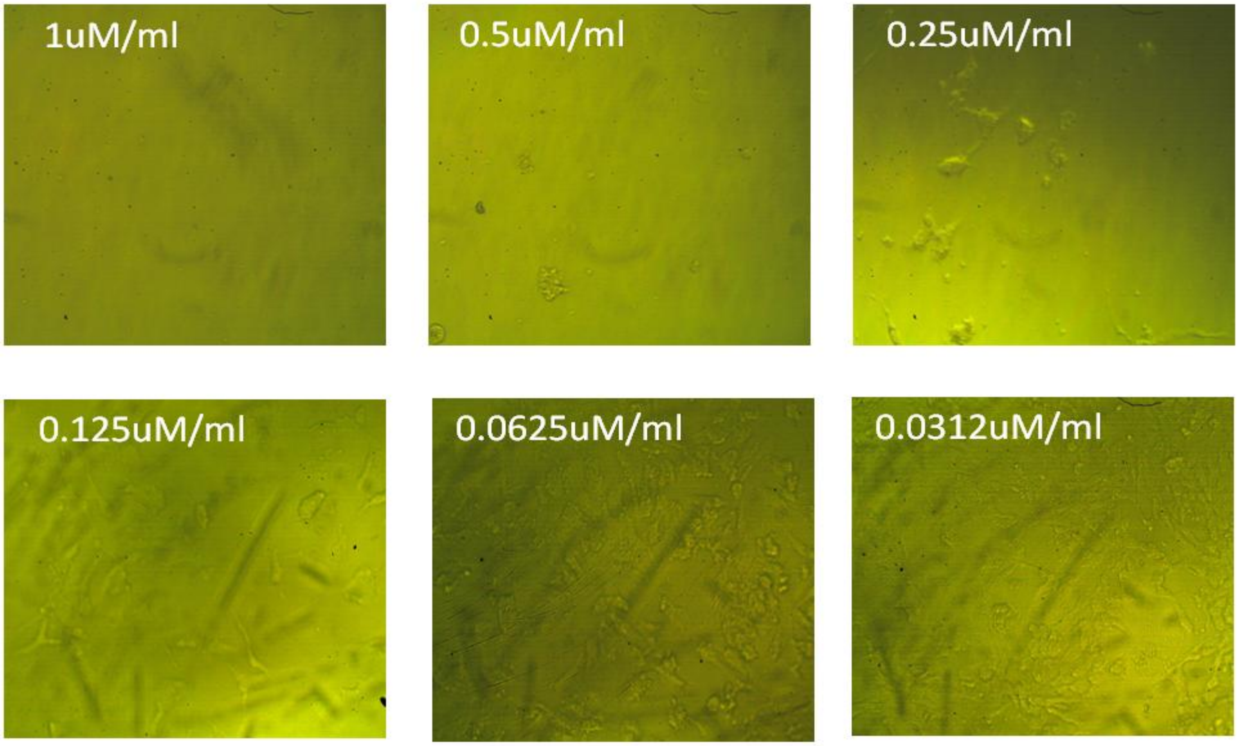

Effect of sample 4 on HepG2 cells at different concentration

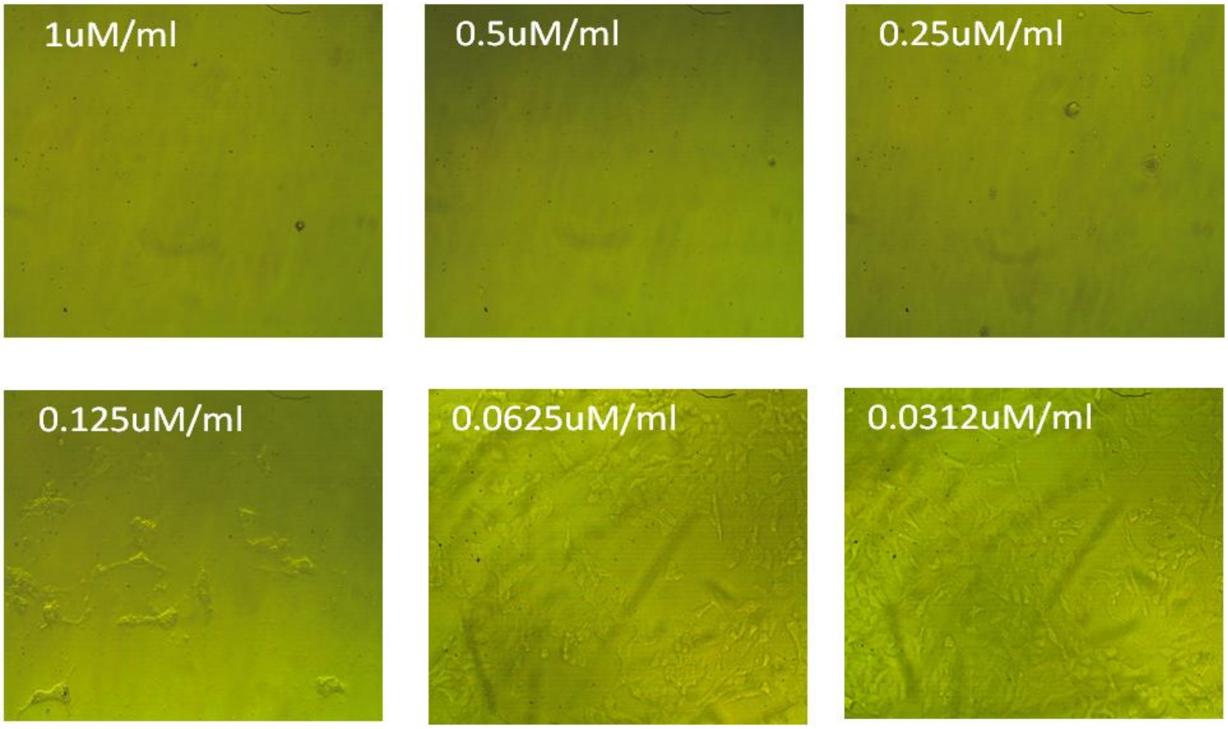

Effect of sample 5 on HepG2 cells at different concentration

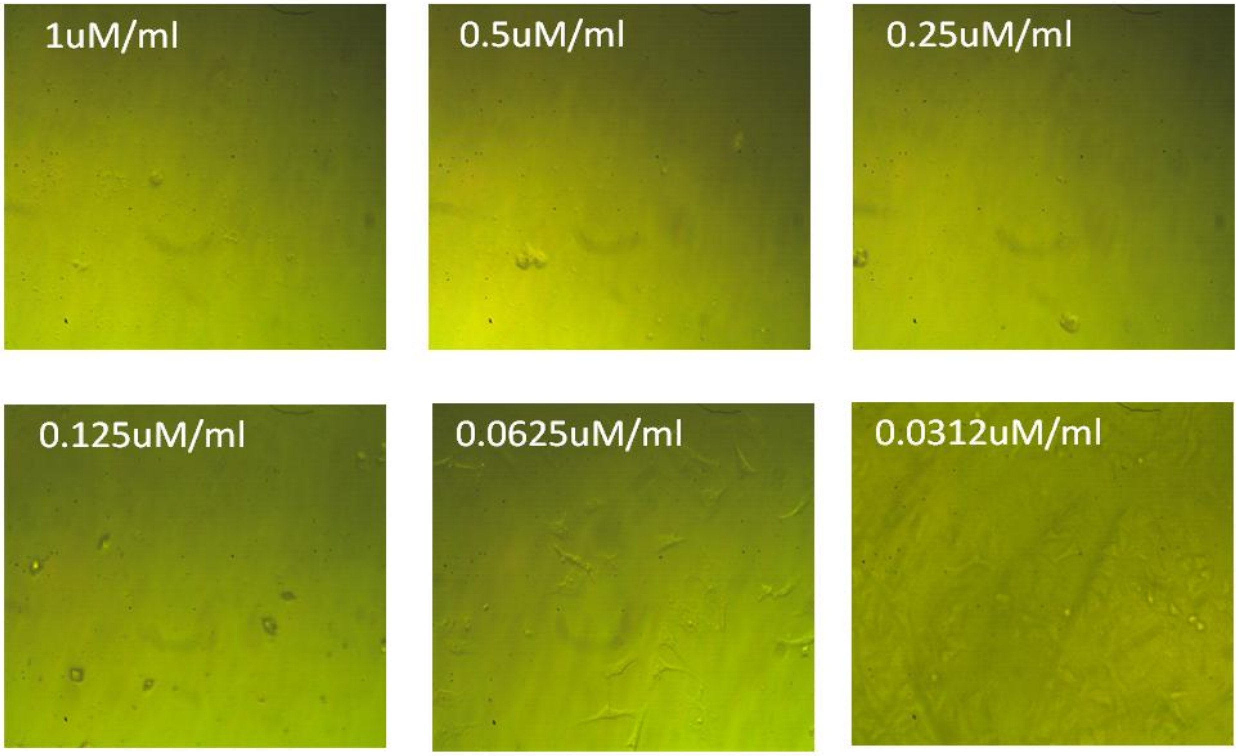

Effect of sample 6 on HepG2 cells at different concentration

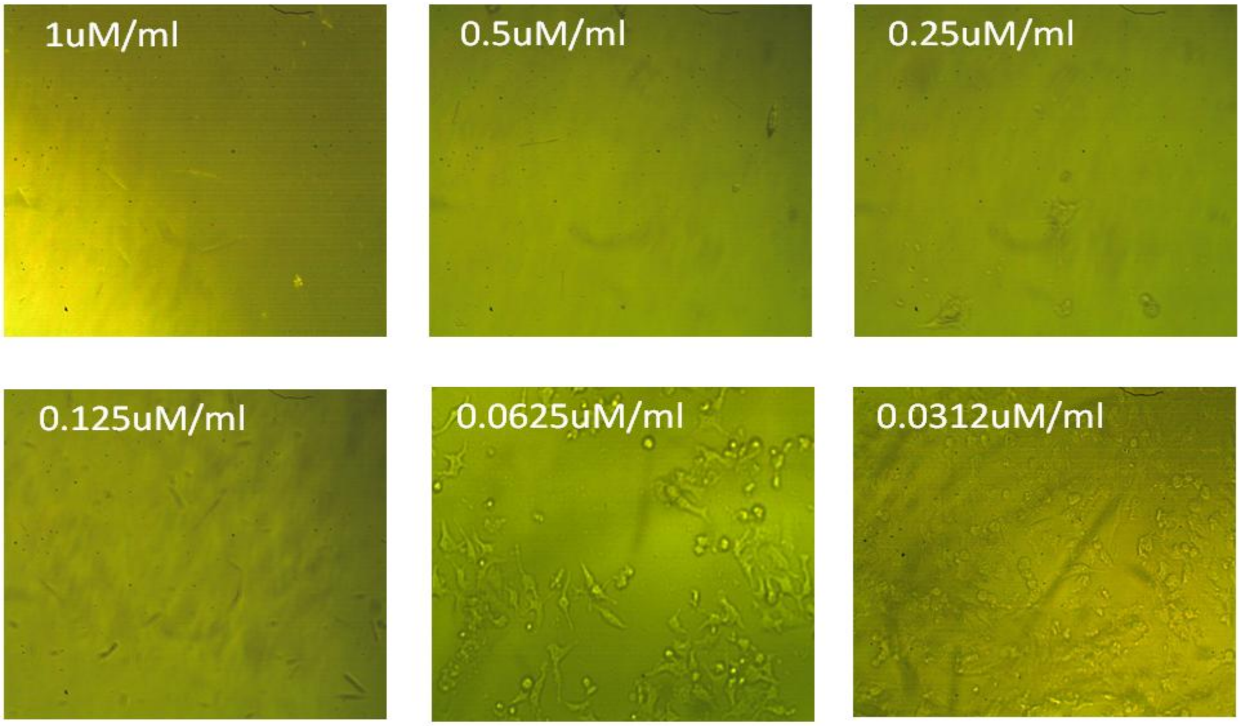

Effect of sample Old 1 on HepG2 cells at different concentration

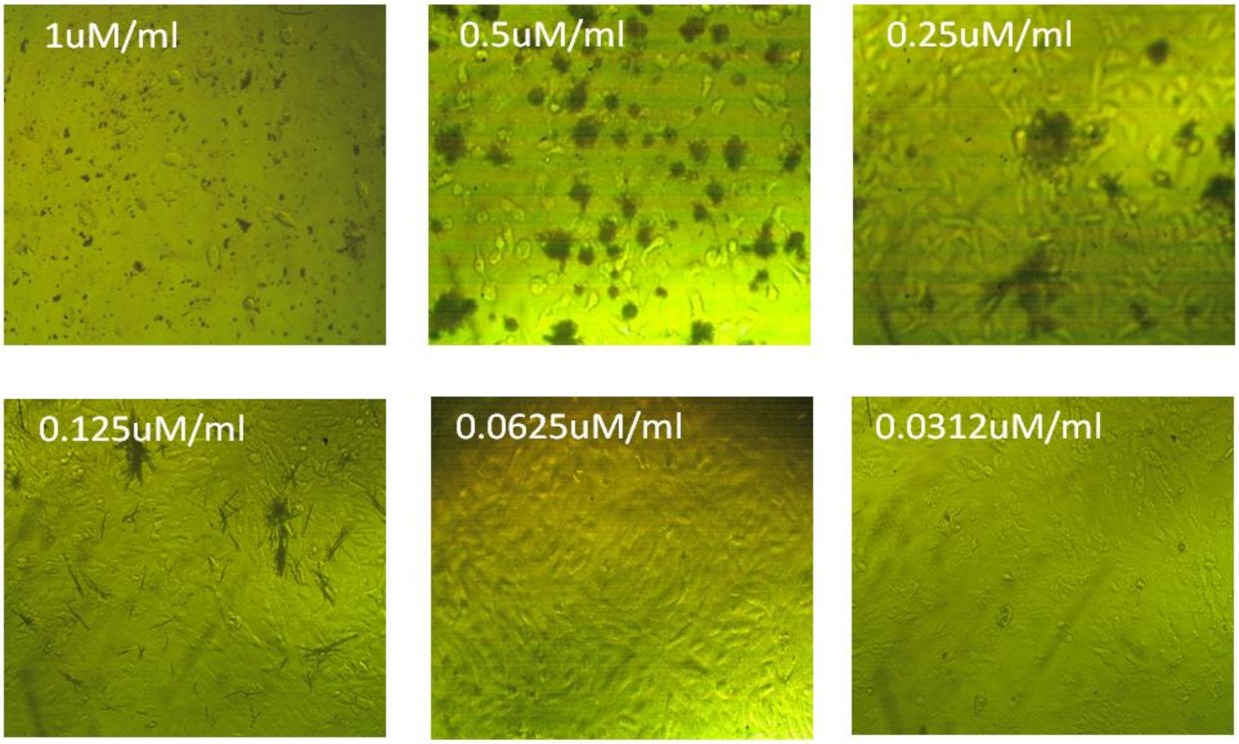

Effect of sample Old 2 on HepG2 cells at different concentration

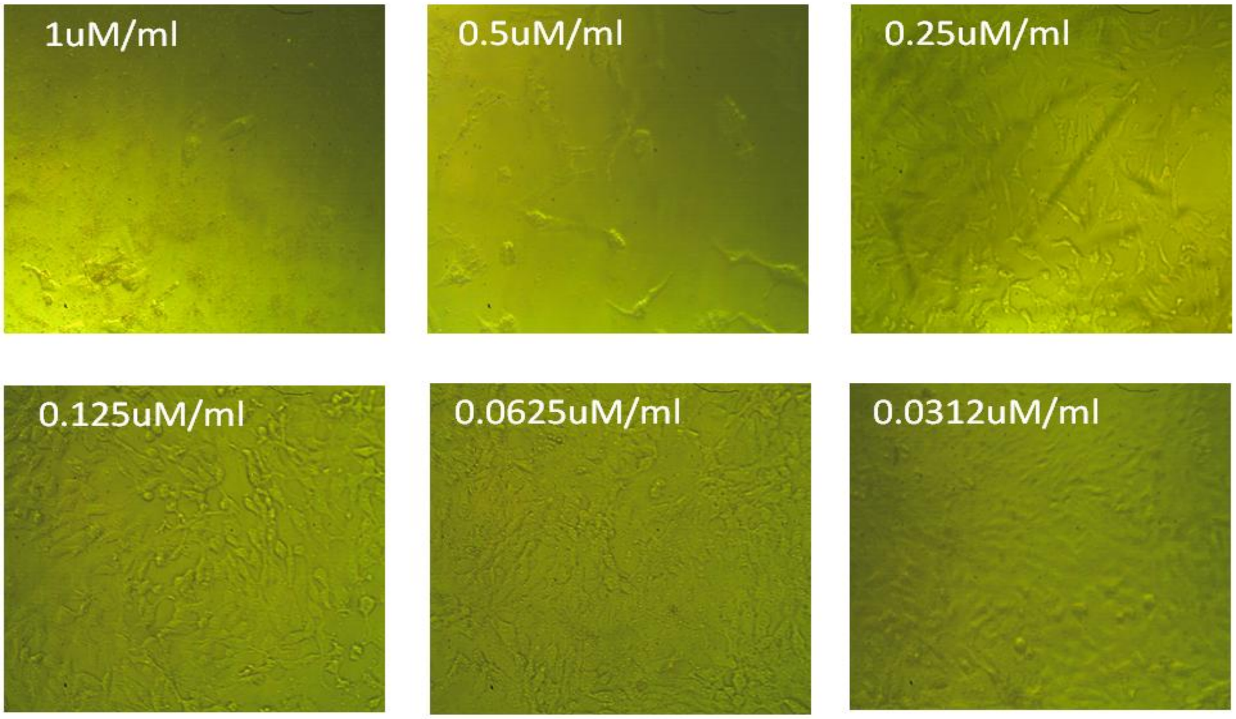

Effect of sample Old 3 on HepG2 cells at different concentration

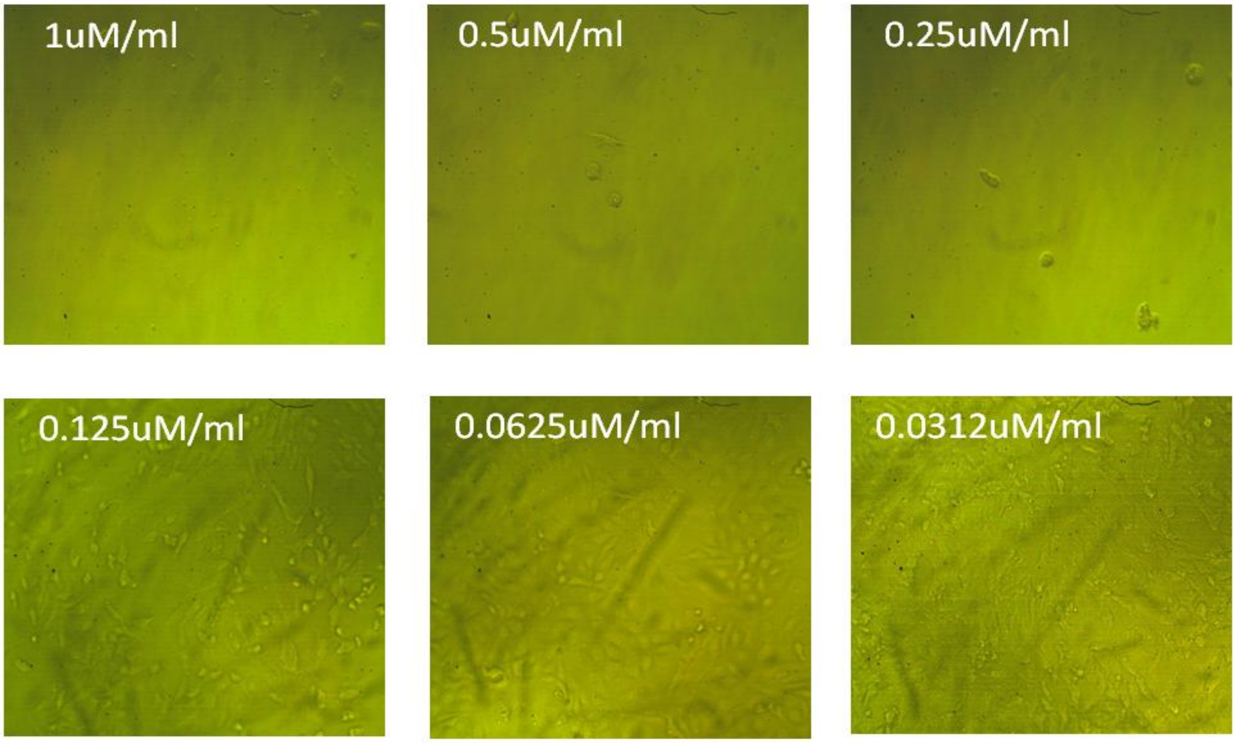

Effect of sample Old 4 on HepG2 cells at different concentration

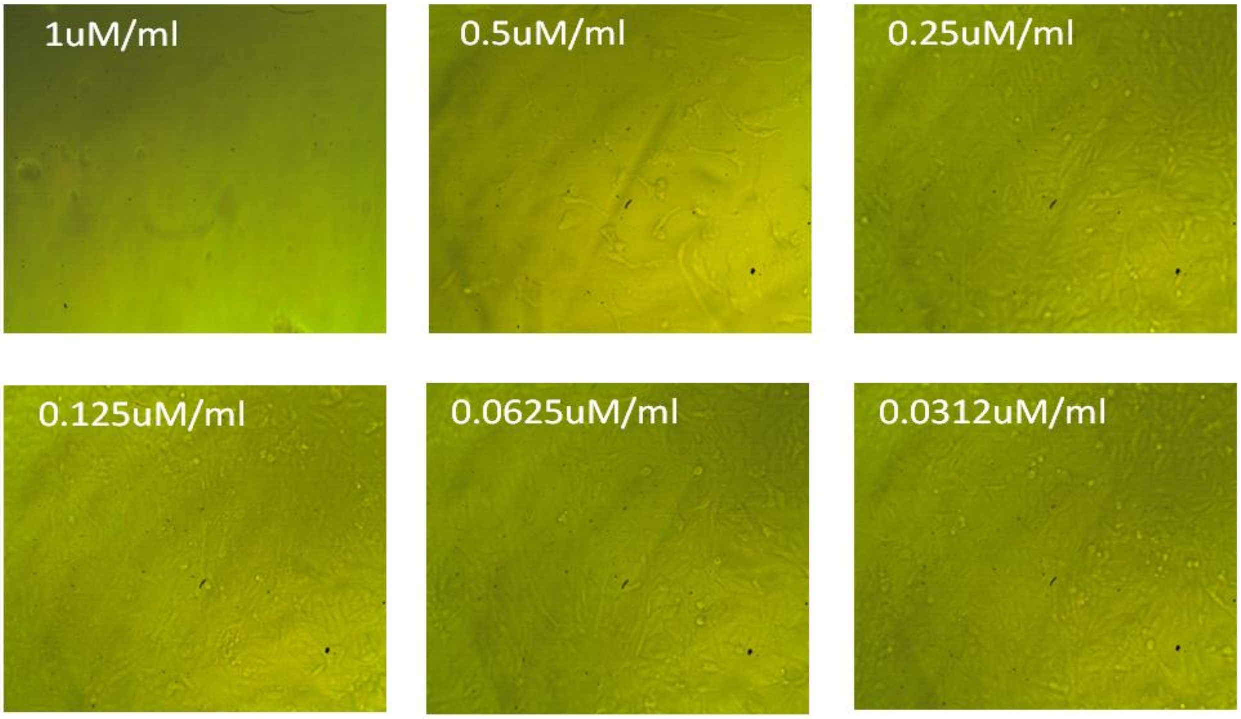

Effect of sample Old 5 on HepG2 cells at different concentration

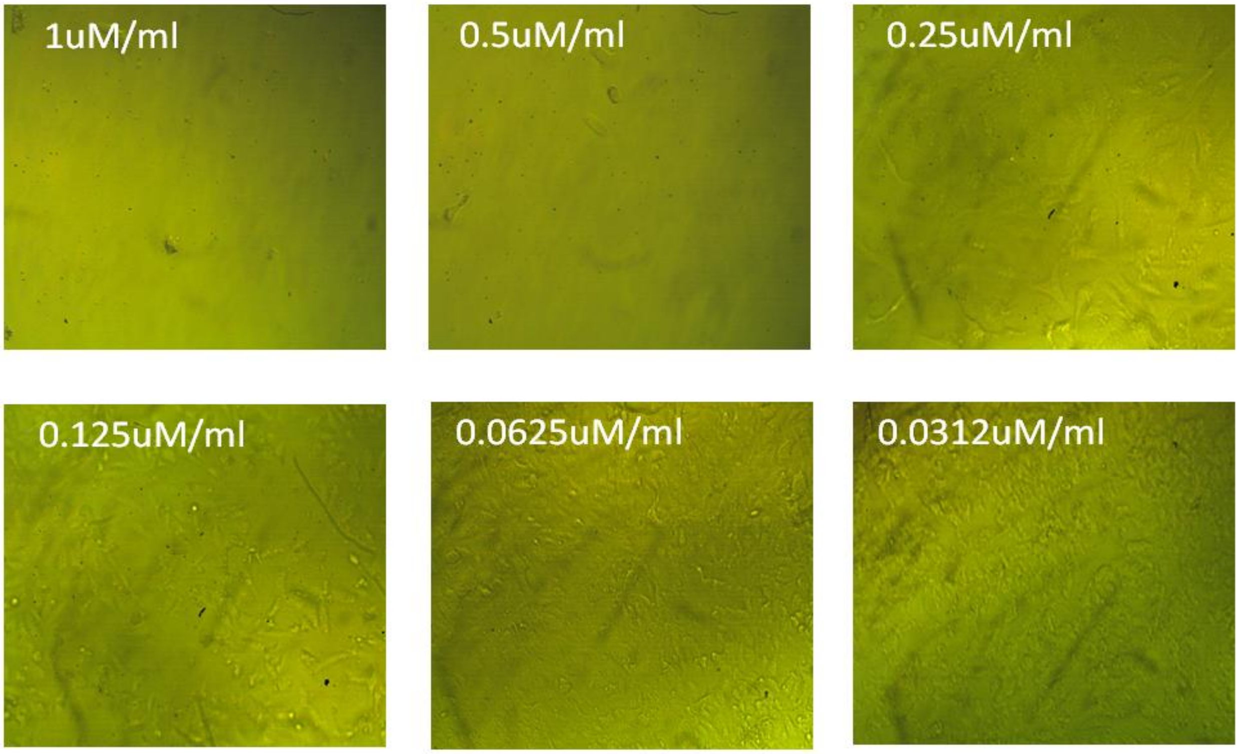

Effect of sample Schif on HepG2 cells at different concentration

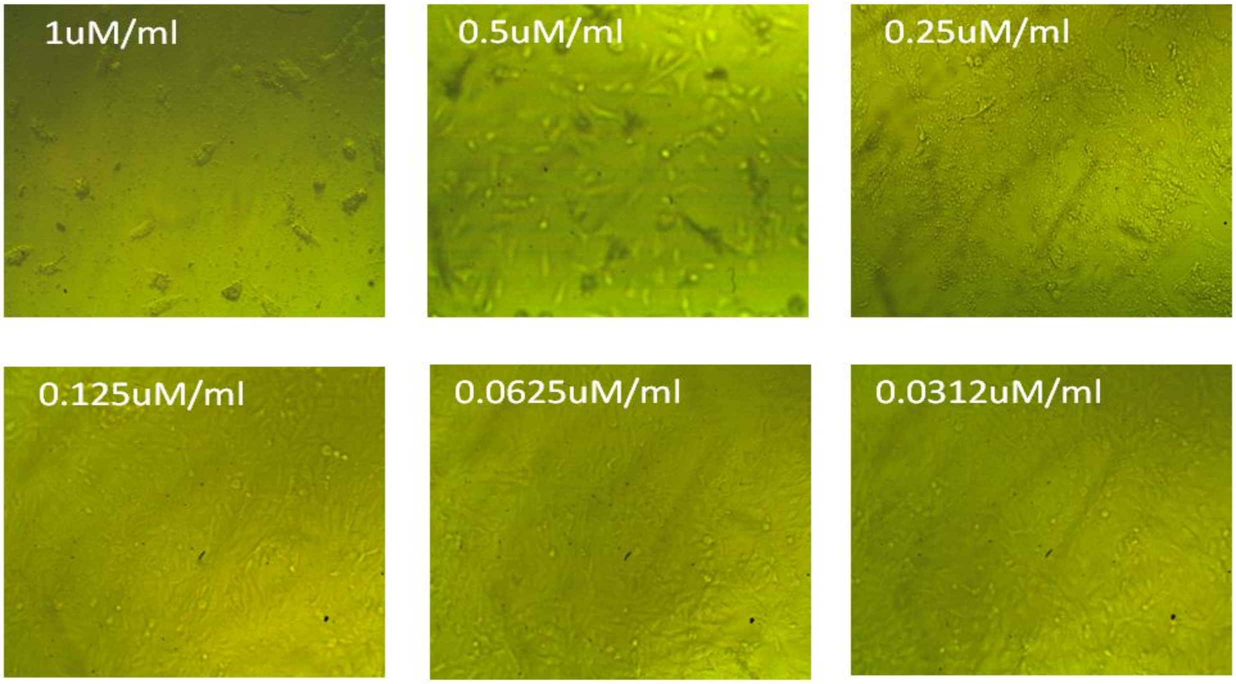

ID	uM/ml	O.D			Mean O.D	±SE	Viability %	Toxicity %	IC50 ± SD
A549	--------	0.638	0.641	0.653	0.644	0.004583	100	0	uM
Se NPs	1	0.018	0.016	0.019	0.017667	0.000882	2.743271222	97.25672878	0.04 ± 0
	0.5	0.017	0.018	0.019	0.018	0.000577	2.795031056	97.20496894	
	0.25	0.064	0.038	0.044	0.048667	0.00786	7.556935818	92.44306418	
	0.125	0.08	0.077	0.082	0.079667	0.001453	12.37060041	87.62939959	
	0.062	0.092	0.1	0.083	0.091667	0.00491	14.23395445	85.76604555	
	0.031	0.403	0.384	0.359	0.382	0.012741	59.31677019	40.68322981	
1 [9NPs]	1	0.022	0.03	0.027	0.026333	0.002333	4.089026915	95.91097308	0.1 ± 0.01
	0.5	0.03	0.022	0.026	0.026	0.002309	4.037267081	95.96273292	
	0.25	0.025	0.029	0.031	0.028333	0.001764	4.399585921	95.60041408	
	0.125	0.25	0.215	0.238	0.234333	0.010269	36.38716356	63.61283644	
	0.062	0.536	0.411	0.357	0.434667	0.05301	67.49482402	32.50517598	
	0.031	0.627	0.597	0.599	0.607667	0.009684	94.35817805	5.641821946	
2 [7NPs]	1	0.018	0.017	0.019	0.018	0.000577	2.795031056	97.20496894	0.04 ± 0
	0.5	0.018	0.015	0.018	0.017	0.001	2.639751553	97.36024845	
	0.25	0.019	0.019	0.017	0.018333	0.000667	2.84679089	97.15320911	
	0.125	0.03	0.028	0.034	0.030667	0.001764	4.761904762	95.23809524	
	0.062	0.217	0.185	0.22	0.207333	0.0112	32.19461698	67.80538302	
	0.031	0.421	0.37	0.389	0.393333	0.014881	61.07660455	38.92339545	
3 [5NPs]	1	0.018	0.015	0.015	0.016	0.001	2.48447205	97.51552795	0.07 ± 0
	0.5	0.016	0.019	0.016	0.017	0.001	2.639751553	97.36024845	
	0.25	0.017	0.02	0.019	0.018667	0.000882	2.898550725	97.10144928	
	0.125	0.022	0.021	0.027	0.023333	0.001856	3.623188406	96.37681159	
	0.062	0.271	0.243	0.259	0.257667	0.00811	40.01035197	59.98964803	
	0.031	0.562	0.54	0.578	0.56	0.011015	86.95652174	13.04347826	
4 [6NPs]	1	0.016	0.018	0.019	0.017667	0.000882	2.743271222	97.25672878	0.07 ± 0
	0.5	0.019	0.019	0.023	0.020333	0.001333	3.157349896	96.8426501	
	0.25	0.022	0.026	0.031	0.026333	0.002603	4.089026915	95.91097308	
	0.125	0.03	0.029	0.033	0.030667	0.001202	4.761904762	95.23809524	
	0.062	0.277	0.291	0.256	0.274667	0.010171	42.65010352	57.34989648	
	0.031	0.626	0.651	0.63	0.635667	0.007753	98.70600414	1.293995859	
5 [4NPs]	1	0.018	0.017	0.019	0.018	0.000577	2.795031056	97.20496894	0.04 ± 0
	0.5	0.022	0.019	0.028	0.023	0.002646	3.571428571	96.42857143	
	0.25	0.02	0.019	0.022	0.020333	0.000882	3.157349896	96.8426501	
	0.125	0.022	0.04	0.037	0.033	0.005568	5.124223602	94.8757764	
	0.062	0.082	0.099	0.106	0.095667	0.007126	14.85507246	85.14492754	
	0.031	0.351	0.401	0.375	0.375667	0.014438	58.33333333	41.66666667	
6 [8NPs]	1	0.019	0.017	0.019	0.018333	0.000667	2.84679089	97.15320911	0.07 ± 0
	0.5	0.018	0.02	0.02	0.019333	0.000667	3.002070393	96.99792961	
	0.25	0.022	0.017	0.021	0.02	0.001528	3.105590062	96.89440994	
	0.125	0.041	0.038	0.03	0.036333	0.003283	5.641821946	94.35817805	
	0.062	0.321	0.284	0.319	0.308	0.012014	47.82608696	52.17391304	
	0.031	0.538	0.541	0.519	0.532667	0.006888	82.71221532	17.28778468	

ID	uM/ml	O.D			Mean O.D	±SE	Viability %	Toxicity %	IC50 ± SD
**A549**	--------	0.638	0.641	0.653	0.644	0.004583	100	0	uM
**Old 1** [7]	10	0.02	0.019	0.023	0.020667	0.001202	3.209109731	96.79089027	4.16 ± 0.07
	5	0.218	0.193	0.225	0.212	0.009713	32.91925466	67.08074534	
	2.5	0.583	0.623	0.618	0.608	0.012583	94.40993789	5.590062112	
	1.25	0.633	0.619	0.62	0.624	0.004509	96.89440994	3.105590062	
	0.625	0.656	0.615	0.624	0.631667	0.012441	98.08488613	1.915113872	
	0.312	0.618	0.609	0.633	0.62	0.007	96.27329193	3.726708075	
**Old 2** [8]	10	0.03	0.028	0.026	0.028	0.001155	4.347826087	95.65217391	2.3 ± 0.06
	5	0.022	0.046	0.031	0.033	0.007	5.124223602	94.8757764	
	2.5	0.271	0.294	0.256	0.273667	0.01105	42.49482402	57.50517598	
	1.25	0.641	0.639	0.64	0.64	0.000577	99.37888199	0.621118012	
	0.625	0.656	0.624	0.638	0.639333	0.009262	99.27536232	0.724637681	
	0.312	0.63	0.646	0.632	0.636	0.005033	98.75776398	1.242236025	
**Old 3** [4]	10	0.02	0.022	0.02	0.020667	0.000667	3.209109731	96.79089027	1.53 ± 0.01
	5	0.02	0.033	0.028	0.027	0.003786	4.192546584	95.80745342	
	2.5	0.063	0.071	0.068	0.067333	0.002333	10.45548654	89.54451346	
	1.25	0.317	0.335	0.327	0.326333	0.005207	50.67287785	49.32712215	
	0.625	0.656	0.629	0.647	0.632	0.007937	98.13664596	1.863354037	
	0.312	0.633	0.65	0.619	0.634	0.008963	98.44720497	1.552795031	
**Old 4** [5]	10	0.072	0.091	0.088	0.083667	0.005897	12.99171843	87.00828157	4.27 ± 0.08
	5	0.261	0.245	0.271	0.259	0.007572	40.2173913	59.7826087	
	2.5	0.489	0.472	0.458	0.473	0.008963	73.44720497	26.55279503	
	1.25	0.618	0.602	0.594	0.604667	0.007055	93.89233954	6.107660455	
	0.625	0.644	0.631	0.639	0.632	0.003786	98.13664596	1.863354037	
	0.312	0.641	0.65	0.637	0.642667	0.003844	99.79296066	0.207039337	
**Old 5** [6]	10	0.017	0.018	0.017	0.017333	0.000333	2.691511387	97.30848861	1.14 ± 0.04
	5	0.042	0.049	0.062	0.051	0.005859	7.919254658	92.08074534	
	2.5	0.088	0.081	0.088	0.085667	0.002333	13.30227743	86.69772257	
	1.25	0.241	0.289	0.271	0.267	0.014	41.45962733	58.54037267	
	0.625	0.61	0.582	0.597	0.632	0.00809	98.13664596	1.863354037	
	0.312	0.631	0.646	0.64	0.639	0.004359	99.22360248	0.776397516	
**Schif** [9]	10	0.02	0.02	0.024	0.021333	0.001333	3.3126294	96.6873706	3.43 ± 0.05
	5	0.081	0.108	0.115	0.101333	0.010366	15.73498965	84.26501035	
	2.5	0.427	0.459	0.451	0.445667	0.009615	69.20289855	30.79710145	
	1.25	0.647	0.632	0.636	0.638333	0.004485	99.12008282	0.879917184	
	0.625	0.628	0.619	0.627	0.632	0.002848	98.13664596	1.863354037	
	0.312	0.633	0.645	0.65	0.642667	0.005044	99.79296066	0.207039337	

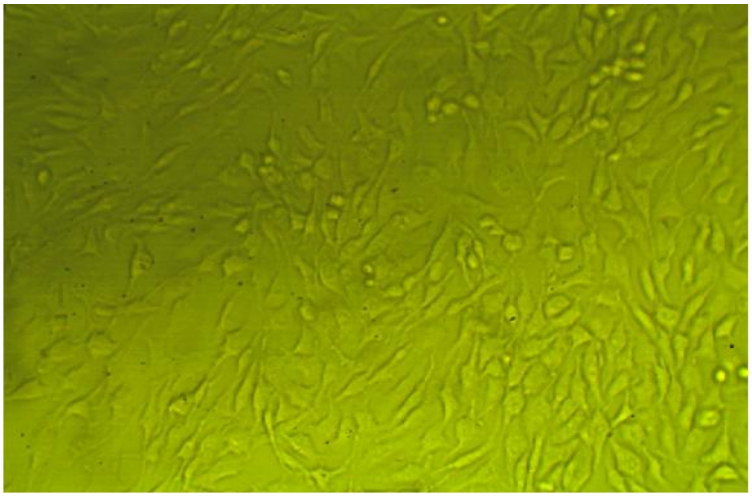

control
A549 cells

Organism : *Homo sapiens*, human
Tissue : lung
Cell Type : epithelial
Culture Properties : adherent
Disease : Carcinoma
ATCC : CCL-185

Effect of sample Se NPs on A549 cells at different concentration

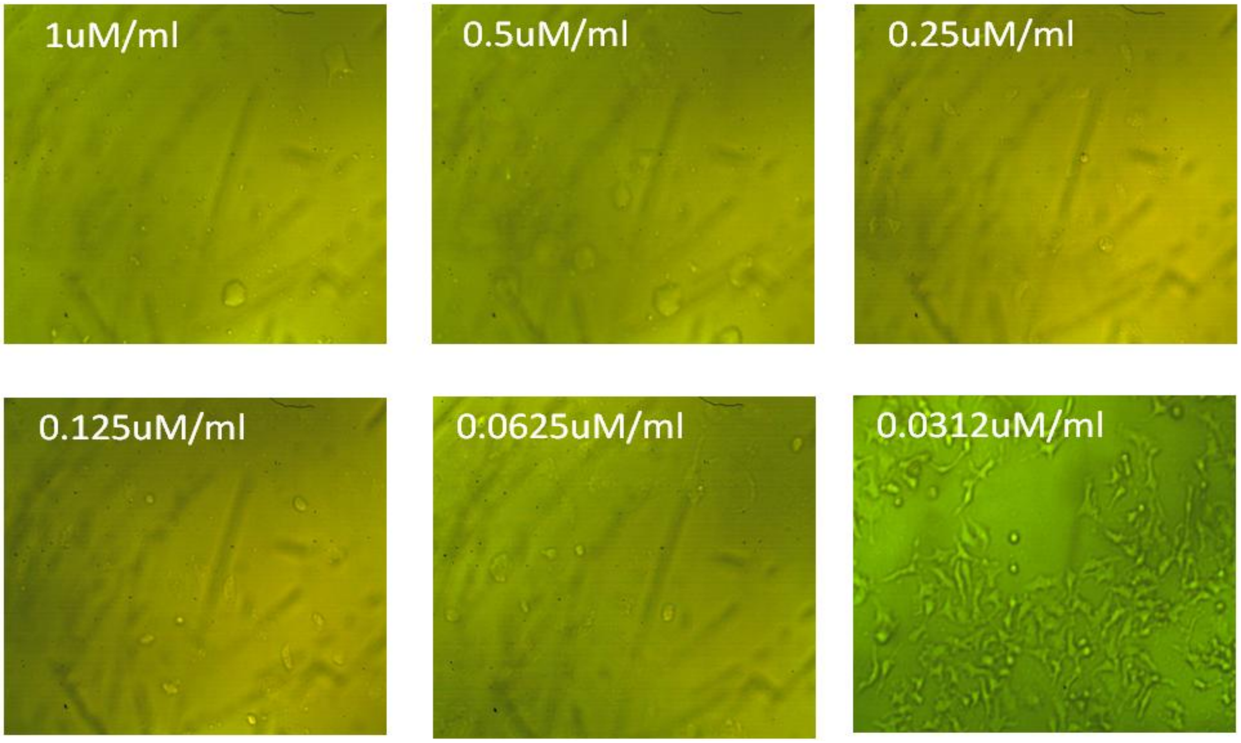

Effect of sample 1 on A549 cells at different concentration

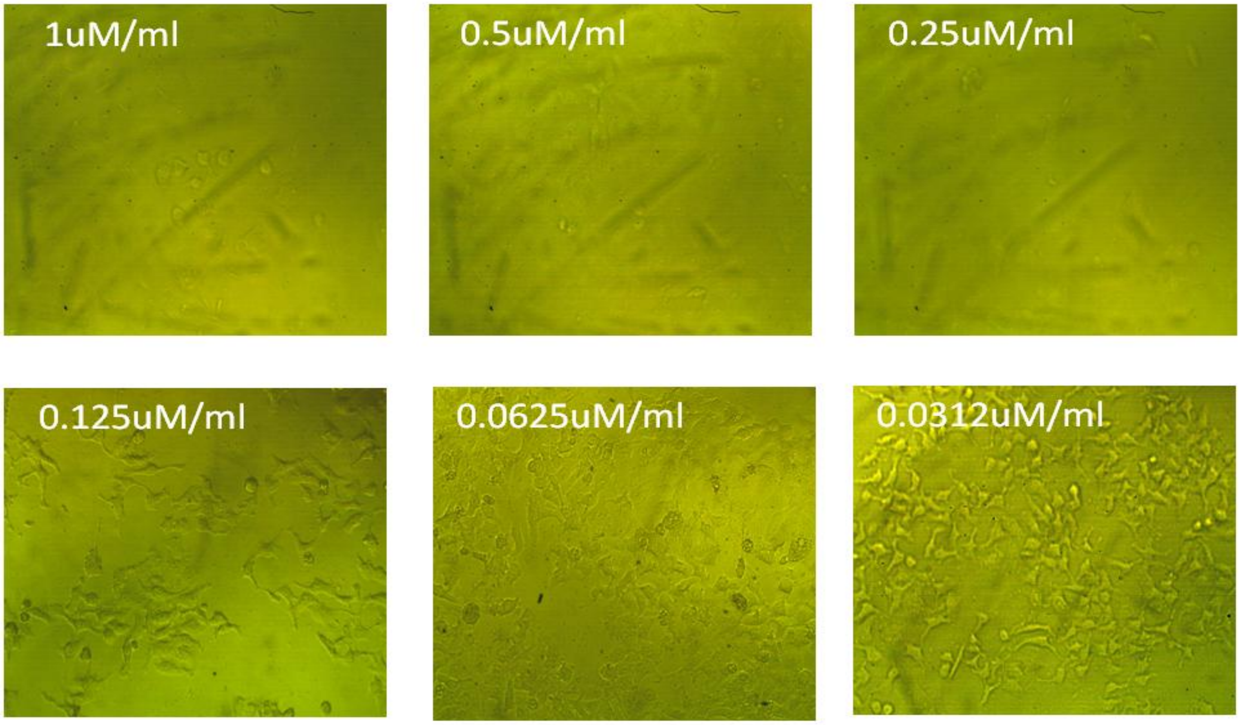

Effect of sample 2 on A549 cells at different concentration

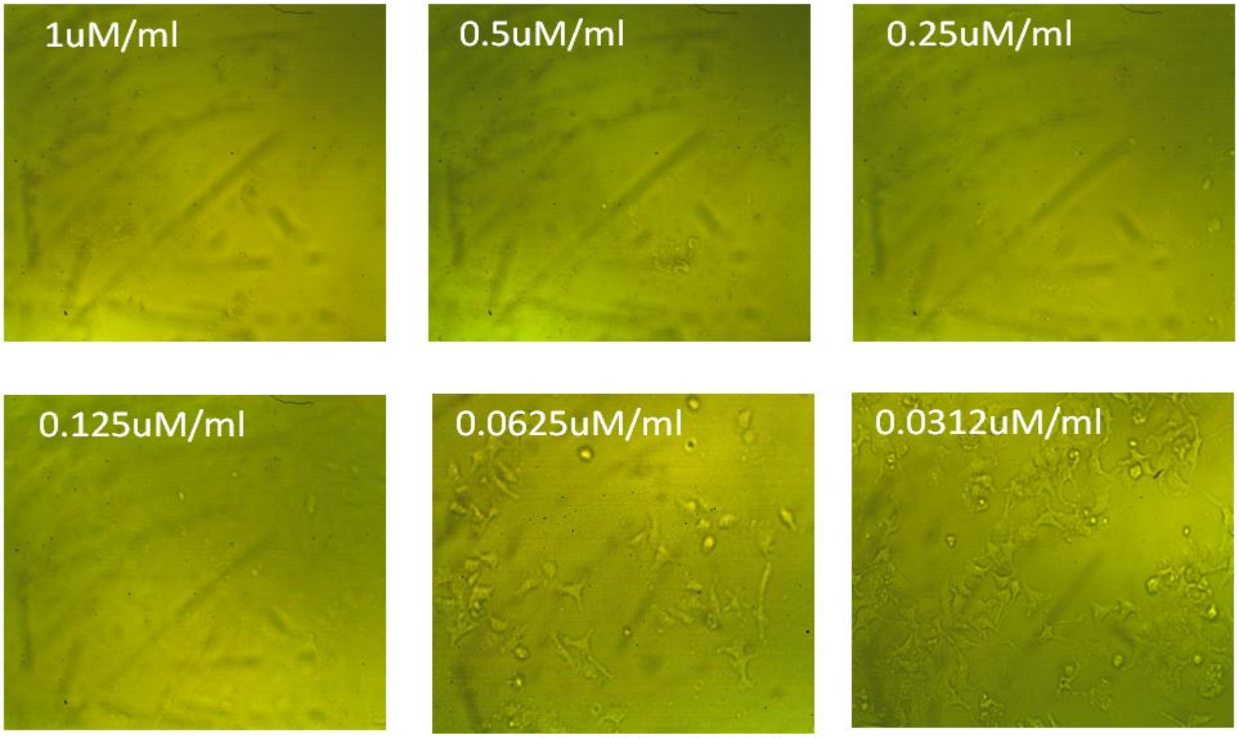

Effect of sample 3 on A549 cells at different concentration

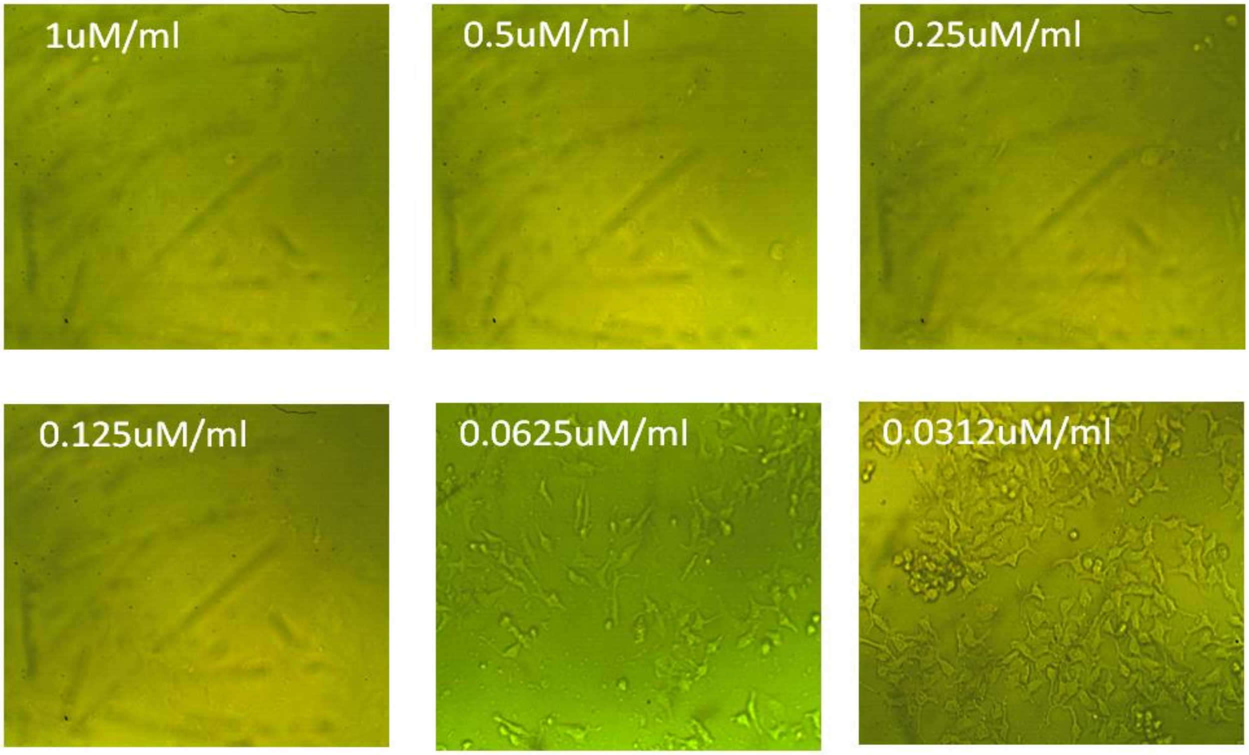

Effect of sample 4 on A549 cells at different concentration

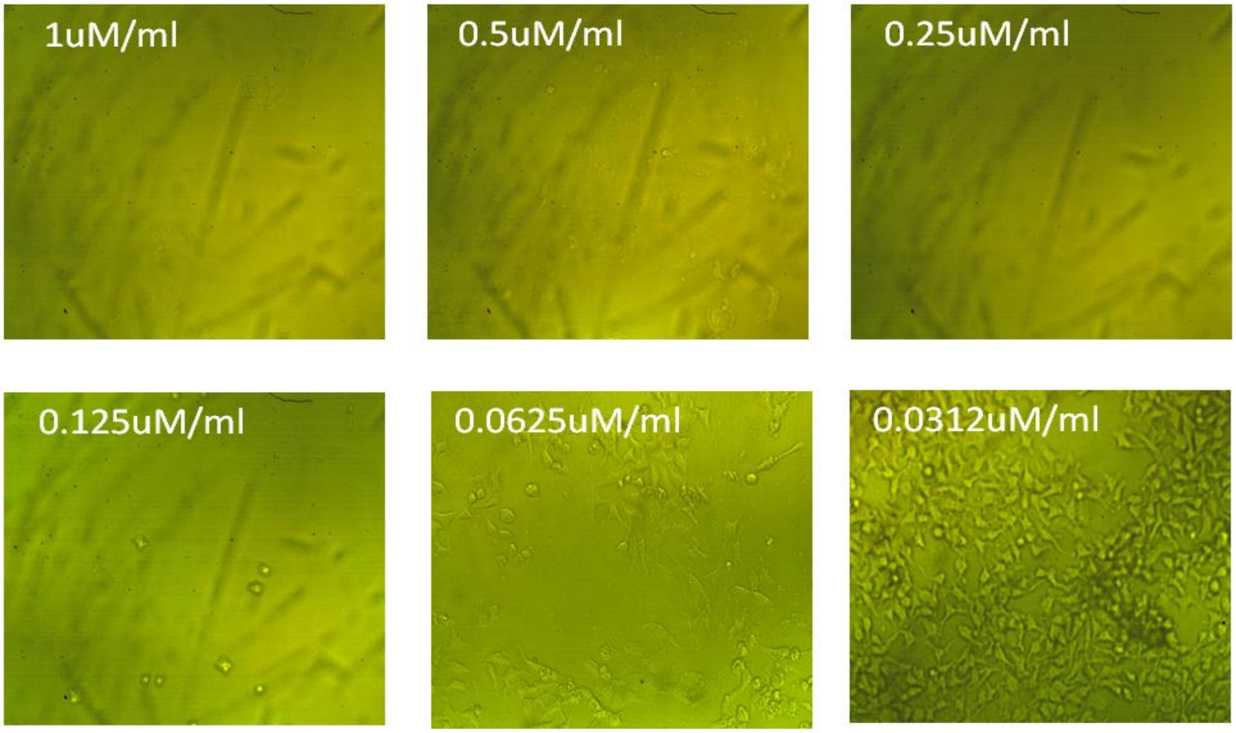

Effect of sample 5 on A549 cells at different concentration

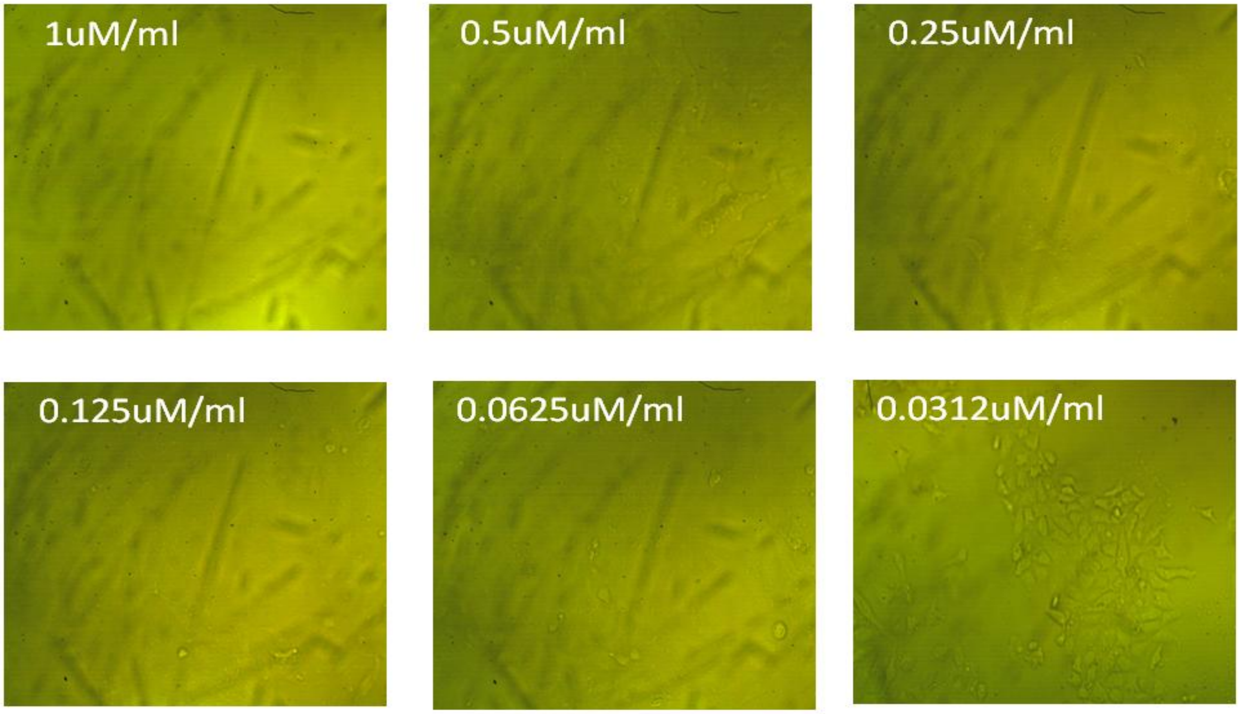

Effect of sample 6 on A549 cells at different concentration

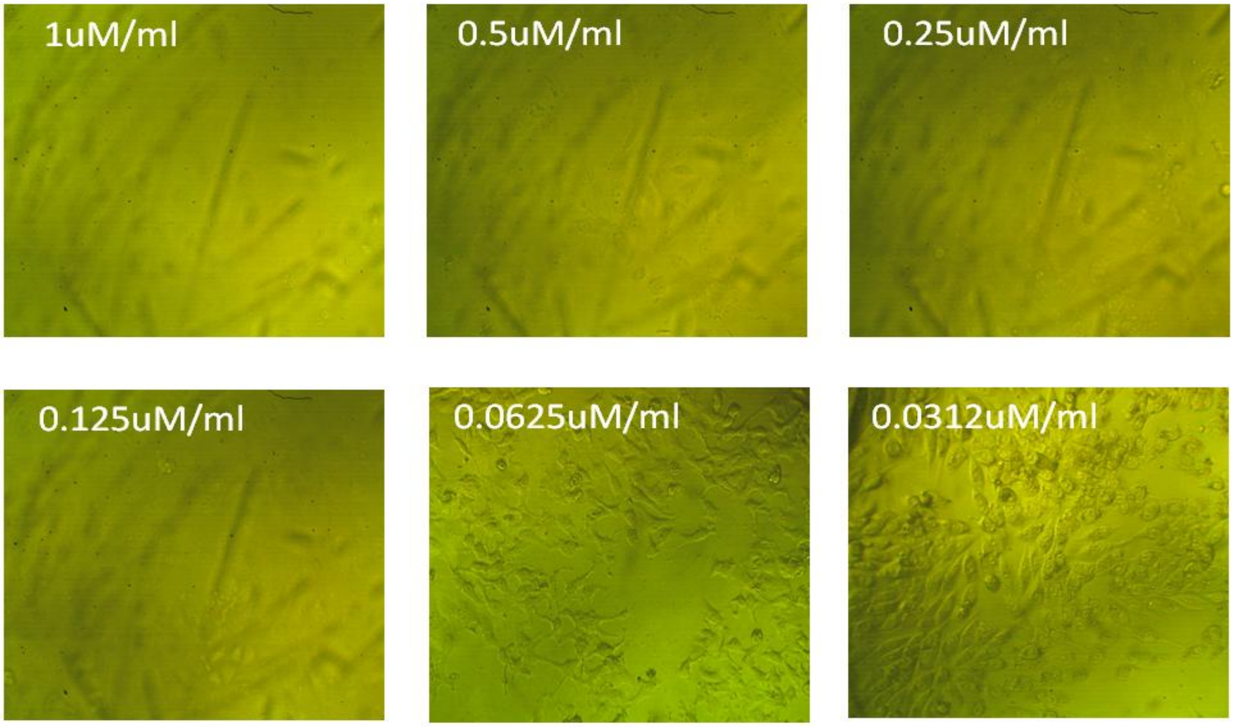

Effect of sample Old 1 on A549 cells at different concentration

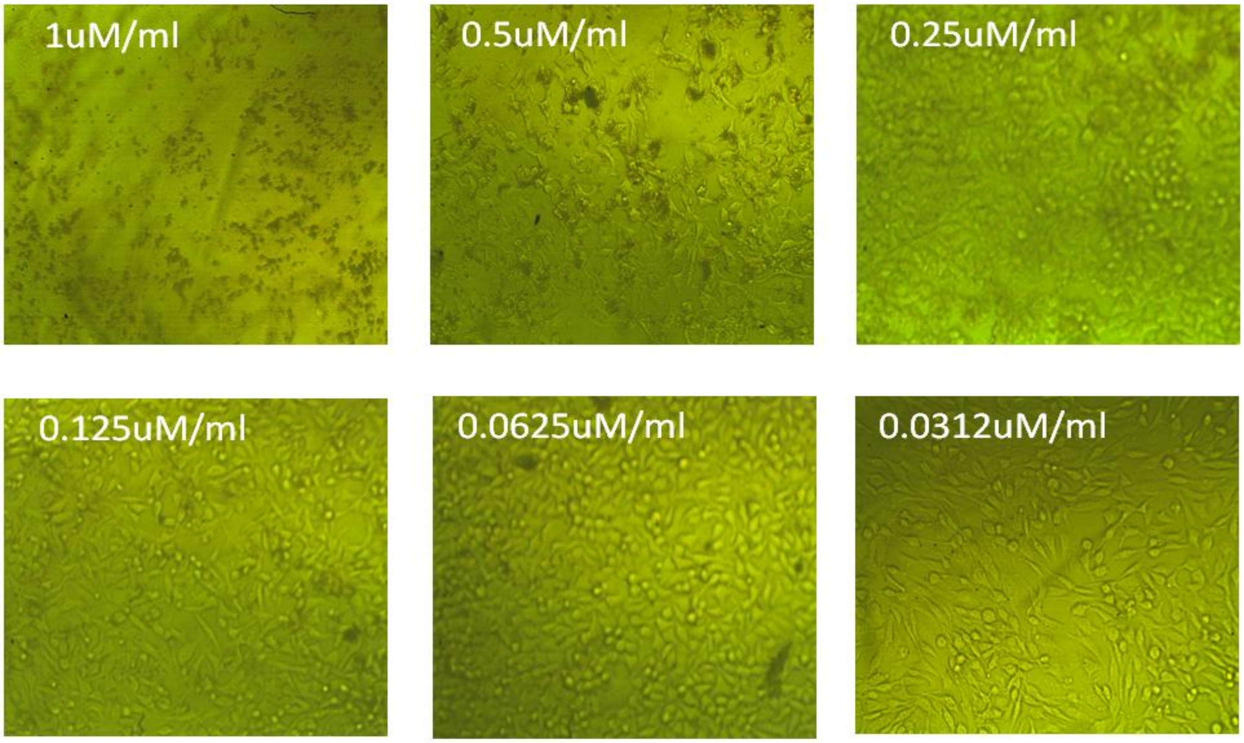

Effect of sample Old 2 on A549 cells at different concentration

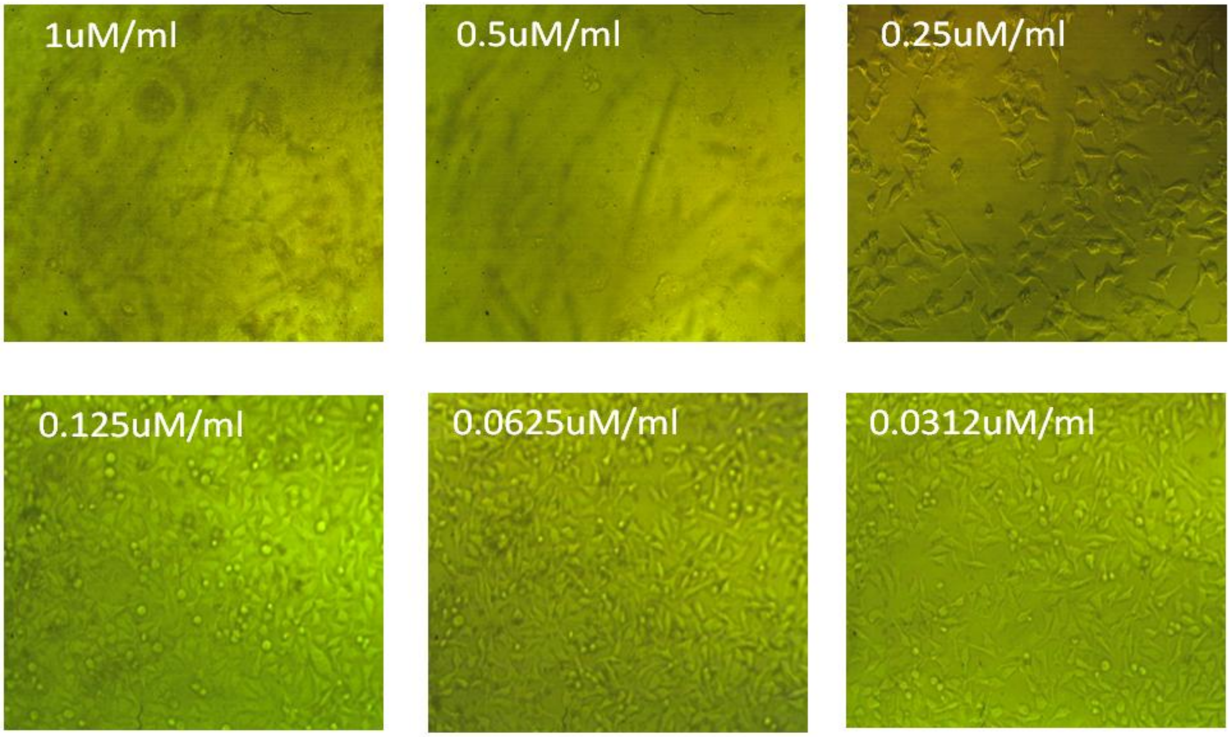

Effect of sample Old 3 on A549 cells at different concentration

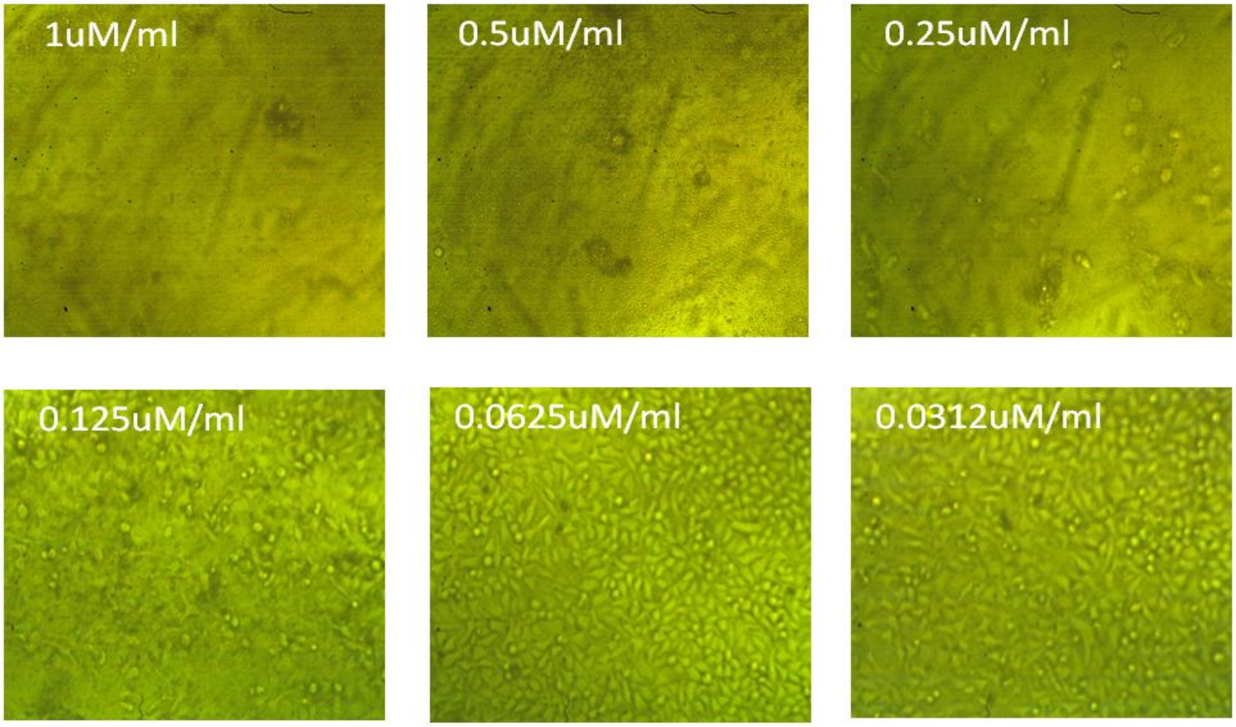

Effect of sample Old 4 on A549 cells at different concentration

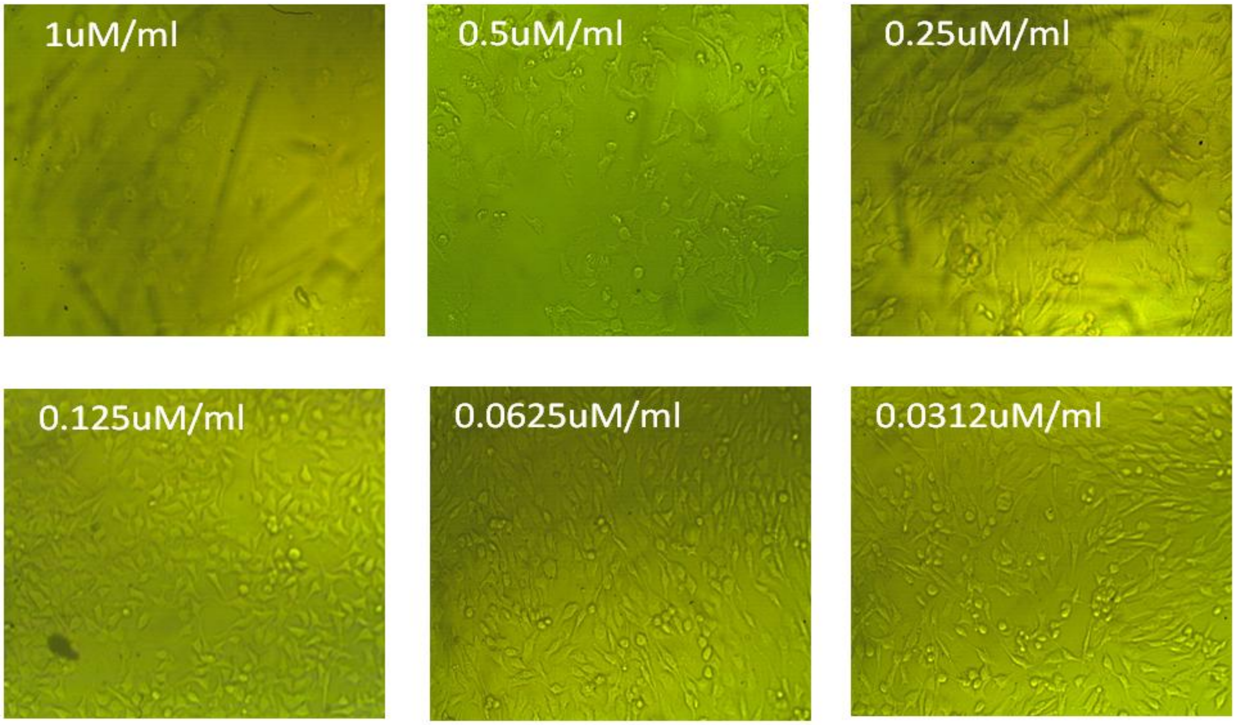

Effect of sample Old 5 on A549 cells at different concentration

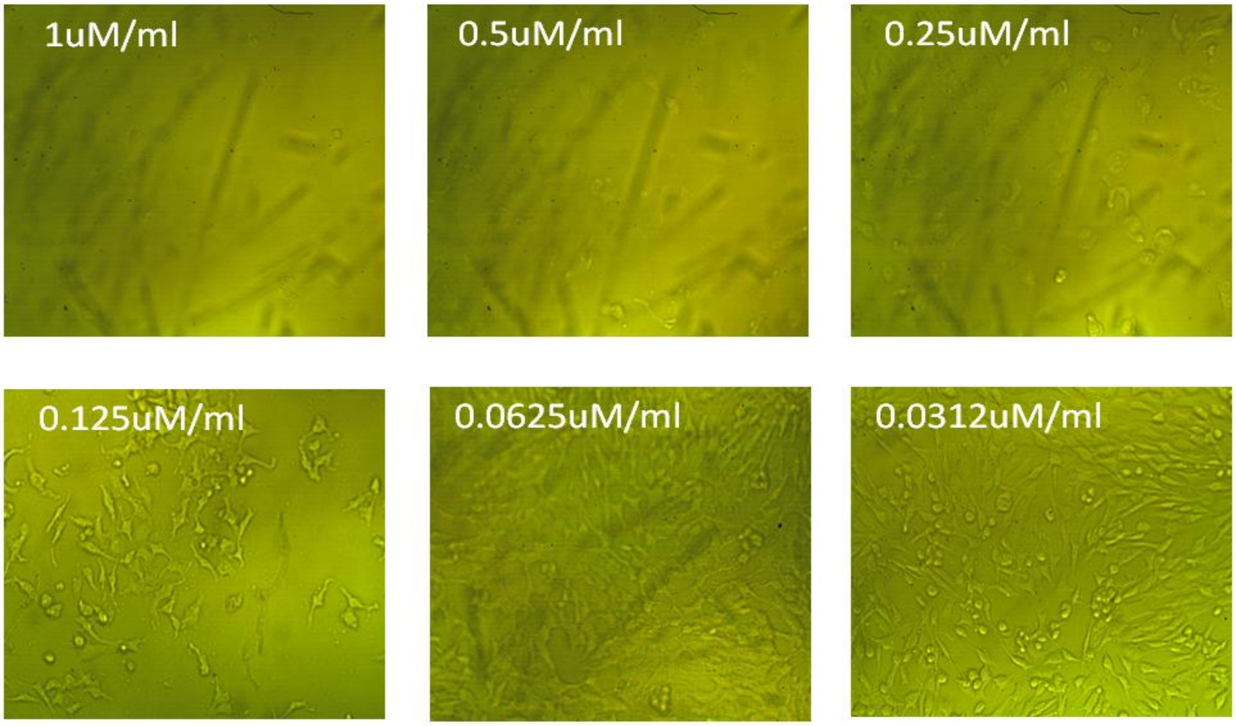

Effect of sample Schif on A549 cells at different concentration

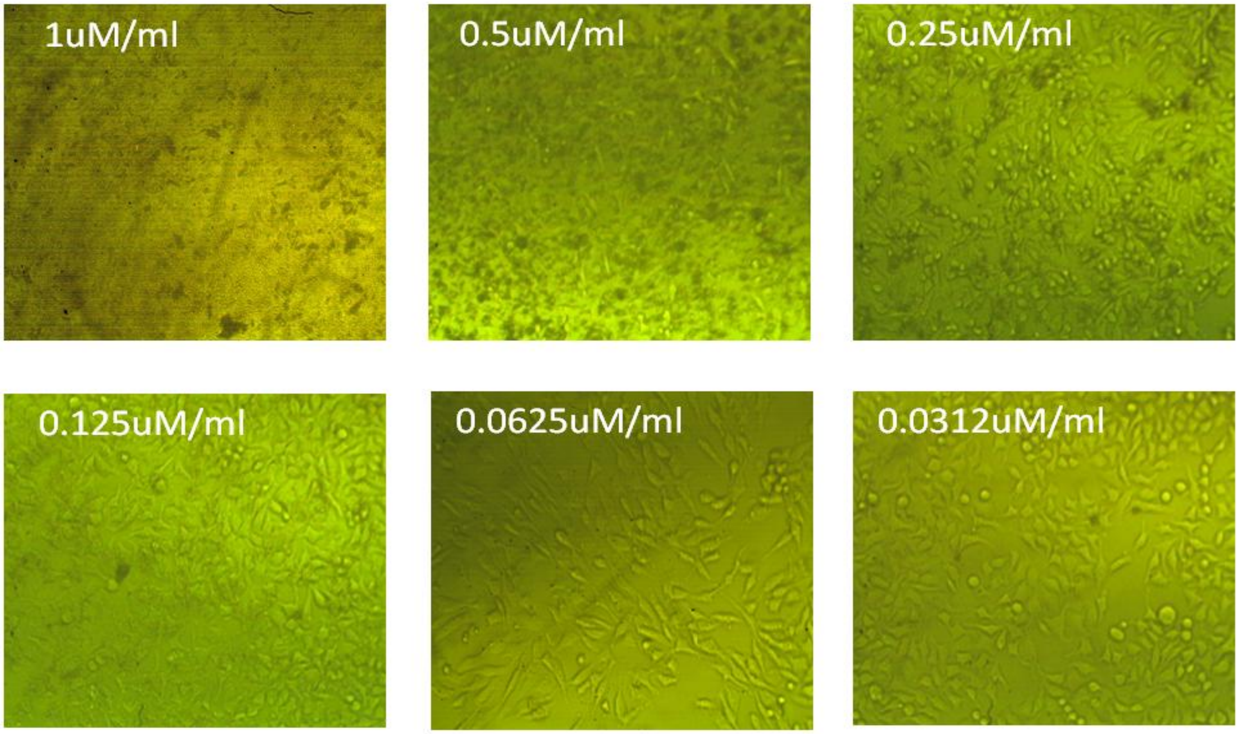

Test code: T-1-023-32

samples number : 4

experiment design : effect against Vero cells

ID	uM/ml	O.D			Mean O.D	±SE	Viability %	Toxicity %	IC50 ± SD
Vero	--------	0.772	0.79	0.778	0.78	0.005292	100	0	uM
4 [6NPs]	1	0.037	0.022	0.024	0.027667	0.004702	3.547008547	96.45299145	0.35 ± 0.01
	0.5	0.228	0.251	0.269	0.249333	0.011865	31.96581197	68.03418803	
	0.25	0.413	0.384	0.409	0.402	0.009074	51.53846154	48.46153846	
	0.125	0.764	0.781	0.773	0.772667	0.00491	99.05982906	0.94017094	
	0.062	0.777	0.784	0.771	0.777333	0.003756	99.65811966	0.341880342	
	0.031	0.789	0.764	0.772	0.775	0.007371	99.35897436	0.641025641	
5 [4NPs]	1	0.073	0.046	0.055	0.058	0.007937	7.435897436	92.56410256	0.59 ± 0.01
	0.5	0.41	0.388	0.393	0.397	0.006658	50.8974359	49.1025641	
	0.25	0.724	0.756	0.749	0.743	0.009713	95.25641026	4.743589744	
	0.125	0.771	0.794	0.768	0.777667	0.008212	99.7008547	0.299145299	
	0.062	0.776	0.785	0.776	0.779	0.003	99.87179487	0.128205128	
	0.031	0.78	0.764	0.782	0.775333	0.005696	99.4017094	0.598290598	
Old 3 [4]	10	0.052	0.048	0.042	0.047333	0.002906	6.068376068	93.93162393	4 ± 0.05
	5	0.327	0.348	0.336	0.337	0.006083	43.20512821	56.79487179	
	2.5	0.462	0.489	0.471	0.474	0.007937	60.76923077	39.23076923	
	1.25	0.768	0.782	0.779	0.776333	0.004256	99.52991453	0.47008547	
	0.62	0.775	0.772	0.78	0.775667	0.002333	99.44444444	0.555555556	
	0.31	0.762	0.779	0.777	0.772667	0.005364	99.05982906	0.94017094	
Old 5 [6]	10	0.084	0.099	0.073	0.085333	0.007535	10.94017094	89.05982906	3.34 ± 0.06
	5	0.188	0.219	0.204	0.203667	0.00895	26.11111111	73.88888889	
	2.5	0.4	0.38	0.427	0.402333	0.013618	51.58119658	48.41880342	
	1.25	0.783	0.751	0.764	0.766	0.009292	98.20512821	1.794871795	
	0.62	0.774	0.787	0.776	0.779	0.004041	99.87179487	0.128205128	
	0.31	0.771	0.782	0.779	0.777333	0.003283	99.65811966	0.341880342	

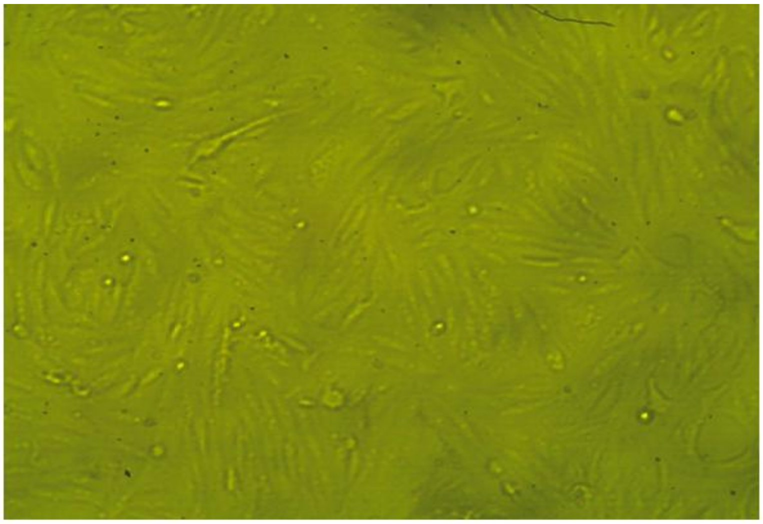

control vero cells

Organism :	*Cercopithecus aethiops*
Tissue :	kidney
Cell Type :	epithelial
Culture Properties :	adherent
Disease :	normal
ATCC :	CCL-81

Effect of sample 4 on vero cells at different concentration

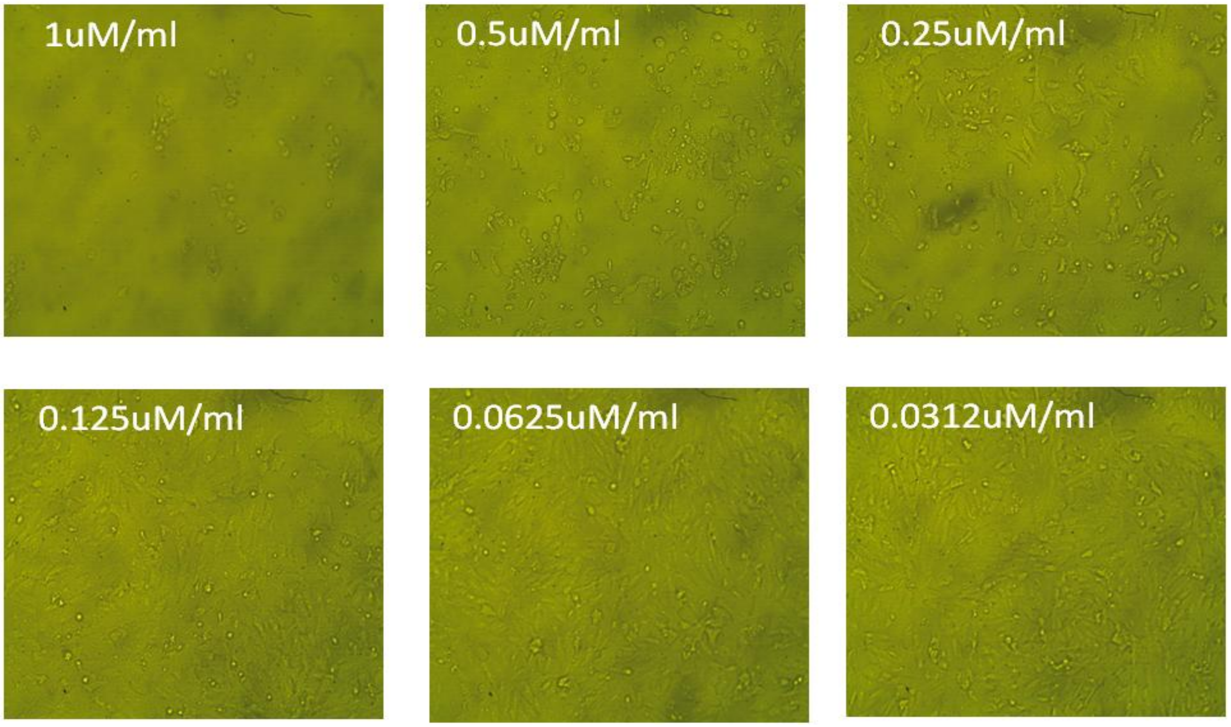

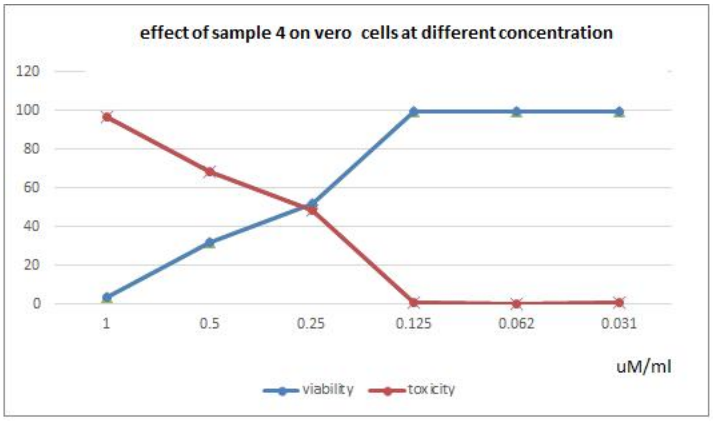

Effect of sample 5 on vero cells at different concentration

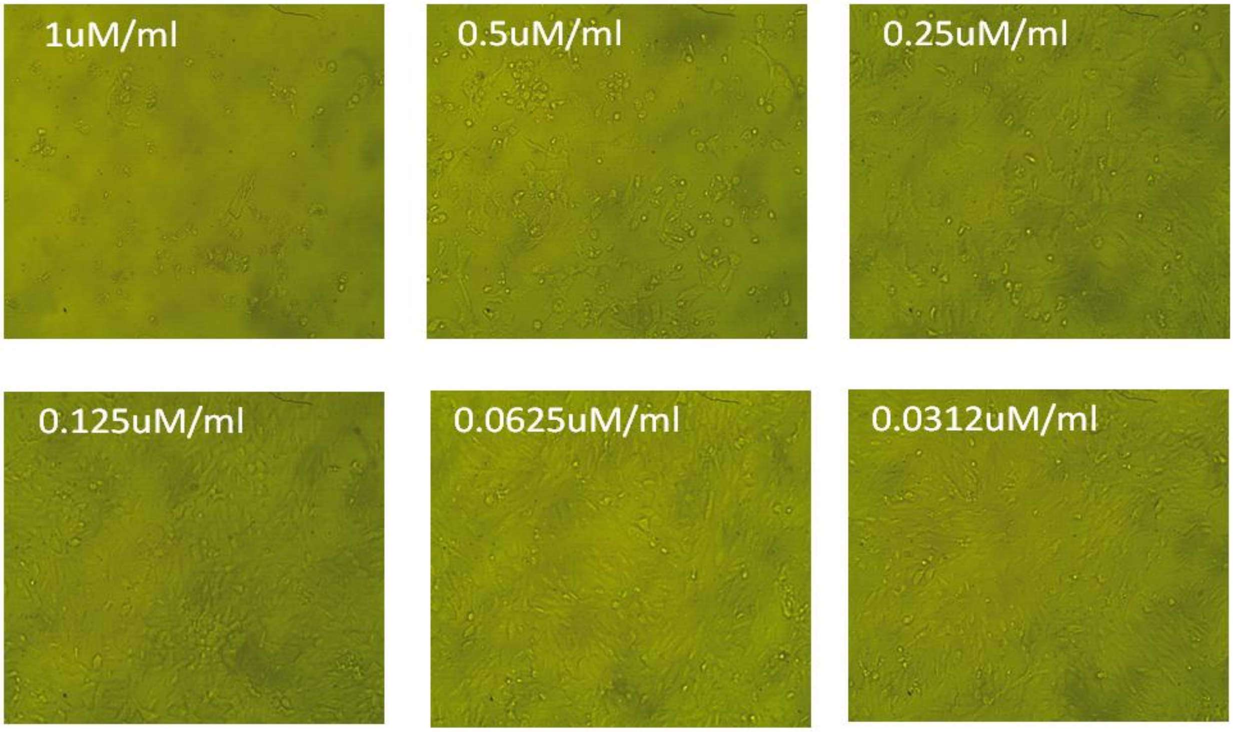

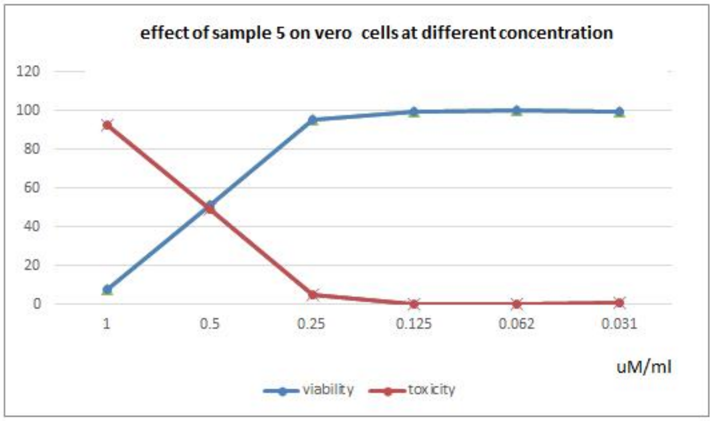

Effect of sample Old 3 on vero cells at different concentration

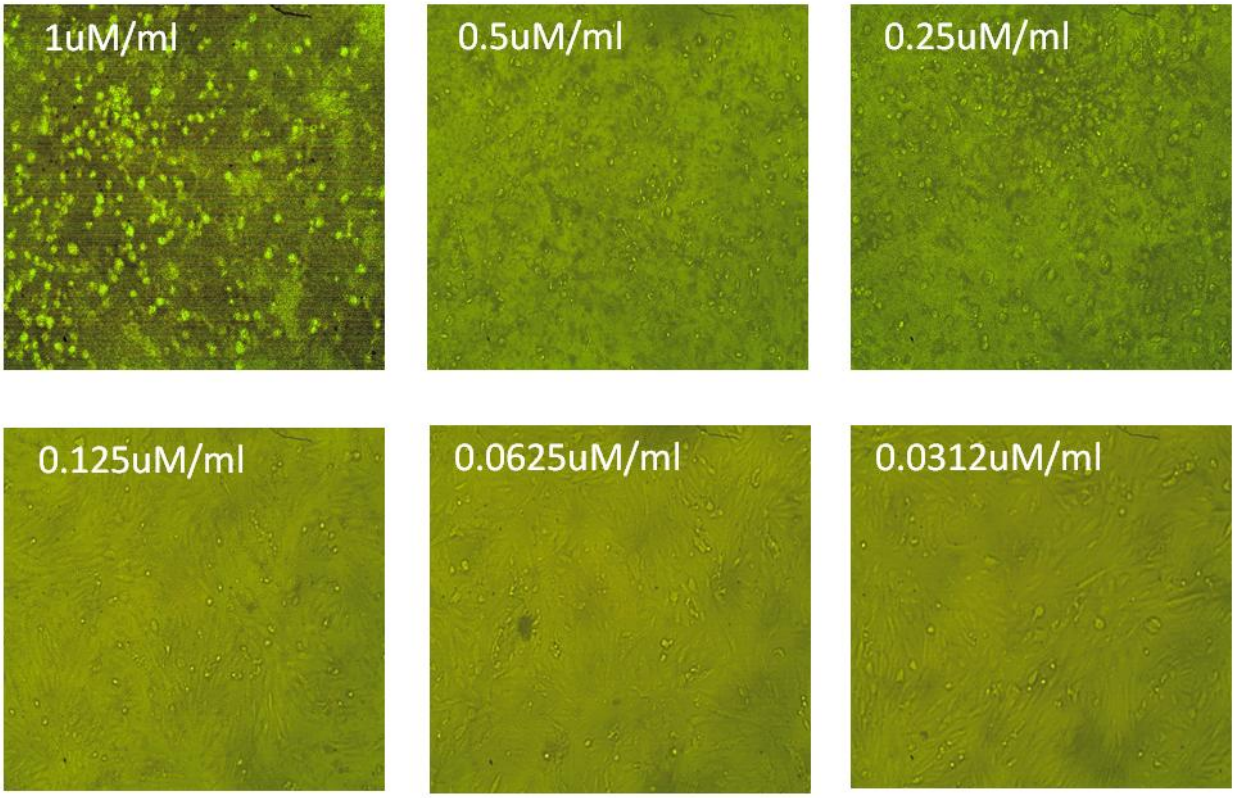

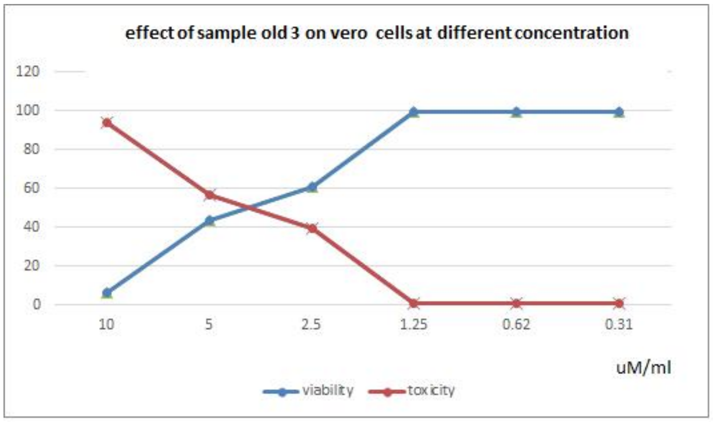

Effect of sample Old 5 on vero cells at different concentration

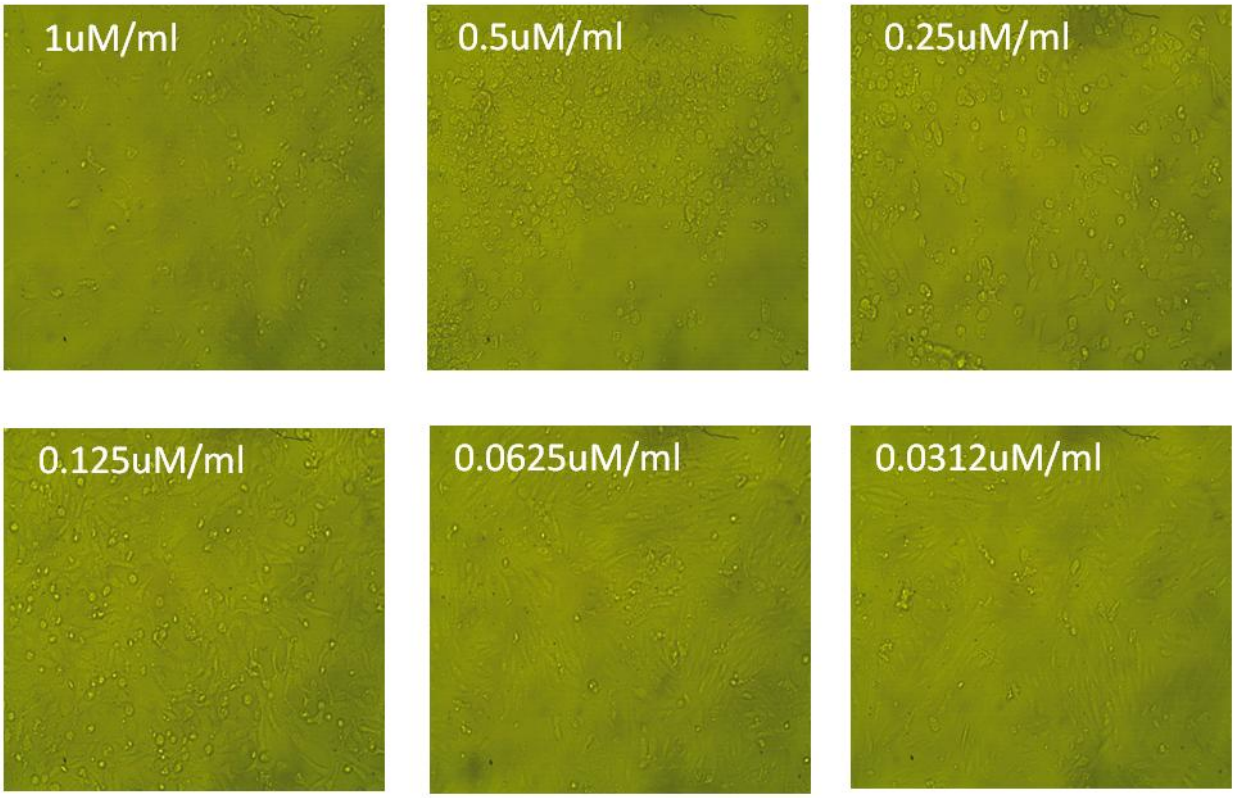

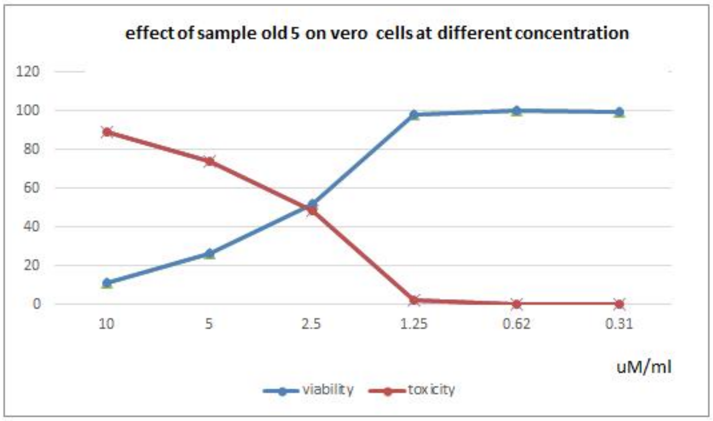

Test code: T- 5FU

samples number : 1

experiment design : effect of 5-FU against MCF-7, HepG2, A549, and Vero cells

ID	Conc. uM	O.D			Mean O.D	ST.E	Viability %	Toxicity %	IC50%
MCF-7	--------	0.639	0.651	0.645	0.645	0.003464	100	0	uM
5-FU.	10	0.026	0.041	0.037	0.034667	0.004485	5.374677003	94.625323	1.82 ± 0.03
	5	0.044	0.062	0.058	0.054667	0.005457	8.475452196	91.5245478	
	2.5	0.152	0.168	0.182	0.167333	0.008667	25.94315245	74.05684755	
	1.25	0.427	0.418	0.442	0.429	0.007	66.51162791	33.48837209	
	0.625	0.643	0.65	0.629	0.640667	0.006173	99.32816537	0.671834625	
	0.312	0.651	0.647	0.631	0.643	0.00611	99.68992248	0.310077519	

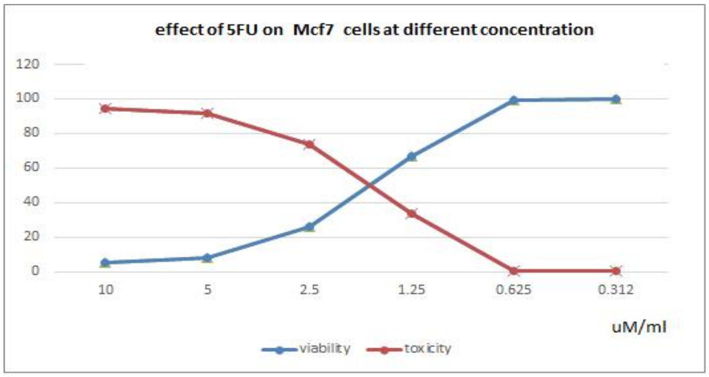

ID	Conc. uM	O.D			Mean O.D	ST.E	Viability %	Toxicity %	IC50%
HepG-2	--------	0.752	0.781	0.765	0.766	0.008386	100	0	uM
5-FU	10	0.017	0.019	0.018	0.018	0.000577	2.349869452	97.65013055	1.36 ± 0.01
	5	0.019	0.025	0.021	0.021667	0.001764	2.828546562	97.17145344	
	2.5	0.063	0.092	0.082	0.079	0.008505	10.31331593	89.68668407	
	1.25	0.341	0.318	0.32	0.326333	0.007356	42.60226284	57.39773716	
	0.625	0.638	0.652	0.658	0.649333	0.005925	84.76936466	15.23063534	
	0.312	0.753	0.768	0.761	0.760667	0.004333	99.30374238	0.696257615	

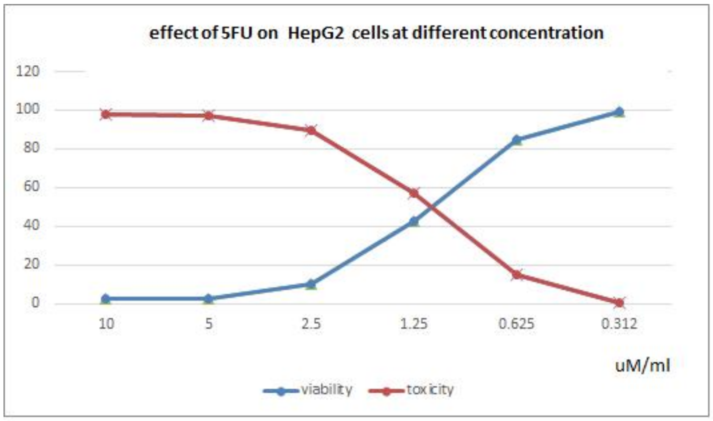

ID	Conc. uM	O.D			Mean O.D	ST.E	Viability %	Toxicity %	IC50%
A549	--------	0.693	0.672	0.669	0.678	0.00755	100	0	uM
5-FU.	10	0.019	0.017	0.019	0.018333	0.000667	2.704031465	97.29596853	0.76 ± 0.04
	5	0.02	0.018	0.024	0.020667	0.001764	3.048180924	96.95181908	
	2.5	0.053	0.027	0.052	0.044	0.008505	6.489675516	93.51032448	
	1.25	0.126	0.148	0.153	0.142333	0.008293	20.99311701	79.00688299	
	0.625	0.388	0.401	0.392	0.393667	0.003844	58.06293019	41.93706981	
	0.312	0.683	0.661	0.679	0.674333	0.006766	99.45919371	0.540806293	

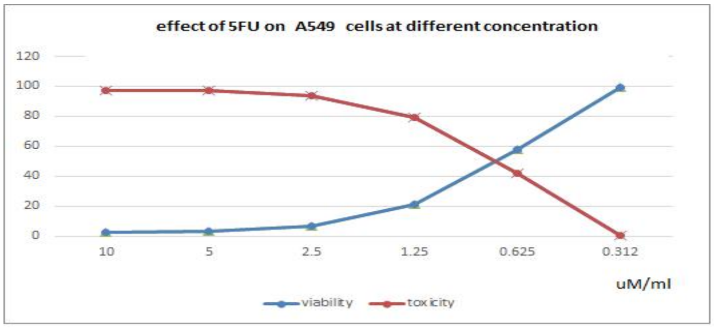

ID	Conc. uM	O.D			Mean O.D	ST.E	Viability %	Toxicity %	
Vero	--------	0.825	0.811	0.824	0.82	0.004509	100	0	uM
5-FU	10	0.02	0.051	0.037	0.036	0.008963	4.390243902	95.6097561	3.04 ± 0.06
	5	0.184	0.142	0.138	0.154667	0.014712	18.86178862	81.13821138	
	2.5	0.438	0.392	0.411	0.413667	0.013346	50.44715447	49.55284553	
	1.25	0.699	0.732	0.718	0.716333	0.009563	87.35772358	12.64227642	
	0.625	0.824	0.805	0.817	0.815333	0.005548	99.43089431	0.569105691	
	0.312	0.816	0.823	0.82	0.819667	0.002028	99.95934959	0.040650407	

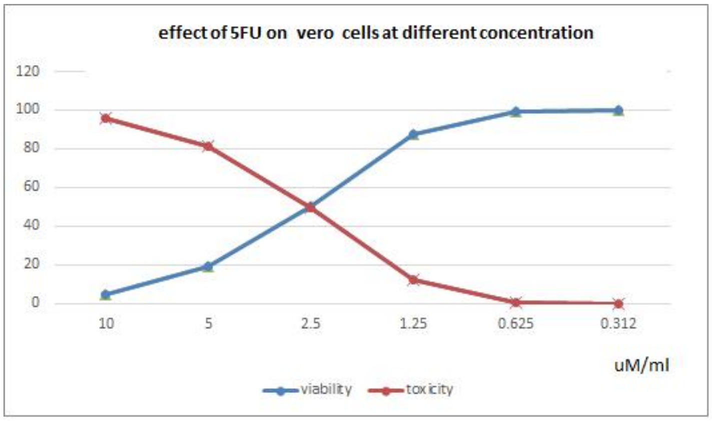

CDK1 Lab Report

cdk1

code	IC50	conc	log	%inh	T2	T1	ΔT	RFU2	RFU1	ΔRFU	slope	K.Activity
Ald3		100	2	86	30	0	30	13.69	0	13.69	3.33333	16.42802
		10	1	65	30	0	30	35.28	0	35.28	3.33333	42.33604
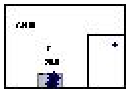		1	0	36	30	0	30	64.22	0	64.22	3.33333	77.06408
		0.1	-1	22	30	0	30	78.26	0	78.26	3.33333	93.91209
		0.01	-2	7.4	30	0	30	92.58	0	92.58	3.33333	111.0961
EC				0	30	0	30	100	0	100	3.33333	120

code	IC50	conc.ng/ml	log conc	%inh	T2	T1	ΔT	RFU2	RFU1	ΔRFU	slope	K.Activity
5 (nano)		100	2	91	30	0	30	8.76	0	8.76	3.33333	10.51201
		10	1	78	30	0	30	22.03	0	22.03	3.33333	26.43603
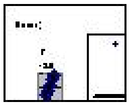		1	0	55	30	0	30	44.54	0	44.54	3.33333	53.44805
		0.1	-1	37	30	0	30	63.12	0	63.12	3.33333	75.74408
		0.01	-2	19	30	0	30	81.42	0	81.42	3.33333	97.7041
EC				0	30	0	30	100	0	100	3.33333	120

code	IC50	conc.ng/ml	log conc	%inh	T2	T1	ΔT	RFU2	RFU1	ΔRFU	slope	K.Activity
**Roscovitine**		100	2	93	30	0	30	6.69	0	6.69	3.33333	8.028008
		10	1	82	30	0	30	18.47	0	18.47	3.33333	22.16402
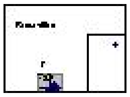		1	0	63	30	0	30	36.88	0	36.88	3.33333	44.25604
		0.1	-1	41	30	0	30	59.03	0	59.03	3.33333	70.83607
		0.01	-2	22	30	0	30	77.59	0	77.59	3.33333	93.10809
EC				0	30	0	30	100	0	100	3.33333	120

Colchicine binding assay Lab Report

CBS I

code	IC50	conc	log	%inh	T2	T1	ΔT	RFU2	RFU1	ΔRFU	slope	K.Activity
**Ald3**		100	2	87.05	30	0	30	991	0	991	255.03	15.5433
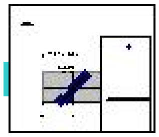		10	1	64.4	30	0	30	2724	0	2724	255.03	42.7244
		1	0	48.27	30	0	30	3958	0	3958	255.03	62.079
		0.1	-1	15.53	30	0	30	6463	0	6463	255.03	101.368

		0.01	-2	4.22	30	0	30	7328	0	7328	255.03	114.935
**[3H]-colchicine**				0	30	0	30	7651	0	7651	255.03	120
Background								254				

code	IC50	conc	log	%inh	T2	T1	ΔT	RFU2	RFU1	ΔRFU	slope	K.Activity
**5 (nano)**		100	2	91.36	30	0	30	661	0	661	255.03	10.3674
		10	1	76.19	30	0	30	1822	0	1822	255.03	28.577
		1	0	61.43	30	0	30	2951	0	2951	255.03	46.2848
		0.1	-1	28.86	30	0	30	5443	0	5443	255.03	85.3703
		0.01	-2	13.64	30	0	30	6607	0	6607	255.03	103.627
**[3H]-colchicine**				0	30	0	30	7651	0	7651	255.03	120
Background								254				

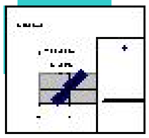

code	IC50	conc	log	%inh	T2	T1	ΔT	RFU2	RFU1	ΔRFU	slope	K.Activity
**CA4**		100	2	92.79	30	0	30	552	0	552	255.03	8.6578
		10	1	80.46	30	0	30	1495	0	1495	255.03	23.4482
		1	0	68.93	30	0	30	2377	0	2377	255.03	37.2819
		0.1	-1	43.97	30	0	30	4287	0	4287	255.03	67.2391
		0.01	-2	19.19	30	0	30	6183	0	6183	255.03	96.9768
**[3H]-colchicine**				0	30	0	30	7651	0	7651	255.03	120
Background								254				

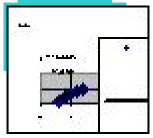

Supplement: Supplemental Material [file IENZ_A_2232125_SM1678.pdf]
